# Asymmetric Solvation of the Zinc Dimer Cation Revealed by Infrared Multiple Photon Dissociation Spectroscopy of Zn_2_^+^(H_2_O)*_n_* (*n* = 1–20)

**DOI:** 10.3390/ijms22116026

**Published:** 2021-06-02

**Authors:** Ethan M. Cunningham, Thomas Taxer, Jakob Heller, Milan Ončák, Christian van der Linde, Martin K. Beyer

**Affiliations:** Institut für Ionenphysik und Angewandte Physik, Universität Innsbruck, Technikerstraße 25, 6020 Innsbruck, Austria; thomas.taxer@uibk.ac.at (T.T.); jakob.heller@uibk.ac.at (J.H.); milan.oncak@uibk.ac.at (M.O.); christian.van-der-linde@uibk.ac.at (C.v.d.L.)

**Keywords:** infrared spectroscopy, metal dimer, photodissociation, hydrated metal ions, solvation evolution

## Abstract

Investigating metal-ion solvation—in particular, the fundamental binding interactions—enhances the understanding of many processes, including hydrogen production via catalysis at metal centers and metal corrosion. Infrared spectra of the hydrated zinc dimer (Zn_2_^+^(H_2_O)*_n_*; *n* = 1–20) were measured in the O–H stretching region, using infrared multiple photon dissociation (IRMPD) spectroscopy. These spectra were then compared with those calculated by using density functional theory. For all cluster sizes, calculated structures adopting asymmetric solvation to one Zn atom in the dimer were found to lie lower in energy than structures adopting symmetric solvation to both Zn atoms. Combining experiment and theory, the spectra show that water molecules preferentially bind to one Zn atom, adopting water binding motifs similar to the Zn^+^(H_2_O)*_n_* complexes studied previously. A lower coordination number of 2 was observed for Zn_2_^+^(H_2_O)_3_, evident from the highly red-shifted band in the hydrogen bonding region. Photodissociation leading to loss of a neutral Zn atom was observed only for *n* = 3, attributed to a particularly low calculated Zn binding energy for this cluster size.

## 1. Introduction

Metal–metal bonds have received much interest over the years; however, homonuclear metal bonds have received particular attention. There have been numerous experimental and theoretical studies on homonuclear transition metal dimers, including Zn_2_, with particular attention on the nature of the metal–metal bonding [1,2,3,4,5,6,7,8,9,10,11,12,13,14,15,16,17,18,19,20,21,22,23,24,25]. Atomic zinc, a group-12 metal, has an electron configuration of [Ar]3d^10^4s^2^, and because of its closed d-shell, it is often not considered a transition metal. Many chemical and spectroscopic properties of zinc are more similar to those of the alkaline earth metals when compared to other transition metals [26].

As Zn has a closed electronic configuration, the neutral dimer, Zn_2_, is only bound by weak van der Waals interactions [3]. Whereas the Zn_2_^−^ anion is considered to be unstable towards autodetachment [7]. The first compound containing a stable Zn–Zn bond came from the synthesis of decamethyldizincocene in 2004, which was heralded a “new frontier” for the Zn–Zn bond quoting a bond distance of 2.305 Å from X-ray measurements [17,27]. Andrews and co-workers employed matrix isolation to study HZnZnH, the infrared spectra of which were consistent with two equivalent Zn atoms [28]. Later, the same group discovered that the Zn–Zn bond can be stabilized by different isomeric forms of the same ligand: cyanide and isocyanide [29]. 

In 1988, Buckner et al. investigated the Zn_2_^+^ cation in the gas phase and discussed an ion-induced dipole bonding interaction between the two Zn atoms vs. covalent bonding with a bond order of one half [4]. Further reactivity investigations from the same study by Buckner showed that the Zn_2_^+^ displays rapid displacement of Zn, forming Zn^+^–molecule complexes, consistent with a weak binding interaction in Zn_2_^+^ [4]. In 2003, Gutsev et al. computed electronic and geometrical structures of homonuclear 3d neutral metal dimers, as well as the cations and anions [7]. Their results confirmed the suggestion of a 4s–4s bond in Zn_2_^+^, reporting a bond length of 2.60 Å and a value for the dissociation energy *D*_0_(Zn^+^–Zn) of 1.73 eV. In 2019, Tarento et al. conducted a theoretical study on charge localization in small group 12 clusters and found that Zn*_n_*^+^ (2 ≤ *n* ≤ 7) clusters have a regular, compact shape, with charge delocalized over the whole cluster [24]. A study on the fragmentation patterns of Zn_2_L*_n_*^+^ (L = pyrrole and furan, *n* = 1–5) was performed in 2005 by Wu et al. observing that the primary fragmentation routes are either the loss of a neutral Zn atom or loss of a single ligand molecule [12]. The loss of the neutral Zn atom becomes less likely with increasing ligand number, which the authors rationalize as decreasing ligand binding energy. More recently, the possibility of Zn=Zn double bonds in certain complexes has become an interest in theoretical chemistry [30,31].

In this work we present an infrared spectroscopic study of hydrated zinc dimer ions, Zn_2_^+^(H_2_O)*_n_*. Studying hydrated metal ions in the gas phase provides the opportunity to investigate solvation at a molecular level and to study various chemical processes such as hydrogen production via catalysis at metal centers [32] and corrosion effects [33] in a well-defined environment. Our group has an extended history in studying these systems [34,35,36,37,38,39,40,41,42,43,44,45,46,47,48,49,50,51]. Infrared action spectroscopy, coupled with quantum chemical calculations, is a powerful technique used to investigate the structures of gas-phase molecular complexes, [52,53,54,55,56,57,58,59,60] in particular hydrated metal ions [61,62,63,64,65,66,67,68,69]. We recently published a study investigating hydrated zinc monomer ions Zn^+^(H_2_O)*_n_* with up to 35 water molecules, [70] where we found evidence for surface solvation of Zn^+^, which was previously established by Duncan and co-workers for *n* ≤ 4 [71]. Here we investigate the solvation evolution of the zinc dimer cation up to 20 water molecules utilizing infrared multiple photon dissociation (IRMPD) spectroscopy. The focus of this study is to investigate how water molecules bind to Zn_2_^+^. Do water molecules bind symmetrically to both Zn atoms, or do they preferentially bind to one Zn atom, and at which cluster sizes are different binding sites observed? By size-selecting each complex, a detailed investigation answering such questions can be carried out. To the best of our knowledge, this is the first experimental IRMPD study on hydrated metal dimers. Going to large cluster sizes up to 20 water molecules, the evolution of infrared spectra with increasing cluster size is a first step towards understanding metal dimer solvation.

## 2. Results and Discussion

Infrared spectra of size-selected clusters were recorded via action spectroscopy, Reaction (1):

(1) $${\mathrm{Zn}}_{2}^{+}{\left(H_{2}O\right)}_{n}+m\;h\nu \to {\mathrm{Zn}}_{2}^{+}\left(H_{2}O{)}_{n-x}+x(H_{2}O\right)$$

However, for *n* = 3, a competing fragmentation channel was observed; photodissociation of the neutral zinc atom is represented by Reaction (2):

(2) $${\mathrm{Zn}}_{2}^{+}{\left(H_{2}O\right)}_{3}+m\;h\nu \to {\mathrm{Zn}}^{+}{\left(H_{2}O\right)}_{3}+\mathrm{Zn}$$

**Figure 1:**
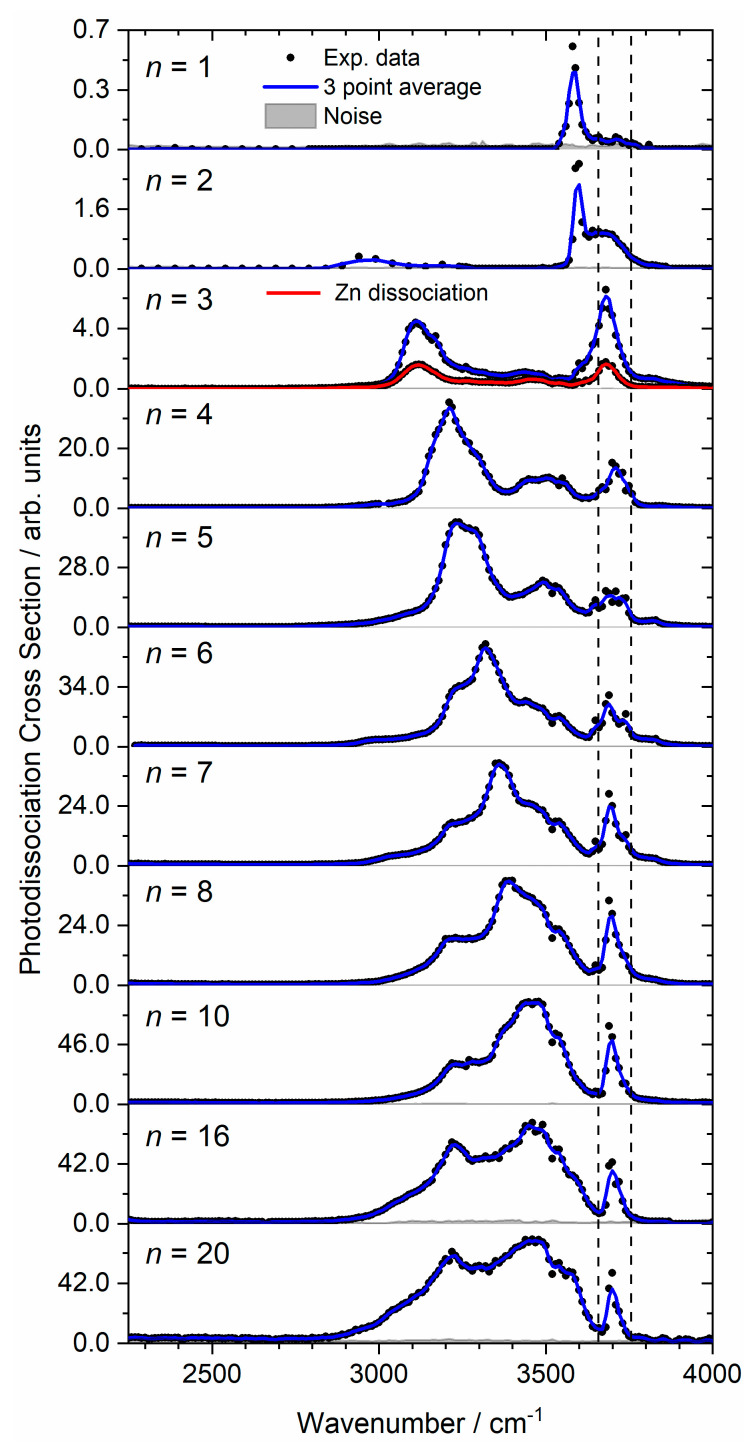
Experimental relative infrared photodissociation cross-section (arbitrary units) of Zn_2_^+^(H_2_O)*_n_* complexes (*n* = 1–8, 10, 16, and 20) assuming a one-photon process. In each case photodissociation events are due to loss of intact water molecules except for *n* = 3 where a second, less intense channel is observed due to Zn dissociation. The dashed lines in each experimental spectrum correspond to the wavenumbers of the symmetric and asymmetric stretching modes of isolated water at 3657 and 3756 cm^−1^, respectively [74].

Very weak photodissociation of neutral Zn + (H_2_O) was also observed for *n* = 4, most likely in a sequential reaction, initial H_2_O evaporation followed by Zn loss upon absorption of further IR photons. Figure 1 shows the experimental IRMPD photodissociation spectra of size-selected zinc dimer water complexes (Zn_2_^+^(H_2_O)*_n_*; *n* = 1–8, 10, 16, and 20), assuming a one-photon process for the calculated cross-sections. At least for the larger clusters, this is realistic, since the photon energy is close to the typical binding energy of a water molecule in water clusters—0.45 eV [72,73]. The spectrum of the smallest cluster, *n* = 1, presents one band centered at 3580 cm^−1^. For *n* = 2, the experimental spectrum is different to that of monatomic Zn^+^(H_2_O)_2_, investigated previously [70]. In the case of the dimer, there is a weak and broad red-shifted band at 2965 cm^−1^, along with an intense narrow band at 3590 cm^−1^, presenting a shoulder at 3680 cm^−1^. The *n* = 3 complex presents two different photodissociation channels: (i) loss of intact water molecules and (ii) loss of a neutral Zn atom (Figure 1, blue and red spectra, respectively). Both channels present similar spectra, showing two intense bands at 3110 and 3680 cm^−1^, with a weak feature centered at 3445 cm^−1^ (blue) and 3460 cm^−1^ (red). At *n* = 4, the most intense band is the red-shifted band centered at 3210 cm^−1^, along with weaker features centered at 3490 and 3710 cm^−1^. Similar to the zinc monomer–water complexes, [70] the bands at cluster sizes as low as *n* = 4 present increased spectral broadening, which signifies several isomers contributing to the experimental spectrum. A broad, unresolved red-shifted band at 3230 cm^−1^, along with a shoulder at 3270 cm^−1^ is presented for *n* = 5. This red-shifted feature decreases in red-shift upon increasing cluster size, from 3240 cm^−1^ for *n* = 6−10, before red-shifting to 3220 cm^−1^ for *n* = 16 and 20. The shoulder feature at 3270 cm^−1^ for *n* = 5 also decreases in red-shift upon addition of water molecules from 3320 cm^−1^ in *n* = 6 to the broad, unresolved feature centered at 3470 cm^−1^ for *n* = 20.

Similar to the monatomic zinc water complexes [70], all cluster sizes present a band between the symmetric and asymmetric O–H stretching regions, starting as a weak feature at 3710 cm^−1^ for *n* = 1, a shoulder at 3680 cm^−1^ for *n* = 2, increasing in intensity to a band at 3680 cm^−1^ for *n* = 3, and staying within 3690–3710 cm^−1^ for *n* = 4–20. Table 1 presents the experimental IRMPD bands for all cluster sizes, with most bands red-shifted from the symmetric and asymmetric O–H bands of isolated water [74].

### 2.1. Zn_2_^+^(H_2_O)_1–6_

**Figure 2:**
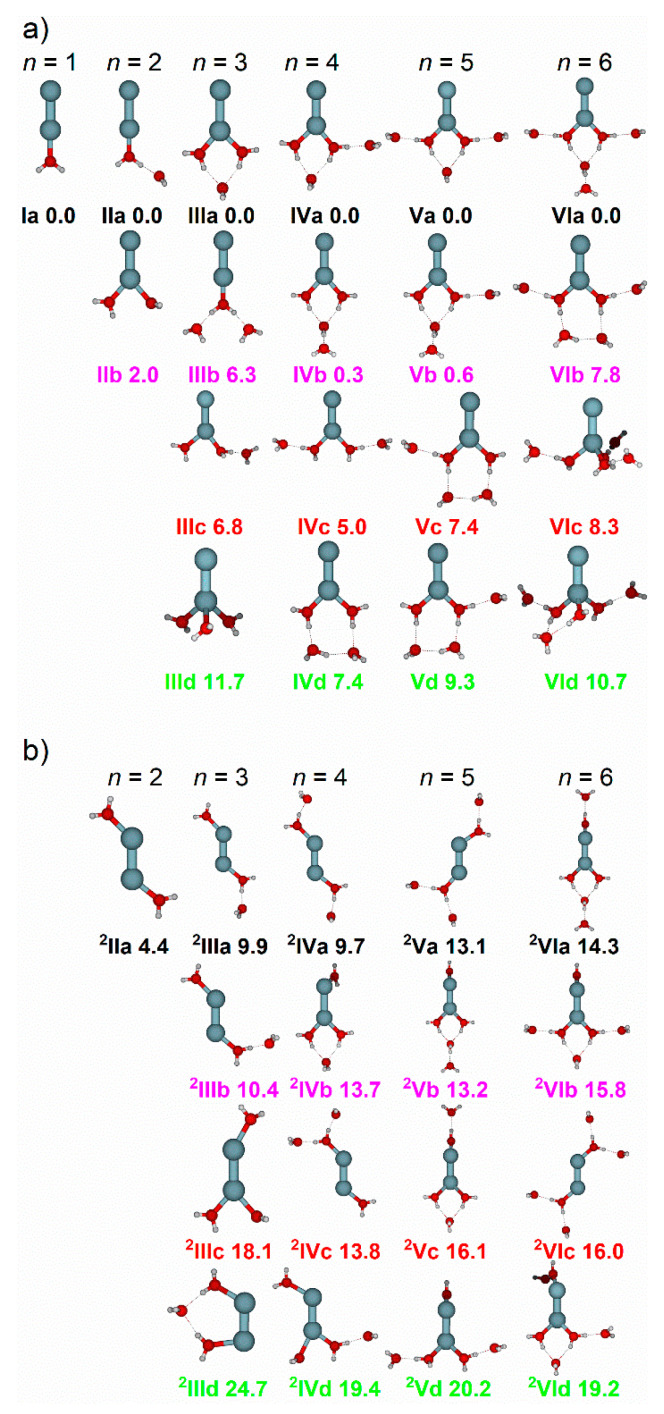
(**a**) Low-lying isomers of Zn_2_^+^(H_2_O)*_n_* clusters (*n* = 1–6) with water molecules coordinated to one Zn atom, and (**b**) isomers of Zn_2_^+^(H_2_O)*_n_* clusters (*n* = 1–6) with water molecules coordinated to both Zn atoms. All isomers were calculated at the B3LYP/aug-cc-pVDZ level of theory, with relative energy given in kJ mol^−1^ inclusive of zero-point energy.

To support the IRMPD spectra, density functional theory calculations were performed in search of low-lying isomers for cluster sizes *n* = 1–6. For all cluster sizes, isomers were selected where water molecules are coordinated to either (a) one Zn atom or (b) both Zn atoms, as shown in Figure 2. For clarity, Roman Numerals are used to denote the cluster size, letters the energetic ordering, and a number to denote the number of zinc atoms coordinated. Using *n* = 2 as an example, **IIa** denotes the lowest-lying isomer with water molecules coordinated to one Zn atom, and **^2^IIa** to both Zn atoms. Throughout the discussion, water binding motifs are also labelled. Water molecules which are directly bound to Zn are denoted core water molecules. Outer water molecules can bind to core water molecules via single, or double hydrogen bonds, denoted single acceptor (SA) and double acceptor (DA) motifs, respectively. Other binding motifs (shown schematically in Supplementary Materials Schemes S1–S3) are also outlined, including S*A, whereby the core water molecule is bound to a single outer water molecule, but also involved in a double-acceptor configuration. Moreover, oDA denotes the “outer” double-acceptor motif, whereby the outer water molecule in a DA motif is bound to another outer water. The “outer” single-acceptor motif (oSA) denotes the O–H stretch in the outer water, and D*A an outer water bound to oSA and a core water. Water binding motifs have been outlined previously [70]. Each binding motif absorbs at a characteristic frequency, as shown by Johnson and co-workers [75]. 

#### 2.1.1. H_2_O Coordinated to One Zn Atom

**Figure 3:**
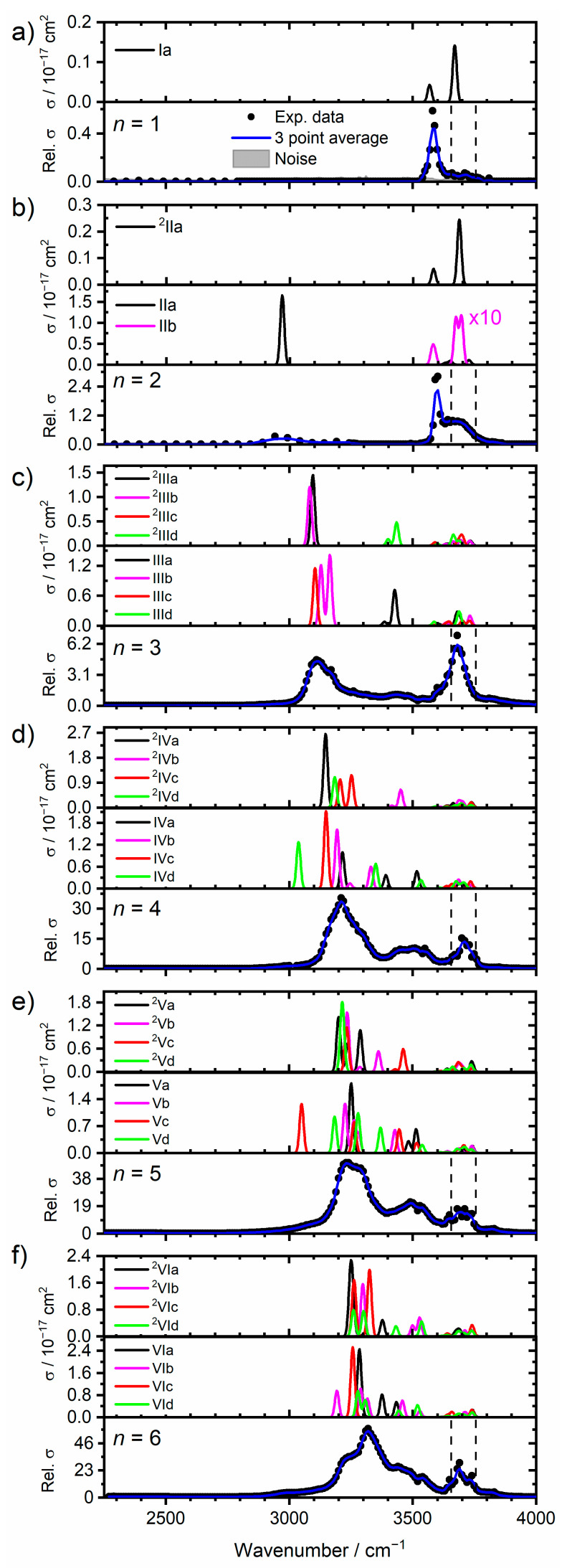
Experimental relative IRMPD photodissociation cross-section (arbitrary units) along with simulated cross-section *σ* of low-lying isomers for (**a**–**f**) Zn_2_^+^(H_2_O)_1–6_, representing the singly coordinated, and doubly coordinated isomers, respectively, at the B3LYP/aug-cc-pVDZ level. In each case photodissociation cross-sections present loss of intact water molecules. The dashed lines in each experimental spectrum correspond to the wavenumbers of the symmetric and asymmetric stretching modes of isolated water at 3657 and 3756 cm^−1^, respectively [74].

Figure 3a–f presents the experimental photodissociation spectra of cluster sizes *n* = 1–6, along with the simulated spectra of low-lying isomers, with water molecules bound to one Zn atom in the Zn_2_^+^ dimer. For every cluster size, *n* = 1–6, (with the exception of **IIId**) the lowest-lying isomers have water molecules coordinated to one zinc atom. In this sense, one could expect that the water molecules will solvate one zinc atom in a similar fashion to the monatomic complexes, Zn^+^(H_2_O)*_n_* [70]. However, taking *n* = 3 as an example, the addition of the Zn atom to Zn^+^(H_2_O)_3_ (forming Zn_2_^+^(H_2_O)_3_) changes the energetic ordering of isomers, shown in Figure 2a. In the case of Zn^+^(H_2_O)_3_, all three water molecules are directly bound to the central Zn atom, representing the lowest energy structure found. For Zn_2_^+^(H_2_O)_3_, this analogue (isomer **IIId**, Figure 2a) lies 11.7 kJ mol^−1^ above the putative global minimum, which presents a coordination number of two (isomer **IIIa**). Interestingly, isomer **IIIb**, presenting one core and two outer water molecules, is the second lowest lying isomer (+6.3 kJ mol^−1^). In contrast, this analogue in Zn^+^(H_2_O)_3_ was calculated to lie 22.3 kJ mol^−1^ above the global minimum. These energetic differences between monatomic and dimer water complexes are observed in sizes *n* = 2–6, and are reflected in the observed IRMPD spectra. For the low-lying calculated isomers *n* = 4–6 in Figure 2a, the range of energies is much greater when compared to their monatomic counterparts. This signifies that the addition of the second zinc atom not only changes the energetic ordering of the isomers, but also increases the energy differences between them. 

For *n* = 1 (Figure 3a) only one isomer is considered, where the water molecule is bound to one Zn atom, adopting a C_2*v*_ configuration (Figure 2a, isomer **Ia**). The simulated spectrum of **Ia** presents two bands: one weak centered at 3568 cm^−1^, and one strong centered at 3670 cm^−1^, representing the symmetric and asymmetric stretches of water, respectively. These bands agree with the experimental band at 3580 cm^−1^, along with the weak shoulder at 3710 cm^−1^. However, the IRMPD band at 3580 cm^−1^ is strong and sharp, whereas the band at 3710 cm^−1^ is weak and broad. This is most likely an IRMPD effect, since two photons are typically required to dissociate Zn^+^(H_2_O), with a binding energy of 85 kJ mol^−1^. In this small system, intramolecular vibrational energy redistribution (IVR) is difficult. We may speculate that IVR works well for the symmetric band, so that both the first and second photon are absorbed in the vibrational ground state of the band, while IVR is accidentally hindered due to the sparse density of states for the asymmetric stretch. In this case, either the band position shifts due to anharmonicity from the fundamental to the hot band excitation, or the system cools by IR emission. Both effects can explain the experimental spectrum. 

In the case of *n* = 2, two isomers are considered, **IIa** and **IIb**. Different to the case of the zinc monomer reported previously, [70] for the zinc dimer, the putative global minimum structure shows an outer water molecule adopting a single-acceptor (SA) configuration, binding to the core water molecule via a single hydrogen bond. This is also reflected in the IRMPD spectrum, Figure 3b, showing a weak, highly red-shifted feature centered at 2965 cm^−1^, the signature of the SA motif in isomer **IIa**. No such red-shifted bands were observed in the monomer analogue, Zn^+^(H_2_O)_2_ [70]. It should be noted that isomers of *n* = 2 calculated at the CCSD/aug-cc-pVDZ//B3LYP/aug-cc-pVDZ level show that the 2-coordinate structure (**IIb**) is the lower-lying isomer by 6.5 kJ mol^−1^. Two more weaker bands are presented for isomer **IIa** at 3651 and 3726 cm^−1^, assigned to the localized core “free” O–H stretch and asymmetric outer O–H stretch, respectively. These bands are in reasonable agreement with the broad feature centered at 3680 cm^−1^. Isomer **IIb** presents one band at 3580 cm^−1^, along with two stronger bands at 3673 and 3695 cm^−1^, due to the symmetric O–H stretch and localized asymmetric O–H stretches of the two core water molecules, respectively. Similar to Zn^+^(H_2_O)_2_, the observed spectrum shows more intense symmetric O–H bands relative to the asymmetric O–H bands, along with increased broadening of the asymmetric bands. As described before, [70] we attribute these observations to the IRMPD process, whereby the first absorbed photon heats the cluster, exciting internal rotations of the water molecules and, potentially, the O–Zn–O bending mode. This assumes that the asymmetric stretch is sensitive to the relative positions of the water molecules, explaining the reduced intensity and band broadening. However, the symmetric stretch remains sharp, implying this band is less sensitive to this effect. Thus, with these observed bands, along with the red-shifted feature at 2965 cm^−1^, the IRMPD spectrum is composed of contributions from both isomers, **IIa** and **IIb**.

Figure 3c shows the IRMPD spectrum of *n* = 3, along with the simulated spectra of the four lowest lying isomers, with water coordinated to one Zn atom, **IIIa**–**IIId**. The intense band at 3110 cm^−1^ is in good agreement with the simulated bands at 3105 cm^−1^ (**IIIc**; isolated O–H stretch of the core water) and 3129 cm^−1^ (**IIIb**; symmetric O–H stretch of the core water) assigned to the SA motif. This observed band shows a shoulder at 3160 cm^−1^, presenting excellent agreement with the asymmetric O–H stretch in the core water in isomer **IIIb** at 3164 cm^−1^. Isomer **IIIa** presents an intense band at 3427 cm^−1^, a weaker band at 3387 cm^−1^ assigned as the in-phase, and out-of-phase core water molecules in the DA motif, respectively. These two bands are in good agreement with the broad experimental feature, displaying a maximum centered at 3445 cm^−1^. Spectral signatures of DA motifs were unobserved in Zn^+^(H_2_O)_3_. The strong band at 3680 cm^−1^ is in agreement with the simulated bands at 3680 cm^−1^ (**IIIa**; O–H core water), 3686 cm^−1^ (**IIId**; O–H core water), 3646 cm^−1^ (**IIIc**; isolated O–H stretch of core water within the SA motif), 3701 cm^−1^ (**IIIc**; asymmetric stretch of isolated core water), and 3730 cm^−1^ (**IIIc**; asymmetric stretch of outer water). Given that isomer **IIId** is calculated to lie 11.7 kJ mol^−1^ above **IIIa**, it is more likely that isomers **IIIa**–**IIIc** are higher in abundance and contribute more to the overall IRMPD spectrum.

The IRMPD spectrum for *n* = 4, along with the simulated spectra of low-lying isomers **IVa**–**IVd**, are shown in Figure 3d. Evidence of more SA and DA signatures are observed, reflected as band broadening of the former along with an increase in relative intensity for the latter. For *n* ≥ 4, it is more informative to discuss the spectra in terms of binding motifs, rather than individual isomers. Water binding motifs are outlined in the supplementary material. The strong, broad band at 3210 cm^−1^ is in good agreement with SA and an outer water bound to a double acceptor site (oDA) binding motifs in isomers **IVa**–**IVc**.

The very broad feature centered at 3490 cm^−1^ matches the absorption of the DA binding motif in isomer **IVa**. The broad band at 3710 cm^−1^ presents satisfactory agreement with the simulated bands from isolated core, and outer waters in oDA, DA and S*A motifs present in isomers **IVa**–**IVc**. As observed, the simulated spectra in this wavelength region, between the symmetric and asymmetric stretches of isolated water (3657 and 3756 cm^−1^), become spectrally congested. Thus, this region is less diagnostic when assigning particular isomers and water binding motifs. Based on the red-shifted bands below 3650 cm^−1^, i.e., the spectral signatures of DA and SA motifs, the observed spectrum is composed of contributions from isomers **IVa**–**IVc**. 

Figure 3e shows the IRMPD spectrum of *n* = 5, along with the simulated spectra of low-lying isomers **Va**–**Vd**. Similar to the IRMPD spectrum of *n* = 4, the observed spectrum of *n* = 5 shows an intense red-shifted broad band centered at 3230 cm^−1^, along with an intense shoulder at 3270 cm^−1^. The intense feature is in good agreement with SA motifs in **Va** and **Vb**, and with the oDA motif in **Vb**. A DA/oDA combination band in **Vb** agrees with the shoulder at 3270 cm^−1^. The S*A motif in **Vc** is also in good agreement, however the red-shifted band at 3050 cm^−1^ (core oSA) presents less clear agreement. Another band at 3445 cm^−1^ (O–H stretch of outer water in oSA) in **Vc** shows some agreement, and could contribute to the overall broad region between the observed bands at 3230 and 3490 cm^−1^. The SA signature at 3278 cm^−1^ in isomer **Vd** is also in good agreement with the observed band at 3270 cm^−1^; however, the simulated band at 3183 cm^−1^ (core oSA) presents less agreement. Signatures of DA motifs in **Va** also fit with the observed bands at 3490 and 3540 cm^−1^. Outer single acceptor (oSA) motifs in **Vc** and **Vd** present agreement with the band at 3540 cm^−1^. All four isomers **Va**–**Vd** present bands within the observed features between 3230 and 3540 cm^−1^, assigned to SA, DA, oDA, and oSA binding motifs. The observed bands show an onset starting at ca. 3010 cm^−1^, which could be due to the most red-shifted band at 3050 cm^−1^ in isomer **Vc** (core O–H bound to the oSA motif). Isomer **Vc** is calculated to lie 7.4 kJ mol^−1^ above isomer **Va**, so will give a minor contribution to the observed spectrum, supporting the presence of the weak onset at ca. 3010 cm^−1^. Thus, the observed IRMPD spectrum mostly consists of isomers **Va** and **Vb**, with minor contributions from isomers **Vc** and **Vd**, along with other low-lying isomers we have not considered.

Figure 3f presents the observed spectrum for *n* = 6, along with simulated spectra of low-lying isomers **VIa**–**VId**. The observed spectrum shows an intense band at 3320 cm^−1^, with a shoulder to the red at 3240 cm^−1^. Based on spectral assignments of Zn^+^(H_2_O)*_n_*, *n* = 2–6, [70] along with *n* = 2–5 in this study, this would imply a lower abundance of isomers with SA waters (most red-shifted), and a higher abundance of isomers with S*A waters (second most red-shifted). This assignment is supported when considering the strong out-of-phase S*A signature in **VIa** at 3283 cm^−1^. Another strong band at 3285 cm^−1^, the oDA signature, is also present in isomer **VIa**. DA signatures in **VIa** are in reasonable agreement with the observed broad feature between 3320 and 3445 cm^−1^. The most red-shifted band at 3193 cm^−1^ (core water bound to outer water in oSA) in **VIb** is in good agreement with the red-shifted shoulder at 3240 cm^−1^. Isomer **VIb** also presents bands at 3289 and 3317 cm^−1^ (core O–H in two SA motifs, respectively) which are in good agreement with the observed intense feature at 3320 cm^−1^, and two more bands at 3458 cm^−1^ (core bound to outer water in D*A) and 3529 cm^−1^ (oSA) which present good agreement with the broad features between 3445 and 3540 cm^−1^. The observed shoulder at 3240 cm^−1^ is thus assigned to the core O–H bound to oSA in isomer **VIb**, in addition to the same binding motif in isomers we have not considered. Given that isomer **VIb** is still higher-lying, calculated as 7.8 kJ mol^−1^ above **VIa**, this isomer offers a minor contribution, reflected as an observed weaker shoulder band. Isomers **VIc** and **VId** are calculated to lie above **VIa** at 8.3 and 10.4 kJ mol^−1^, respectively, so are predicted to play a minor role.

In each cluster size *n* = 4–6, the bands in the region between 3657 and 3756 cm^−1^ are due to the asymmetric O–H stretches of different water binding motifs (core, DA, oDA, SA, S*A, and oSA) giving a crowded and relatively less intense feature, thus this spectral range is less diagnostic when assigning the different binding motifs to the observed IRMPD spectrum.

#### 2.1.2. H_2_O Coordinated to Both Zn Atoms

Figure 3b–f presents the experimental IRMPD spectra of cluster sizes *n* = 2–6 along with the simulated spectra of isomers with water molecules coordinated to both Zn atoms. The structures of these isomers are presented in Figure 2b. Beginning with *n* = 2, Figure 3b shows the observed IRMPD spectrum, along with the simulated spectrum of isomer **^2^IIa**, presenting two bands at 3583 and 3687 cm^−1^, due to the symmetric and asymmetric O–H stretching bands, respectively. Both types of stretch are localized to one water, resulting in two pairs of bands, with each pair coalescing forming a band (two in total). These simulated bands are in good agreement with the experimental bands at 3590 and 3680 cm^−1^. Similar to the spectrum of **IIb**, the asymmetric bands are more intense than the symmetric, with the opposite shown in the experimental spectrum. The experimental IRMPD spectrum of *n* = 2 was assigned to contributions from isomers **IIa** and **IIb**. However, given that the spectrum for **^2^IIa** is in good agreement with the observed spectrum, and is calculated to lie only 4.4 kJ mol^−1^ above **IIa**, we cannot rule out contributions from **^2^IIa**. 

Figure 3c shows the observed spectrum of *n* = 3, along with simulated spectra from isomers **^2^IIIa**–**^2^IIId**. Simulated bands at 3095 cm^−1^ (**^2^IIIa**, black) and 3083 cm^−1^ (**^2^IIIb**, magenta) agree well with the experimental band at 3110 cm^−1^, assigned as core O–H stretches in SA motifs. However, none of these simulated spectra can reproduce the observed feature at 3160 cm^−1^. Isomers **^2^IIIa** and **^2^IIIb** however, are in good agreement with the SA signature at 3110 cm^−1^, and are calculated to lie 9.9 and 10.4 kJ mol^−1^ above **IIIa**, respectively. Thus, isomers **^2^IIIa** and **^2^IIIb** could offer a minor contribution to the IRMPD spectrum, with major contributions from isomers **IIIa**–**IIIc**.

The IRMPD spectrum, along with the simulated spectra of **^2^IVa**–**^2^IVd**, is shown for *n* = 4 in Figure 3d. Isomer **^2^IVa** presents a red-shifted band at 3147 cm^−1^, assigned as the two equivalent SA motifs, and agrees well with the observed band at 3210 cm^−1^. Simulated bands at 3206 and 3252 cm^−1^ in **^2^IVc**, the in-phase and out-of-phase core DA O–H stretches, respectively, present good agreement with the broad feature at 3210 cm^−1^. The simulated band at 3184 cm^−1^ in isomer **^2^IVd**, the SA motif, is also in agreement with this observed band. Evidence of the DA signature is also presented in the simulated spectrum for **^2^IVb** at 3452 cm^−1^. Though these simulated bands present agreement with the observed spectrum, isomers **^2^IVa**–**^2^IVd** lie ≥ 9.7 kJ mol^−1^ above **IVa**, so are expected to have a minor contribution to the IRMPD spectrum. 

Upon addition of another water molecule, the IRMPD spectrum for *n* = 5, along with the simulated spectra of **^2^Va**–**^2^Vd**, is presented in Figure 3e. As observed in the simulated isomers, Figure 2b, the difference between the global minimum and the lowest-lying doubly coordinated isomer increases at five water molecules (from 9.7 kJ mol^−1^ in **IVa** and **^2^IVa** to 13.1 kJ mol^−1^ in **Va** and **^2^Va**). Thus, given the large energy differences between isomers **Va**–**Vd** and **^2^Va**–**^2^Vd**, the observed IRMPD spectrum largely consists of singly coordinated zinc isomers, namely **Va** and **Vb**, with minor contributions from other singly coordinated, as well as doubly coordinated, isomers.

The IRMPD spectrum of *n* = 6, along with the simulated spectra of **^2^VIa**–**^2^VId**, is presented in Figure 3f. Similar to *n* = 5, the doubly coordinated isomers (Figure 2b) of *n* = 6 are calculated to lie significantly higher than the singly coordinated isomers, at ≥14.3 kJ mol^−1^ above **VIa**. Indeed, upon increasing cluster size, the energy difference between singly and doubly coordinated isomers remains high (see Supplementary Materials Figure S8). Thus, the doubly coordinated isomers are expected to be lower in abundance and will offer a very minor contribution to the observed IRMPD spectrum for *n* > 5.

#### 2.1.3. Zn Dissociation

**Figure 4:**
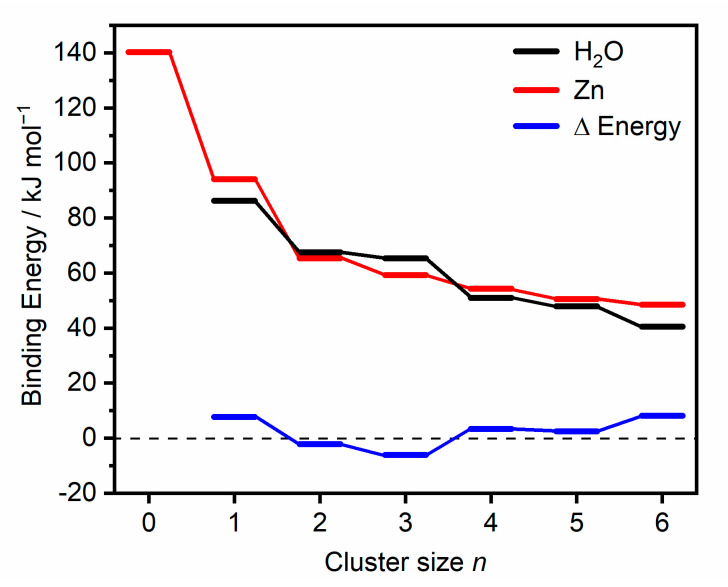
Calculated binding energies of one water molecule (black), one Zn atom (red), and the difference in these binding energies (blue) in lowest-lying Zn_2_^+^(H_2_O)*_n_* clusters (*n* = 1–6) calculated at the CCSD/aug-cc-pVDZ//B3LYP/aug-cc-pDVZ level of theory. These isomers correspond to water bound to one Zn atom. Using the water molecule as an example, binding energies are calculated: *E*[Zn_2_^+^(H_2_O)*_n_*] − *E*[Zn_2_^+^(H_2_O)*_n_*_−1_] − *E*[(H_2_O)], including zero-point correction.

In addition to dissociation of water molecules, we also observe the loss of a neutral zinc atom for Zn_2_^+^(H_2_O)_3_ (Figure 1). This channel seems to be independent of the IR wavelength, presenting a qualitatively similar spectrum to that of water loss, only with a lower relative intensity. The main factor proposed for this observation arises when considering energetic contributions. Figure 4 presents the calculated binding energies of one neutral zinc atom and one water molecule, along with the energy difference as function of cluster size for the lowest-lying isomer in each case, calculated at the CCSD/aug-cc-pVDZ//B3LYP/aug-cc-pVDZ level. Calculated binding energies of Zn and water at the B3LYP/aug-cc-pVDZ level of theory were also carried out for comparison and are presented in the supplementary material (Supplementary Materials Figure S1). Beginning with *n* = 0 (i.e., Zn_2_^+^), Zn dissociation is calculated as 141 kJ mol^−1^ (1.46 eV), lower than the previous calculations from Gutsev [7] (1.73 eV) but higher than the experimental value of 0.56 eV by Buckner. [4] The experimental value, however, was estimated from the bond dissociation energy of the neutral dimer, which in turn was estimated from Zn vapor viscosity data, [76] and the ionization potentials of Zn and Zn_2_, the latter determined by gas-phase charge transfer bracketing experiments [4]. Buckner et al. also estimated the bond length of Zn_2_^+^, assuming solely ion-induced dipole bonding interactions, giving an internuclear distance of 3.00 Å, which is much higher than the Zn–Zn distance calculated by Gutsev [7] (2.60 Å) and in our calculations, 2.62 Å. The respective dissociation energy and bond length at the CCSD/aug-cc-pVDZ level, i.e., after CCSD optimization, amount to 1.46 eV and 2.62 Å, respectively. The binding in Zn_2_^+^ is most likely due to covalent bonding, primarily with 4sσ bonding character. This would imply minor contributions from 3d orbitals, yielding a bond order of 0.5 and an equal charge distribution on both Zn atoms. Similar bonding mechanisms have been proposed in collision-induced dissociation measurements of Mn_2_^+^, [77] in addition to photoelectron spectra of Cu_2_^–^, [78] which is isoelectronic to Zn_2_^+^. For almost all cluster sizes the binding energy of zinc is higher than the binding energy of a water molecule, and for both Zn dissociation and water dissociation, the binding energy (BE) decreases with increasing cluster size. Blue bars in Figure 4 show the binding energy differences (BE(Zn)—BE(H_2_O)), which for cluster sizes *n* = 1 and 4–6 present a positive energy difference. Given these positive binding energy differences, we expect water dissociation dominates, which is concordant with our IRMPD spectra of *n* = 1 and 4–6. For cluster sizes *n* = 2 and 3, the binding energy of neutral Zn is lower than that of water, presenting negative energy differences (−2.1 and −6.0 kJ mol^−1^, respectively) in Figure 4. Subtle energy differences between the CCSD (Figure 4) and B3LYP (Supplementary Materials Figure S1) calculated binding energies are observed. Only CCSD calculations yield negative binding energy differences for *n* = 2 and 3. Given that we observe only water dissociation in the IRMPD spectrum of Zn_2_^+^(H_2_O)_2_, we believe that the binding energy difference is not sufficiently large in order for the Zn dissociation channel to be observed experimentally. The small binding energy difference of −2.1 kJ mol^−1^ in *n* = 2 is also within error of our calculations. For *n* = 3, however, the dominant channel is water dissociation, with Zn dissociation presenting a weaker, but prominent, spectrum. We attribute the appearance of the Zn channel to the binding energy difference of −6.0 kJ mol^−1^, which, although a weaker channel, is enough to be observed in the IRMPD spectrum. In larger clusters, *n* > 3, further factors suppressing the Zn dissociation channel could be attributed to the decrease in water binding energies, resulting in the dissociation of weakly bound outer shell water molecules, preferred over Zn dissociation.

### 2.2. Zn_2_^+^(H_2_O)_8_ and Zn_2_^+^(H_2_O)_10_

**Figure 5:**
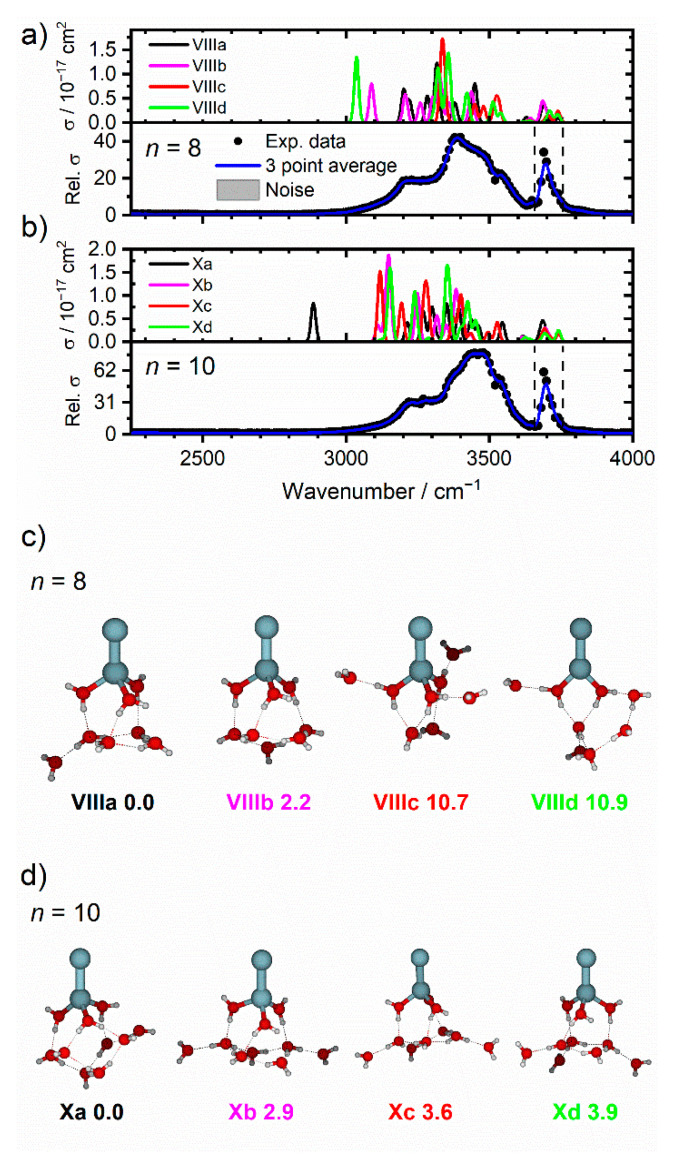
Experimental relative IRMPD photodissociation cross-section (arbitrary units) along with simulated cross-section *σ* of low-lying isomers for (**a**) Zn_2_^+^(H_2_O)_8_ and (**b**) Zn_2_^+^(H_2_O)_10_. The dashed lines in each experimental spectrum correspond to the wavenumbers of the symmetric and asymmetric stretching modes of isolated water at 3657 and 3756 cm^−1^, respectively [74]. Low-lying isomers of *n* = 8 and *n* = 10, with water coordinated to one Zn atom, are shown in (**c**,**d**), respectively. All isomers were calculated at the B3LYP/aug-cc-pVDZ level of theory, with relative energy given in kJ mol^−1^ inclusive of zero-point energy.

Figure 5 presents the IRMPD spectra of cluster sizes *n* = 8 and 10, along with simulated spectra of low-lying isomers, Figure 5a,b, respectively. Figure 5c,d also shows the structures of low-lying isomers of *n* = 8 and 10. Similar to the smaller clusters, the lowest-lying isomers are those with the water molecules coordinated to one Zn atom. In contrast to smaller clusters, however, is the coordination number, which has increased from two to three at *n* = 8. This would suggest that for smaller clusters, the hydrogen bonding between water molecules is favored over three-coordinate structures, while for *n* ≥ 8, enough water molecules are available to form a compact hydrogen bonded network with three molecules coordinating to a Zn atom, without imposing too much strain on the geometry of three coordinate complex as well as the hydrogen bonds. The IRMPD spectrum for *n* = 8 (Figure 5a) presents a broad feature between 3050 and 3640 cm^−1^, with a pronounced maximum at 3390 cm^−1^, along with a shoulder feature to the red at 3220 cm^−1^ and two shoulders to the blue at 3460 and 3540 cm^−1^. A sharper band, assigned to the asymmetric free O–H stretches, is shown at 3670 cm^−1^. These features are in agreement with the simulated spectra of isomer **VIIIa**, especially the intense feature at 3316 cm^−1^. Given the broad, unresolved features in the observed spectrum, simulated spectra of all four isomers, **VIIIa**–**VIIId**, show agreement with the IRMPD spectrum. However, given the calculated energies of isomers **VIIIc** and **VIIId** exceed 10.7 kJ mol^−1^, these isomers are likely to only give a minor contribution to the overall spectrum. The spectral congestion and overlap of bands in the two lowest-lying isomers **VIIIa** and **VIIIb** contributes to the broad unresolved features in the observed spectrum. As the number of water molecules increases, the potential energy landscape becomes ever-increasingly complicated, whereby water ligands can change position across isomeric structures separated by shallow energetic barriers. Thus, for larger sizes these clusters represent fluxional systems exploring the potential energy landscape, whereby the spectra represent structural binding motifs, rather than individual isomers. Observed in monatomic Zn^+^(H_2_O)*_n_* clusters [70], the retention of a coordination number of three was caused by the *n*s^1^ valence electron configuration, which caused incoming ligands to experience repulsion from the occupied s orbital. Thus, water molecules bind to the same side of the metal center, even at larger cluster sizes. A similar effect is observed in Zn_2_^+^(H_2_O)*_n_* clusters, whereby incoming water molecules bind to one Zn atom and avoid binding to the second Zn atom.

The IRMPD spectrum of *n* = 10 is presented in Figure 5b, which also shows a broad absorption between 3050 and 3645 cm^−1^, with a maximum at 3460 cm^−1^, along with a shoulder to the red at 3280 cm^−1^, and a blue shoulder at 3530 cm^−1^. A sharper band to the blue at 3700 cm^−1^ is also shown. The low-lying isomers of Zn_2_^+^(H_2_O)_10_ are presented in Figure 5d, whereby the difference in energy between each isomer is much lower (all four are within 3.9 kJ mol^−1^) when compared to the smaller clusters. The simulated spectra of all four isomers present many bands, which accounts for the broad absorption in the observed spectrum. The spectrum of **Xa**, however, presents a red-shifted band at 2885 cm^−1^, assigned to the O–H stretch of an outer water hydrogen-bonded to three other outer water molecules, which is not observed experimentally. This red-shifted band was also calculated in the simulated spectrum of the putative global minimum structure for Zn^+^(H_2_O)_10_, and signifies significant weakening of the O–H bond and emergence of the mobile proton. Just as is the case in this study, this red-shifted band was not observed in monatomic zinc-water clusters. Isomers **Xb**–**Xd** do not present such red-shifted bands, and instead show more outer water molecules in SA binding motifs. This would suggest that warmer isomers (i.e., the cluster ensemble allowed to reach thermal equilibrium at 80 K) are present in the ICR cell. This is concordant with our previous study on Zn^+^(H_2_O)*_n_* clusters, whereby entropic effects (higher presence of less rigidly bound SA motifs) dominate enthalpic effects at higher temperatures [70]. Each mass-selected cluster (ensemble of different isomers) is exposed to 80 K black-body infrared radiation in the ICR cell, thus more entropically favored isomers are present and more prominent in the observed spectrum.

The IRMPD spectra of larger clusters *n* = 16 and 20 (Figure 1) look qualitatively similar to the spectrum of *n* = 10, presenting a broad absorption between 2950 and 3650 and between 2920 and 3660 cm^−1^, respectively. The feature at 3220 cm^−1^ is more prominent for *n* = 16 and 20. Given there are no significant spectral changes at these larger cluster sizes, we believe there are no significant structural changes; a coordination of three is most likely retained, with asymmetric solvation at one Zn atom. Similar to Zn^+^(H_2_O)*_n_* [70], there are no highly red-shifted bands, which would signify the emergence of a mobile proton within this size regime.

The infrared signature of the mobile proton; a weak, broad, structureless absorption between 2800 and 3500 cm^−1^ was observed previously in the D_2_ “tagged” vibrational predissociation spectra of M^2+^OH^–^(H_2_O)*_n_*_=1–5_ (M = Mg, Ca) by Johnson and co-workers [79]. This signature, unobserved in Zn^+^(H_2_O)*_n_* [70], is also not present in the IRMPD spectra of Zn_2_^+^(H_2_O)_16,20_; thus, we rule out formation of the Zn–HZnOH^+^ motif in larger clusters. 

## 3. Experimental and Computational Details

All experimental measurements were performed on a modified 4.7 Tesla Bruker/Spectrospin CMS47X FT-ICR mass spectrometer [80,81,82,83,84] equipped with a Bruker infinity cell [85] and laser vaporization source [86,87]. To briefly summarize, a frequency-doubled Litron Nano S 60-30 Nd:YAG laser (532 nm, 5 mJ/pulse, 30 Hz) is focused onto a rotating solid disc of isotopically enriched zinc (^64^Zn 99.4%, STB Isotope Germany, GmbH), producing a plasma. This plasma is then entrained into a pulse of the desired gas mixture (H_2_O in helium) produced via a homebuilt piezoelectric valve. The ensuing pulse is cooled via supersonic jet expansion into the source chamber. The gas pulse traverses through a skimmer (forming the molecular beam), and ions are guided via an electrostatic lens setup into the center of the ICR cell. Ions of the composition Zn_2_^+^(H_2_O)_n_ (*n* = 1–20) are then stored and mass-selected within the 4.7 T magnetic field [88] under ultrahigh vacuum conditions (ca. 5 × 10^−10^ mbar). The ICR cell is surrounded by a copper jacket, whereby the temperature of the cell is controlled and cooled to ca. 80 K via liquid nitrogen [89,90] for all measurements, minimizing the effects of black body infrared radiative dissociation (BIRD) [91,92,93,94,95,96]. 

On the opposite side of the magnet, the output radiation of a tunable IR OPO laser system (EKSPLA NT277, 1000 Hz repetition rate, pulse duration <10 ns) is aligned into the cell through a CaF_2_ window [97]. Absorption of infrared photons, leading to photodissociation events is measured via mass spectrometry [98]. Monitoring the precursor and fragment intensities as a function of wavenumber yields the infrared spectrum of the complex of interest. Infrared spectra were recorded in the 2250–4000 cm^−1^ region, probing the O–H symmetric and asymmetric stretching modes. Specific details on the laser setup can be found in previous publications [97,99]. To account for laser energy and irradiation time, single-photon cross-sections, *σ*, are calculated by using a modified Beer–Lambert equation:

(3) $$I_{0}=\left(\overset{n}{\underset{i=0}{\sum }}I_{i}\right)\exp\left(-\frac{\sigma \lambda Pt}{hcA}-k\right)$$

where *I*_0_ represents the intensity of the precursor ion, *I_i_* the fragment ion intensity, *λ* the laser wavelength, *P* the laser power, *t* the irradiation time, *h* the Planck constant, *A* the area of the laser beam and *k* an empirical factor which corrects for any contributions due to BIRD. 

To complement the experimental findings, the structures, along with their corresponding infrared spectra, of Zn_2_^+^(H_2_O)*_n_* were calculated using density functional theory (DFT). Calculations were performed by using the B3LYP functional coupled with the aug-cc-pVDZ basis set. Simulated infrared spectra were generated by implementing Gaussian functions to the band positions, each with a full width at half maximum (FWHM) of 20 cm^−1^, using a scaling factor of 0.96. All structures considered represent local minima and are in the doublet spin multiplicity corresponding to the ground state. Other multiplicities, such as quartet or sextet, lie considerably higher in energy (487 and 1647 kJ mol^−1^, respectively). Binding energies for the low-lying structures for *n* = 0–6 were calculated through single-point recalculation of B3LYP/aug-cc-pVDZ at the coupled cluster singles and doubles (CCSD) level with the aug-cc-pVDZ basis set. Charges on atoms were calculated by using the CHELPG scheme, [100] with the radius of 1.39 Å chosen for Zn. Calculations were performed using the Gaussian16 software package [101]. 

## 4. Conclusions

Cationic zinc dimer–water complexes (Zn_2_^+^(H_2_O)*_n_*, *n* = 1–20) were formed in the gas-phase by laser vaporization, size-selected, and stored in the center of an ICR cell cooled to ca. 80 K. Infrared multiple photon dissociation spectra were recorded for each cluster size revealing structural information and the nature of water binding sites to Zn_2_^+^. Photodissociation of intact water molecules is the dominant channel for every cluster size, with the exception of *n* = 3, whereby Zn photodissociation is also observed as a weaker channel. 

IRMPD spectra are in agreement with simulated spectra of low-lying isomers, whereby different structural motifs can be assigned to bands based on the relative red-shift in band position. In all sizes, asymmetric solvation to one Zn atom in the dimer present the energetically lower-lying isomers. Based on the relative energy of calculated structural isomers, along with infrared band assignments, asymmetric solvation, whereby water molecules bind exclusively to one Zn atom, dominates the observed spectra even at larger cluster sizes. Spectroscopy experiments in the ultraviolet/visible (UV/VIS) region may help to further support these claims of asymmetric solvation.

Zn photodissociation observed in *n* = 3 is rationalized by an interplay of water and Zn binding energy. Calculated binding energies show subtle differences in water binding energy vs. Zn binding energy for smaller clusters, with a significant decrease in Zn binding at *n* = 3. This anomalous Zn binding energy at *n* = 3 helps to rationalize the observed Zn dissociation in the infrared spectrum.

## Figures and Tables

**Table 1:** Experimentally observed IRMPD O–H band position of Zn_2_^+^(H_2_O)*_n_* complexes (*n* = 1–8, 10, 16, and 20) along with the isomer assignment and water binding motif.

Cluster	Experimental IRMPD Band Position/cm^−1^	Isomer Assignment and Binding Motif
Zn_2_^+^(H_2_O)	3580 3710	**Ia** **Ia**
Zn_2_^+^(H_2_O)_2_	2965 3590 3680	**IIa**-SA **IIa**, **IIb** **IIa**, **IIb**
Zn_2_^+^(H_2_O)_3_	3110 3160 3445 3605 3680	**IIIc**-SA, **IIIb**-SA(sym) **IIIb**-SA(asym) **IIIa**-DA **IIIb** **IIIa**–**IIIc**
Zn_2_^+^(H_2_O)_4_	3210 3290 3490 3710	**IVa**-S*A, **IVb**-oDA, **IVc**-SA **IVb**-DA **IVa**-DA **IVa**–**IVc**
Zn_2_^+^(H_2_O)_5_	3230 3270 3490 3540 3690	**Va**-SA, **Vb**-oDA **Vb**-SA, **Vb**-DA/oDA **Va**-DA **Va**-DA **Va**-**Vb**
Zn_2_^+^(H_2_O)_6_	3240 3320 3445 3540 3690 3730	**VIa**-S*A, **VIa**-oDA, **VIb**-oSA **VIb**-SA **VIa**-DA, **VIb**-D*A **VIb**-oSA **Via**, **VIb** **Via**, **VIb**
Zn_2_^+^(H_2_O)_7_	2985–3620, 3220, 3360, 3460, 3540, 3690, 3730	
Zn_2_^+^(H_2_O)_8_	3050–3640, 3220, 3390, 3460, 3540, 3670	
Zn_2_^+^(H_2_O)_10_	3050–3645, 3220, 3460, 3530, 3700	
Zn_2_^+^(H_2_O)_16_	2950–3650, 3220, 3450, 3540, 3700	
Zn_2_^+^(H_2_O)_20_	2920–3660, 3220, 3460, 3560, 3700	

## Data Availability

The data presented in this study are available in the supplementary material.

## Supplementary Materials

The following are available online at https://www.mdpi.com/article/10.3390/ijms22116026/s1. Figures S1 and S2: Calculated binding energies. Figures S3 and S4: Calculated charges, spin densities, and bond distances of Zn–Zn. Figure S5: IRMPD cross-sections. Schemes S1–S3: Outer water binding motifs. Figures S6–S8: Additional calculated structures and Cartesian coordinates of the optimized structures. IRMPD spectra in ASCII format for *n* = 1–8, 10, 16 and 20.
