# Supplementary material for: Asymmetric Solvation of the Zinc Dimer Cation Revealed by Infrared Multiple Photon Dissociation Spectroscopy of Zn2+(H2O)n (n = 1–20)
Source: Int J Mol Sci. 2021 Jun 2;22(11):6026. doi: 10.3390/ijms22116026 (PMC8199724; doi:10.3390/ijms22116026)
Supplement: Supplementary file 1 [file ijms-22-06026-s001.zip › ijms-1221133-supplementary.pdf]

Supplementary Material

Asymmetric solvation of the zinc dimer cation revealed by infrared multiple photon dissociation spectroscopy of $Zn_2^+(H_2O)_n$ (n = 1–20)

Ethan M. Cunningham *,†, Thomas Taxer †, Jakob Heller, Milan Ončák, Christian van der Linde and Martin K. Beyer *

Institut für Ionenphysik und Angewandte Physik, Universität Innsbruck, Technikerstraße 25, 6020 Innsbruck, Austria

*Correspondence: ethan.cunningham@uibk.ac.at (E.M.C.); martin.beyer@uibk.ac.at (M.K.B.)

†These authors contributed equally to this work.

Table of Contents

Calculated Binding Energies 2

Calculated Charges, Spin Densities, and Bond Distances of Zn–Zn 4

Calculating IRMPD Cross Sections 6

Outer Water Binding Motifs 7

Calculated Structures 9

i) Coordination to one Zn atom in $Zn_2^+(H_2O)_n$ clusters 9

ii) Coordination to both Zn atoms in $Zn_2^+(H_2O)_n$ clusters 11

Infrared Multiple Photon Dissociation Spectra of $Zn_2^+(H_2O)_n$ (n = 1–20) 38

References 80

Calculated Binding Energies

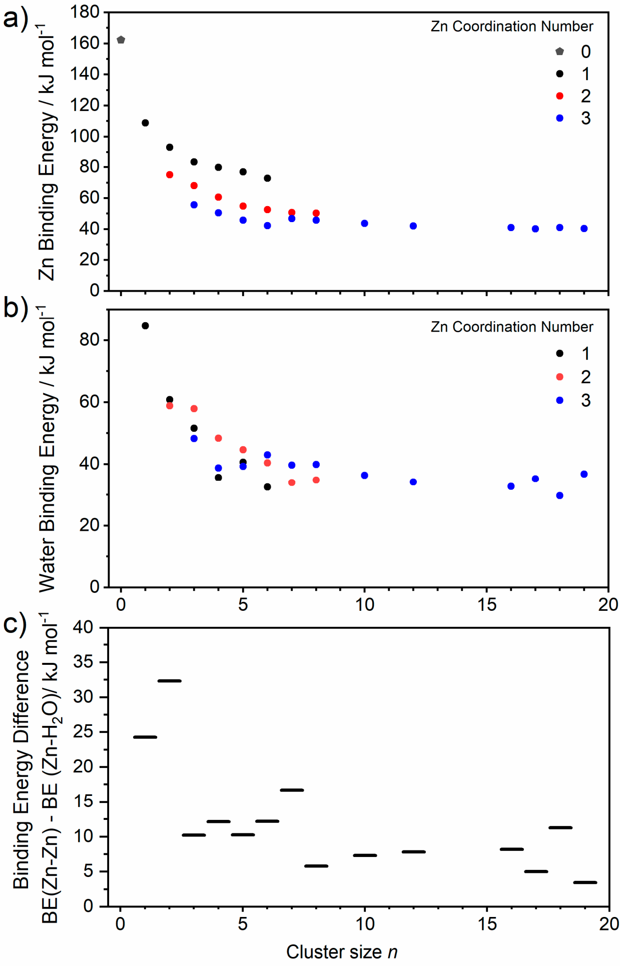

Figure S1. Calculated binding energies of a) one Zn atom, and b) one water molecule in $Zn_2^+(H_2O)_n$ clusters (n = 1–19) calculated at the B3LYP/aug-cc-pVDZ level of theory. These isomers correspond to water bound to one Zn atom. Using the water molecule as an example, binding energies are calculated: $E[Zn_2^+(H_2O)_n] - E[Zn_2^+(H_2O)_{n-1}] - E[(H_2O)]$ including zero-point correction. For larger clusters, in particular n = 10, 12, 16, the binding energies were calculated using multiple water molecules, $(H_2O)_m$, where m = 2, 2, and 4 for n = 10, 12, and 16, respectively. Calculated binding energy differences between lowest energy isomers in panels a) and b) are shown in c).

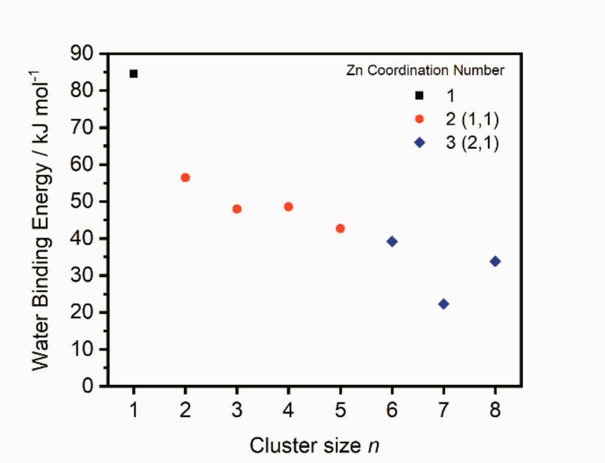

Figure S2. Calculated binding energies of one water molecule in $Zn_2^+(H_2O)_n$ clusters (n = 1–8) calculated at the B3LYP/aug-cc-pVDZ level of theory. These isomers correspond to the lowest energy structure with water molecules bound to both Zn atoms. Binding energies are calculated: $E[Zn_2^+(H_2O)_n]$ – $E[Zn_2^+(H_2O)_{n-1}]$ – $E[(H_2O)]$ including zero-point correction. For a particular structure, the minimum value binding energy is given, where $Zn_2^+(H_2O)_n$ and $Zn_2^+(H_2O)_{n-1}$ have the same substructure, with one water missing. In some cases, the minimum value corresponds to the $Zn_2^+(H_2O)_{n-1}$ structure with water molecules bound to one Zn atom. Numbers in brackets represent the number of water molecules to each Zn atom: taking 3(2,1) as an example, 2 waters are bound to one Zn, with 1 water bound to the other Zn atom.

Calculated Charges, Spin Densities, and Bond Distances of Zn–Zn

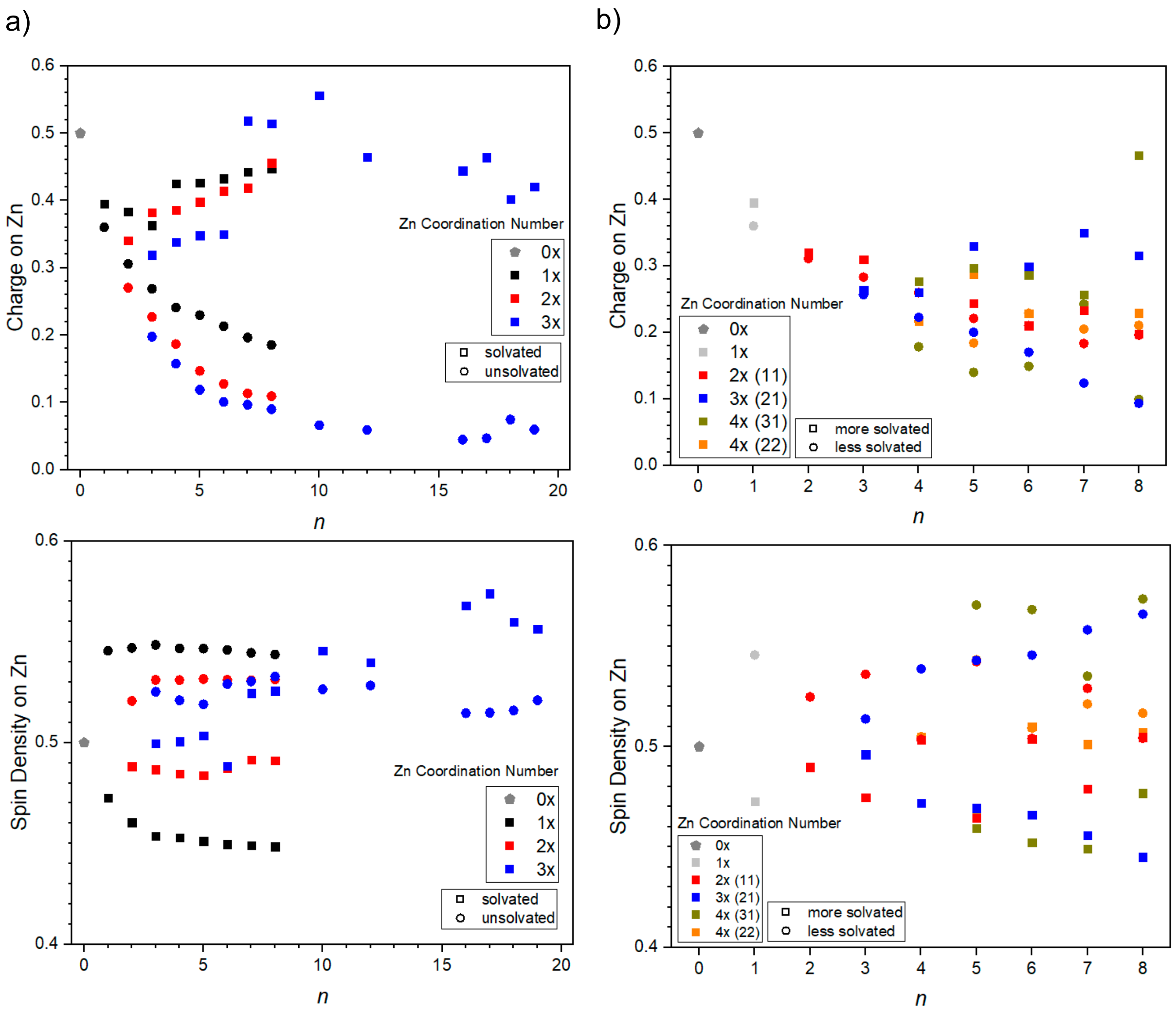

Figure S3. Calculated CHELPG charges and spin densities of $Zn_2^+(H_2O)_n$ clusters for a) water bound to one Zn atom (n = 1–19), and b) water bound to both Zn atoms (n = 1–8) calculated at the B3LYP/aug-cc-pVDZ level of theory. A radius 1.39 Å of was chosen for the Zn atom.

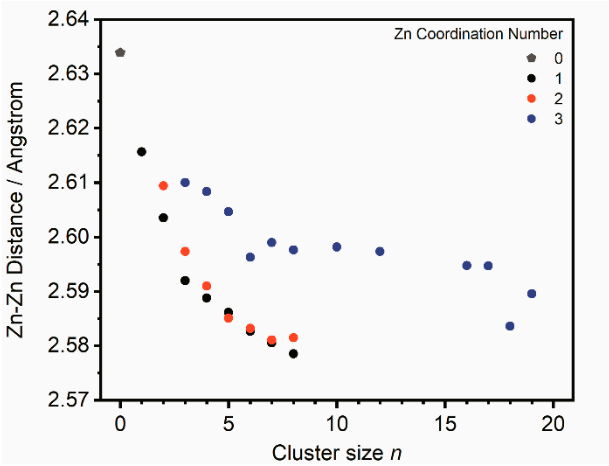

Figure S4. Zn–Zn bond distances obtained from calculated singly-coordinated isomers of $Zn_2^+(H_2O)_n$ clusters (n = 1–19) calculated at the B3LYP/aug-cc-pVDZ level of theory. The singly-coordinated Zn coordination numbers of 1, 2, and 3 are shown in black, red, and blue, respectively.

Calculating IRMPD Cross Sections

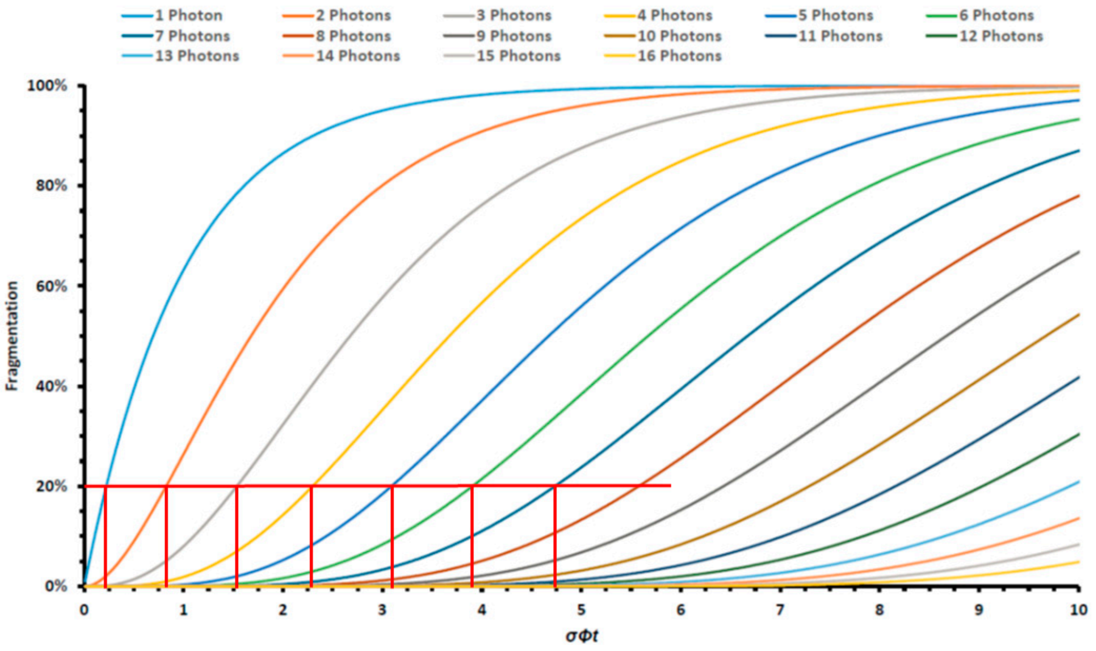

Figure S5. Quantitative analysis of multiphoton absorption, each curve representing *k* photons (*k* = 1–16) with the fragmentation yield (%) and $\sigma\Phi t$. Reproduced with permission from J. Heller, M. Ončák, N. K. Bersenkowitsch, C. van der Linde and M. K. Beyer, *Eur. J. Mass Spectrom.*, 2019, **25**, 122-132.

As outlined in previous publications, [1–3] infrared multi-photon photodissociation cross sections are calculated assuming consecutive first-order reactions, the kinetics of photon absorption are shown in Figure SX. Using Figure SX, along with the fragmentation yield after absorption of the kth photon, the value of $\sigma\Phi t$ can be readily obtained. By way of example, the values of $\sigma\Phi t$ are shown for 1–7 photons, assuming a 20% fragmentation yield. The photon flux, Φ, is measured using a power meter, t is controlled using the LabView software, thus the cross section, σ, can be calculated from $\sigma\Phi t$.

The number of photons required to dissociate a water molecule ranges from one to two, details of which is found in previous publications [1–3]. For all cluster sizes, photodissociation leading to loss of water molecules was found to be the only photofragment channel. To account for laser energy and irradiation time, single-photon cross sections, σ, are calculated using a modified Beer-Lambert equation, details are outlined in the experimental section of the main article.

Outer Water Binding Motifs

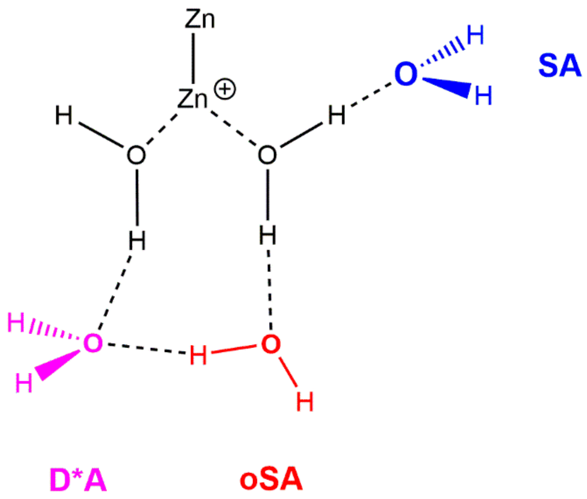

Scheme S1. Schematic representation of the $Zn_2^+(H_2O)_5$ cluster displaying three water configurations; a single-acceptor motif (SA), along with an outer single-acceptor motif (oSA) and an outer water bound to the oSA motif (D*A). ''Core'' water molecules represent those directly bound to the Zn centre.

It should be noted that, in the case of SA, assignments are made to the core O–H in single acceptor (SA) motifs. This is different when assigning bands to the oSA motif, whereby it is the O–H vibration of the second sphere water (red H_2O molecule). oSA and D*A both represent "outer" single- and double-acceptor water molecules in the second hydration sphere (*i.e.* not directly bound to the Zn^+ centre). The O–H bonds in D*A are isolated, whereas oSA has one isolated O–H with the other O–H bond hydrogen-bound to D*A. The D*A water is bound *via* two hydrogen bonds, one from the core water, and another from oSA, thus is denoted a double-acceptor.

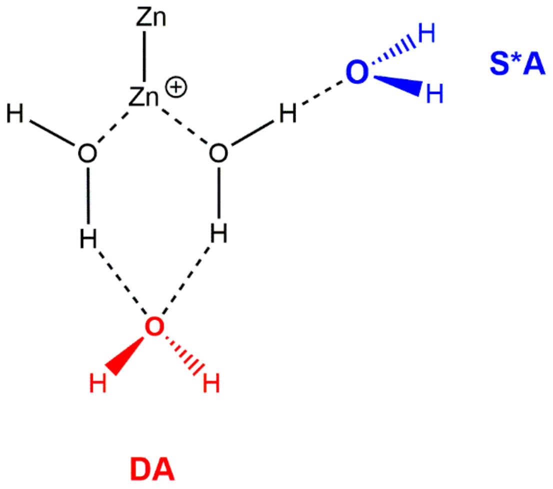

Scheme S2. Schematic representation of the $Zn_2^+(H_2O)_4$ cluster displaying two water configurations; a single-acceptor motif (S*A), along with the double-acceptor motif (DA).

S*A is a different type of single-acceptor configuration, whereby the core water is bound to a single outer water, but also involved in a double-acceptor configuration.

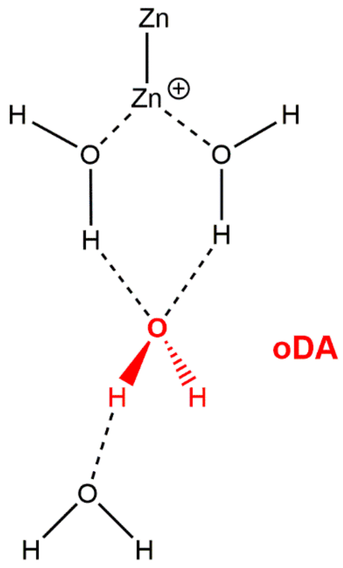

Scheme S3. Schematic representation of the $Zn_2^+(H_2O)_4$ cluster showing the double-acceptor water molecule bound to another outer water molecule (oDA).

Calculated Structures

i) Coordination to one Zn atom in $Zn_2^+(H_2O)_n$ clusters

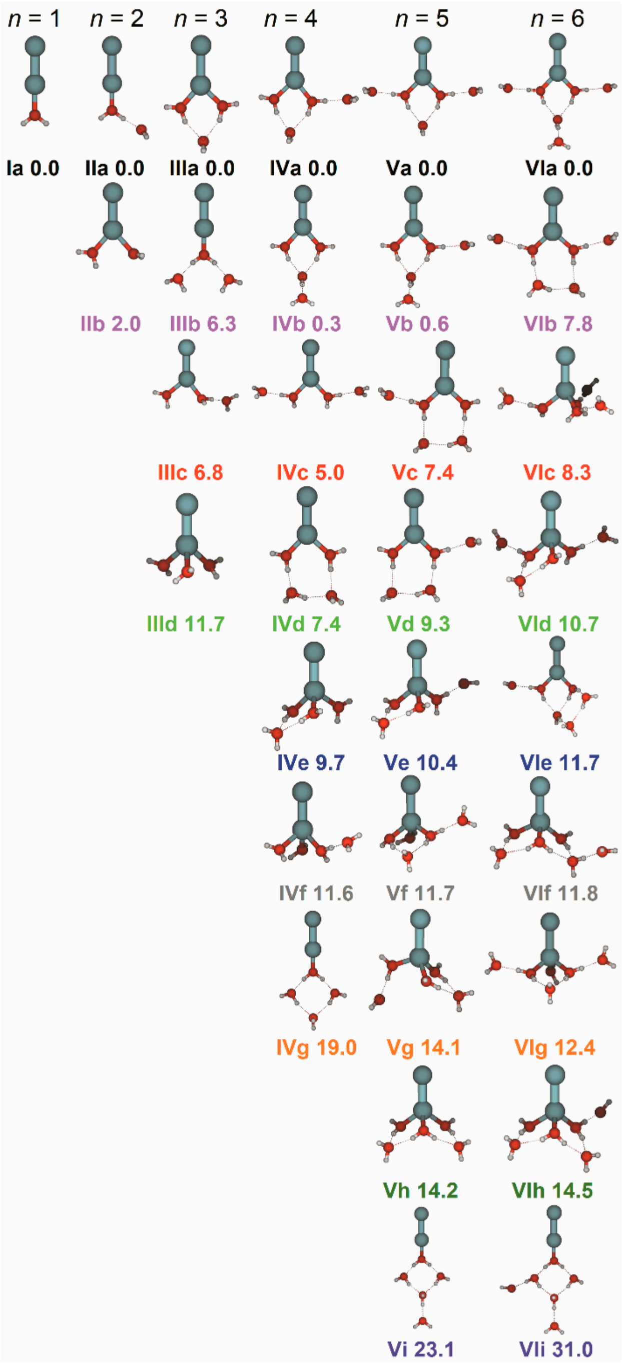

Figure S6. Low-lying isomers of $Zn_2^+(H_2O)_n$ clusters (n = 1–6) with water molecules bound to one Zn atom calculated at the B3LYP/aug-cc-pVDZ level of theory along with relative energy given in kJ mol^{-1} inclusive of zero-point energy.

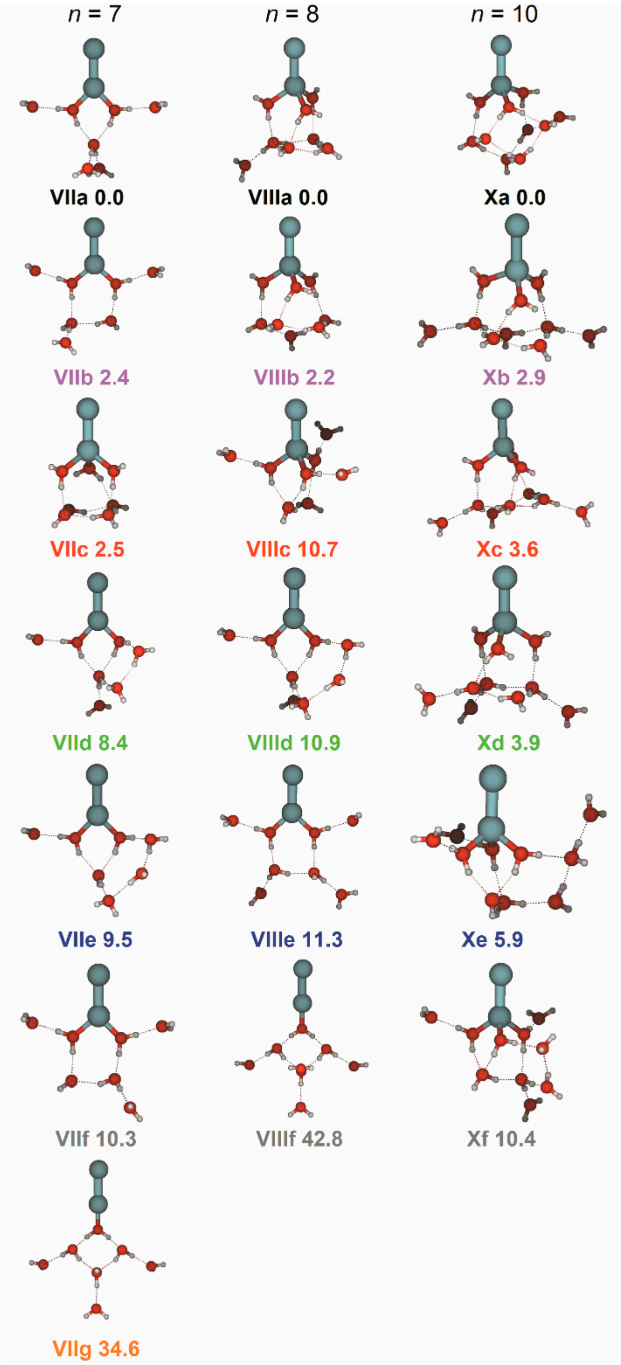

Figure S7. Low-lying isomers of $Zn_2^+(H_2O)_n$ clusters (n = 7–10) with water molecules bound to one Zn atom calculated at the B3LYP/aug-cc-pVDZ level of theory along with relative energy given in kJ mol^{-1} inclusive of zero-point energy.

ii) Coordination to both Zn atoms in $Zn_2^+(H_2O)_n$ clusters

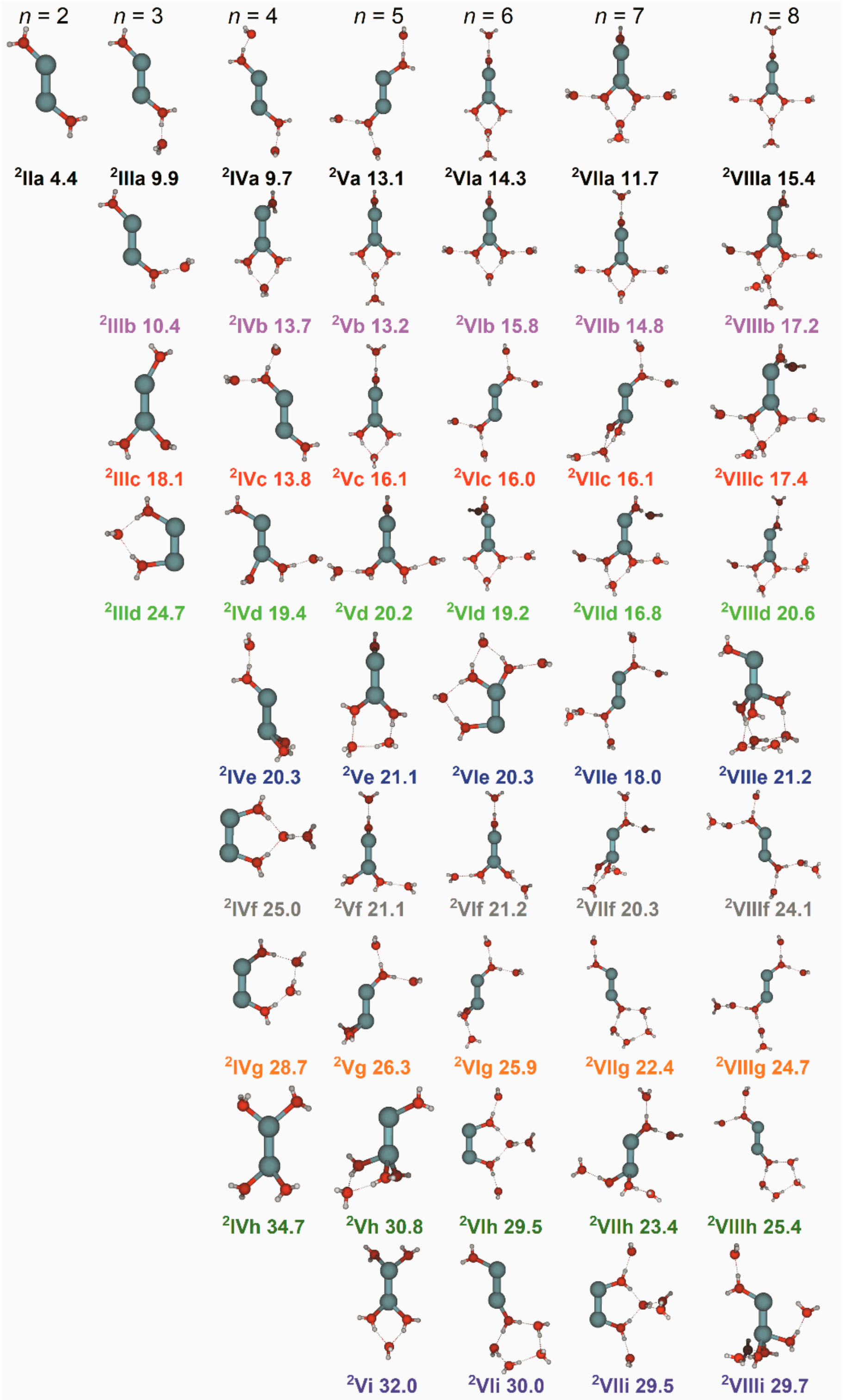

Figure S8. Low-lying isomers of $Zn_2^+(H_2O)_n$ clusters (n = 2–8) with water molecules bound to both Zn atoms calculated at the B3LYP/aug-cc-pVDZ level of theory along with relative energy given in kJ mol^{-1} inclusive of zero-point energy.

Cartesian Coordinates (Å) and electronic energies (Hartee), inclusive of zero-point energy, of optimised $Zn_2^{+}(H_2O)_n$ clusters (n = 1-19) calculated at the B3LYP/aug-cc-pVTZ level of theory.

Ia

E= -3635.152988
Zn 0.801080 -0.000159 -0.001232
O 2.925629 0.000248 0.001914
H 3.499859 0.782039 0.002257
H 3.500004 -0.781438 0.002272
Zn -1.814576 0.000073 0.000570

IIa

E= -3711.599567
O -2.153296 -0.827019 -0.000204
Zn -0.189612 -0.279575 0.000170
O -4.132620 0.873662 -0.000035
H -2.474237 -1.739685 -0.000652
H -2.941571 -0.205668 -0.000181
H -4.667919 1.092083 -0.774203
H -4.667903 1.091734 0.774243
Zn 2.357578 0.259188 -0.000080

IIb

E= -3711.598798
Zn 0.550423 0.019684 -0.000821
O 2.259276 -1.424744 0.000558
H 2.436404 -1.980923 0.775056
H 2.433994 -1.984768 -0.771708
O 2.140247 1.463417 0.000989
H 3.078144 1.221893 -0.001196
H 2.082333 2.429833 0.000243
Zn -2.057991 -0.019531 0.000329

^{2}IIa

E= -3711.597885
Zn 1.204118 -0.506154 0.000074
Zn -1.175231 0.444096 0.000195
O -3.182674 -0.397686 -0.000529
H -3.997569 0.126194 -0.000550
H -3.443660 -1.331025 0.000180
O 3.101428 0.571612 -0.000322
H 3.252760 1.528656 -0.000908
H 3.971830 0.146510 0.000026

IIIa

E= -3788.045017
O 4.034559 -0.000663 0.000113
O 1.627872 -1.424170 -0.000490
Zn 0.035036 -0.000309 -0.000222
O 1.627580 1.425897 -0.000286
H 4.621919 -0.001245 0.769810
H 4.622082 -0.001200 -0.769459
H 2.574906 -1.175787 -0.000099
H 1.553330 -2.387326 -0.000137
H 1.550987 2.388900 -0.000510
H 2.575108 1.179876 -0.000162
Zn -2.562317 -0.000082 0.000417

IIIb

E= are -3788.042633
O -1.849829 0.000124 -0.001158
Zn 0.135078 -0.000452 -0.001819
Zn 2.727039 0.000136 0.001106
O -3.225964 2.272230 0.001431
O -3.226821 -2.271425 0.001452
H -2.414426 -0.816732 -0.000010
H -2.414030 0.817254 -0.000002
H -3.654490 -2.663736 -0.770498
H -3.653388 -2.663219 0.774280
H -3.653884 2.664391 -0.770460
H -3.652379 2.664106 0.774303

IIIc

E= -3788.042437
O -0.742362 2.438815 -0.394719
Zn -0.089194 0.434341 0.085112
O -2.101434 0.142718 0.698169
O -3.481329 -1.825738 -0.426094
H -1.674986 2.698177 -0.375797
H -0.238695 3.171968 -0.775578
H -2.339009 0.203678 1.634382
H -2.631149 -0.609444 0.315193
H -4.320343 -1.690136 -0.885197
H -3.447670 -2.763636 -0.197822
Zn 2.264289 -0.669574 -0.042913

IIId

E= -3788.040574
Zn 0.374154 0.001936 0.000038
H 2.425554 -1.580618 -1.053551
H 1.565564 -2.603567 -0.264793
O 1.823385 -1.670776 -0.299499
O 1.828428 0.573014 1.594582

H 1.574013 1.070504 2.385528
H 2.429318 -0.126734 1.892269
O 1.829009 1.091705 -1.295401
H 2.434588 1.695304 -0.838999
H 1.574599 1.530779 -2.120200
Zn -2.235828 0.000157 0.000038

[2]IIIa

E= -3788.041254
O -3.609284 -0.856510 0.000165
Zn -1.839151 0.456692 -0.000179
Zn 0.605998 -0.255452 0.000044
O 2.423414 0.754882 0.000406
O 4.765953 -0.465006 -0.000297
H -4.522692 -0.535380 0.000285
H -3.648658 -1.824274 0.000573
H 2.542826 1.714188 0.001299
H 3.322936 0.326199 0.000129
H 5.330000 -0.591804 -0.773807
H 5.329530 -0.593073 0.773347

[2]IIIb

E= -3788.041048
O 3.613427 -0.057950 -0.000072
Zn 1.526002 0.668278 0.000057
Zn -0.499953 -0.872802 -0.000089
O -2.573767 -0.790124 0.000203
H 4.389087 0.521305 0.000191
H 3.940438 -0.969510 -0.000098
H -3.149469 0.024015 0.000187
H -3.144905 -1.569819 0.000779
O -4.021298 1.421170 -0.000085
H -4.481809 1.772085 -0.773223
H -4.481711 1.772873 0.772752

[2]IIIc

E= -3788.038116
O -2.579575 1.431283 -0.136295
Zn -0.888615 -0.030227 0.388215
Zn 1.479774 -0.002438 -0.547865
O -2.477858 -1.427642 -0.166023
H -2.852633 2.081293 0.528158
H -2.600299 1.899241 -0.984715
H -3.396146 -1.123530 -0.204375
H -2.493425 -2.388615 -0.053629
O 3.309024 0.047380 0.731016
H 3.384629 0.055729 1.696261
H 4.210364 0.047660 0.378218

[2]IIId

E= are -3788.035594
Zn -0.963678 1.284128 0.000116
Zn -0.951979 -1.292373 -0.000398
O 1.139866 -2.035290 0.001680
O 1.122177 2.045107 0.000427
O 3.149184 0.013500 -0.000865
H 1.923382 -1.449245 0.000110
H 1.446705 -2.952399 -0.000369
H 1.910543 1.465647 -0.000112
H 1.421249 2.964758 0.000100
H 3.739334 0.016350 -0.768970
H 3.738653 0.015708 0.767771

IVa

E= -3864.486863
O 1.064775 -2.095556 -0.001588
Zn -0.148857 -0.335076 0.000258
O 1.629034 0.715080 -0.000449
Zn -2.707330 0.074077 0.000042
O 1.860666 3.377191 -0.000199
O 3.715423 -1.268001 0.000743
H 2.043841 -2.062900 0.000387
H 0.775584 -3.016661 0.002136
H 2.500870 0.282165 0.000045
H 1.738357 1.698044 -0.000299
H 1.946652 3.949203 -0.772977
H 1.945121 3.949021 0.772880
H 4.288593 -1.398864 0.769392
H 4.287424 -1.399750 -0.768635

IVb

E= -3864.486758
O 3.300534 0.000321 -0.772864
O 1.008613 -1.408212 -0.386629
Zn -0.548152 -0.000051 -0.120489
O 1.008601 1.407898 -0.386987
Zn -3.110424 0.000052 0.280144
O 5.266941 -0.000077 1.056672
H 3.726894 0.000520 -1.640096
H 4.036405 0.000268 -0.109694
H 1.941033 1.126205 -0.538590
H 0.954723 2.371469 -0.379966
H 0.954905 -2.371807 -0.381329
H 1.940809 -1.126217 -0.538867
H 5.761447 0.771622 1.360083
H 5.763541 -0.771533 1.357272

IVc

E= -3864.484963
O 1.435136 -1.775544 0.181487
Zn -0.000489 -0.245971 0.000661
Zn 0.002502 2.347329 -0.000133
O -1.438267 -1.773333 -0.180207
O -4.046663 -1.305179 0.076932
H 1.298475 -2.543987 0.752023
H 2.413713 -1.618635 0.117112
H -1.301838 -2.543244 -0.748812
H -2.416734 -1.615130 -0.117313
H -4.583236 -1.670846 0.791725
H -4.664969 -1.023142 -0.608947
O 4.043781 -1.310783 -0.079796
H 4.662882 -1.028333 0.605197
H 4.579412 -1.678704 -0.794138

IVd

E= -3864.484046
O -1.016473 1.580388 0.240890
Zn 0.377203 0.071377 0.018267
Zn 2.970403 -0.102444 -0.014783
O -1.160158 -1.412778 -0.218550
O -3.591695 1.425505 -0.241071
O -3.951342 -1.383993 0.218934
H -1.991594 1.568025 0.023149
H -0.785857 2.459732 0.566164
H -2.126023 -1.406895 -0.039104
H -0.902380 -2.330636 -0.375679
H -4.270329 -1.573509 1.113201
H -4.478777 -1.949694 -0.363055
H -3.982874 0.537082 -0.192130
H -4.132999 1.954936 -0.838682

IVe

E= -3864.483181
O -3.587497 -1.410142 0.028017
O -1.580981 -0.075677 -1.447017
Zn 0.023598 0.305975 -0.002106
O -0.402951 2.459553 -0.091128
O -1.639645 0.088858 1.426089
H -3.665460 -2.373076 0.085792
H -4.498032 -1.084164 -0.010834
H -2.384356 -0.494174 1.175842
H -1.476207 -0.038843 2.370179
H -1.417421 -0.202880 -2.390702
H -2.336464 -0.641246 -1.193876
H 0.256648 3.165650 -0.054115
H -1.255984 2.852080 0.143055
Zn 2.458598 -0.628778 0.020338

IVf

E= -3864.482449
Zn 0.036191 -0.465000 -0.022793
O 2.070882 0.159204 -0.217765
O 0.885083 -1.790494 1.579327
H 0.389974 -2.067157 2.363730
H 1.756744 -1.503286 1.890261
H 0.833820 -3.012333 -0.840799
H 0.211404 -2.425107 -2.134442
O 0.672281 -2.184601 -1.317889
H 2.564149 -0.244222 -0.945561
H 2.349239 1.109616 -0.167992
O 2.822032 2.720839 -0.010585
H 2.641652 3.437089 -0.632426
H 3.550420 3.025075 0.545601
Zn -2.232846 0.813025 0.011357

IVg

E= -3864.479622
O -1.128340 0.000111 -0.568644
Zn 0.802211 -0.000067 -0.153861
O -2.936608 -1.857991 -0.085008
O -2.936774 1.858071 -0.084976
O -5.061925 -0.000063 0.557035
H -1.722674 0.793247 -0.445536
H -1.722683 -0.793044 -0.445675
H -3.782253 1.411233 0.103241
H -3.145222 2.674088 -0.555545
H -3.782214 -1.411225 0.102798
H -3.144789 -2.674174 -0.555410
H -5.317796 -0.000346 1.490387
H -5.897458 0.000023 0.069303
Zn 3.365264 0.000040 0.210167

^{2}IVa

E= -3864.483155
O 2.965026 -0.808537 -0.000399
Zn 1.216576 0.351272 -0.000766
Zn -1.216593 -0.350664 -0.000084
O -2.965397 0.808647 -0.000131
O 5.415810 0.217793 0.001925
H 3.891534 -0.450442 -0.002240
H 3.013411 -1.773686 -0.003605
H -3.014055 1.773789 -0.000385
H -3.891791 0.450270 0.000378

H 5.983583 0.335610 -0.769914
H 5.980298 0.329939 0.777012
O -5.415399 -0.219544 0.001253
H -5.981705 -0.335595 -0.771934
H -5.981061 -0.334985 0.775004

^{2}IVb

E= -3864.481653
O -4.390251 -0.008415 -0.191658
O -1.968218 -1.439817 -0.074060
Zn -0.374173 -0.027058 0.366136
Zn 1.971132 0.094398 -0.607339
O -1.985266 1.416930 0.148999
H -4.871517 0.053703 -1.029083
H -5.072801 -0.067007 0.491857
H -2.912355 -1.191142 -0.122045
H -1.902835 -2.402868 -0.046295
H -1.931436 2.365244 0.323016
H -2.926320 1.167267 0.062353
O 3.750942 -0.162787 0.783733
H 3.783881 -0.264327 1.745607
H 4.666990 -0.128346 0.474573

^{2}IVc

E= -3864.481596
O -2.189339 0.012427 -0.000187
Zn -0.233576 -0.488560 0.000396
Zn 2.097104 0.513955 -0.000763
O 3.911062 -0.788263 0.001033
O -3.023478 2.564890 0.000651
H -2.931489 -0.640070 -0.000254
H -2.546601 0.934297 0.000256
H 3.973620 -1.754320 0.002297
H 4.816495 -0.446668 0.000744
H -3.362322 3.036096 -0.770978
H -3.361453 3.036852 0.772196
O -4.109387 -1.863146 -0.000484
H -4.602897 -2.167475 0.771390
H -4.602077 -2.167827 -0.772743

^{2}IVd

E= -3864.479461
O 3.492373 -0.789384 -0.785456
Zn 1.728409 -0.348778 0.557996
Zn -0.462199 0.553097 -0.355126
O -2.420495 -0.000577 0.135415
O -3.487634 -2.442976 -0.036182
H 4.334126 -1.134454 -0.455928
H 3.558299 -0.764503 -1.750838
H -2.840028 -0.895399 0.057915
H -3.117754 0.668085 0.124415
H -3.796460 -2.946072 0.727920
H -3.920697 -2.829627 -0.807611
O -1.314075 2.652054 0.126956
H -1.200988 3.053060 1.001569
H -1.164160 3.366398 -0.509388

^{2}IVe

E= -3864.479122
O -2.683378 -0.013329 0.755406
Zn -0.897995 0.016152 -0.370136
Zn 1.495062 -0.064813 0.447489
O 3.102602 1.461692 -0.295472
O 3.028370 -1.384148 -0.441196
O -5.151900 0.030513 -0.257035
H -3.600180 0.002952 0.379387
H -2.754589 -0.103848 1.714790
H 3.134192 -2.336759 -0.309763
H 3.910880 -1.011220 -0.579510
H -5.696262 -0.725181 -0.510158
H -5.704613 0.814864 -0.361400
H 2.964559 1.981109 -1.101362
H 3.468451 2.080068 0.353820

^{2}IVf

E= -3864.477328
O 0.581634 1.986010 0.360238
Zn -1.438859 1.286729 -0.136452
Zn -1.441376 -1.285896 -0.136603
O 0.579555 -1.985906 0.360960
O 2.455471 -0.000706 0.847564
H 1.328547 1.371989 0.550805
H 0.887687 2.893994 0.485770
H 1.327039 -1.372443 0.551002
H 0.885445 -2.894079 0.485515
H 2.810433 -0.000939 1.746999
H 3.247451 -0.001194 0.252915
O 4.598288 -0.001789 -0.784778
H 5.100381 0.769585 -1.076178
H 5.100488 -0.772771 -1.077024

^{2}IVg

E= -3864.475920
Zn 1.826581 0.338670 -0.279131
Zn 0.252183 -1.593849 0.363643
O -1.838073 -1.675540 -0.414440
H -2.347465 -2.492322 -0.507947
H -2.471749 -0.927658 -0.426941

O 1.016892 2.289729 -0.095488
H 0.078388 2.430237 0.195741
H 1.377446 3.123718 -0.423540
O -3.517875 0.632932 -0.460070
H -4.337271 0.562575 0.050841
H -3.800136 0.888536 -1.350337
O -1.503528 2.391405 0.747357
H -2.254999 1.923039 0.344712
H -1.866454 3.039028 1.363242

[2]IVh

E= -3864.473642
O -2.847018 1.408905 0.411988
Zn -1.167061 -0.017882 -0.489967
Zn 1.166924 -0.019791 0.491261
O -2.767478 -1.416841 0.178349
O 2.846104 1.410282 -0.412558
H -3.225754 2.086387 -0.166619
H -2.733390 1.836469 1.273363
H -2.940861 -2.311110 -0.147350
H -3.624974 -1.003040 0.353740
H 2.728207 1.838574 -1.272979
H 3.225308 2.088019 0.165430
O 2.768950 -1.414813 -0.181581
H 3.624354 -0.997106 -0.358011
H 2.946752 -2.308257 0.144006

Va

E= -3940.927277
O 3.790768 0.005081 0.001073
O 1.332452 -1.429390 0.000662
Zn -0.164825 -0.000257 -0.000565
O 1.328979 1.432112 -0.001490
Zn -2.749885 -0.002935 0.000448
O 1.017341 4.101540 0.000229
H 4.377397 0.004352 0.770154
H 4.378754 0.006612 -0.766969
H 2.277468 -1.192745 -0.000240
H 1.229377 -2.410535 0.000197
H 1.223867 2.413075 -0.000721
H 2.274512 1.197402 0.000528
H 0.918919 4.671422 0.772928
H 0.920328 4.672133 -0.772125
O 1.028340 -4.100083 -0.000380
H 0.928780 -4.669826 -0.773035
H 0.928876 -4.670193 0.772018

Vb

E= -3940.927067
O 3.206557 -0.499089 -0.772083
O 0.841457 -1.771114 -0.472213
Zn -0.619822 -0.275527 -0.118205
Zn -3.177250 -0.316642 0.273690
O 0.945358 1.046919 -0.255218
O 5.205778 -0.813587 1.019534
O 0.820767 3.721549 -0.005308
H 3.625475 -0.448230 -1.640981
H 3.944782 -0.609233 -0.124764
H 1.860559 0.742230 -0.420226
H 0.915326 2.028343 -0.164376
H 0.729632 -2.725243 -0.558843
H 1.787255 -1.528565 -0.614117
H 5.748978 -0.113122 1.401598
H 5.648461 -1.643514 1.236568
H 0.837231 4.228337 0.815874
H 0.655120 4.356666 -0.712954

Vc

E= -3940.924477
O 0.850843 -2.004813 0.024657
Zn -0.424985 -0.368204 0.030255
Zn -3.010401 -0.176528 0.020157
O 1.118901 1.015619 -0.027311
O 3.464519 -1.989023 -0.275974
O 0.629121 3.665313 -0.206979
O 3.962462 0.740668 0.347233
H 1.837203 -2.040494 -0.125841
H 0.540389 -2.896068 0.226562
H 2.077831 0.905150 0.123912
H 0.926792 1.981521 -0.085248
H 0.459316 4.145733 -1.027053
H 0.340840 4.245899 0.508497
H 4.301764 0.855938 1.246215
H 4.491448 1.337032 -0.201016
H 3.881087 -1.115446 -0.174986
H 3.998148 -2.499409 -0.896403

Vd

E= -3940.923742
O -0.966369 1.248813 -0.081135
Zn 0.386687 -0.234349 -0.019867
Zn 2.963015 -0.525340 -0.002276
O -1.094161 -1.816684 0.020001
O -3.622551 0.947343 -0.083694
O -0.126557 3.804433 0.117990
O -3.924914 -1.900867 0.102239
H -1.953777 1.177413 -0.104329
H -0.702667 2.195943 -0.009071

H -2.074566 -1.838197 0.055505
H -0.786009 -2.731417 0.052125
H 0.004306 4.416481 -0.616917
H -0.150744 4.345318 0.917212
H -4.326820 -2.189500 0.934083
H -4.359979 -2.423555 -0.586199
H -3.978558 0.043659 -0.110234
H -4.285810 1.530237 -0.471069

Ve

E= -3940.923331
O 1.743628 0.494987 -1.442850
Zn 0.124582 0.029030 -0.002380
O 1.737428 0.500397 1.445057
O -0.518899 2.030734 -0.012704
O -3.072741 2.861961 0.006657
O 4.122296 -0.037708 0.005988
H 1.642379 0.290951 -2.381893
H 2.656021 0.255166 -1.188577
H -1.451047 2.358118 -0.004014
H 0.075061 2.791778 -0.007472
H 1.633028 0.294694 2.383400
H 2.651038 0.261778 1.194053
H 4.576516 -0.892045 0.009515
H 4.827588 0.624885 0.004983
H -3.569133 3.154473 0.781137
H -3.576031 3.154889 -0.763215
Zn -1.509885 -1.998951 0.000877

Vf

E= -3940.922818
O -3.513952 -0.929664 -1.173265
O -1.014857 -2.028337 -0.526741
Zn 0.184759 -0.370780 0.235442
O -1.639449 0.745186 0.269396
O -0.340512 -0.854953 2.332454
O -1.654856 3.440523 -0.000864
H -3.802382 -0.742175 -2.077436
H -4.301978 -1.260036 -0.719404
H -1.908703 -1.868621 -0.888987
H -0.643054 -2.801672 -0.969274
H -1.634761 1.724333 0.152614
H -2.394502 0.390659 -0.234018
H 0.298008 -1.044161 3.033577
H -1.049213 -0.328576 2.729126
H -1.220051 3.983364 -0.670114
H -2.061546 4.056799 0.620932
Zn 2.616148 0.201048 -0.508271

Vg

E= -3940.921897
O -1.096071 -0.865078 1.457512
Zn 0.231085 0.129631 0.000447
O -0.930347 1.893676 -0.001651
O -1.095125 -0.864900 -1.457581
O -3.614017 2.219031 0.000453
O -2.238583 -3.013708 -0.000455
H -0.830058 -0.949752 2.382471
H -1.506731 -1.710963 1.192289
H -0.532187 2.772303 0.000851
H -1.913158 2.003365 -0.000581
H -0.829628 -0.947629 -2.382859
H -1.505141 -1.711480 -1.193679
H -1.890264 -3.916392 0.000234
H -3.201172 -3.112504 -0.001246
H -4.091882 2.550723 0.771453
H -4.092162 2.552278 -0.769705
Zn 2.841766 0.120965 0.000038

Vh

E= -3940.921866
O 2.664774 2.597482 -0.595944
O 0.821097 1.639276 1.331094
Zn -0.200746 -0.001119 0.274317
O 1.512723 0.001284 -1.064296
O 0.824714 -1.639336 1.329817
O 2.670497 -2.593087 -0.596647
H 2.438889 3.288749 -1.234376
H 3.581516 2.776684 -0.344162
H 1.443222 2.183726 0.809713
H 0.343645 2.231328 1.927128
H 2.085792 -0.787329 -1.063618
H 2.083448 0.791625 -1.062973
H 2.446690 -3.284561 -1.235585
H 3.587317 -2.770567 -0.343922
H 1.447806 -2.181833 0.807493
H 0.347981 -2.233272 1.924558
Zn -2.724479 -0.000865 -0.388199

Vi

E= -3940.918477
O -0.470404 -0.000065 -0.570400
Zn 1.454863 -0.000368 -0.175162
O -2.243029 -1.835954 0.028090
O -2.242565 1.836489 0.027634
O -4.214792 0.000623 0.830390
O -6.692713 -0.000482 -0.306844

H -1.061698 0.793623 -0.411172
H -1.061978 -0.793516 -0.410918
H -2.529092 2.595777 -0.493466
H -3.048473 1.342413 0.286982
H -3.048645 -1.341512 0.287671
H -2.530035 -2.594796 -0.493399
H -4.359423 0.001076 1.785667
H -5.115997 0.000277 0.431262
H -7.262151 0.769882 -0.425394
H -7.262856 -0.770811 -0.422185
Zn 4.018082 0.000125 0.168295

[2]Va

E= -3940.922298
O -2.750631 0.078737 0.001751
Zn -0.827365 -0.579242 -0.002527
Zn 1.504860 0.382038 0.002409
O 3.269837 -0.791852 -0.004312
O 5.754948 0.192781 0.001910
O -4.838724 -1.629555 0.003171
H -3.539758 -0.512664 -0.001204
H -3.039067 1.022569 0.000178
H 3.316836 -1.756701 -0.010050
H 4.195530 -0.439705 -0.002205
H -5.338125 -1.929310 -0.766389
H -5.332599 -1.925404 0.777794
H 6.309777 0.357978 -0.770320
H 6.309281 0.349080 0.776346
O -3.408516 2.695877 -0.001995
H -3.711187 3.190443 -0.773807
H -3.710862 3.191933 0.768990

[2]Vb

E= -3940.922255
Zn 2.617153 0.004794 0.477752
Zn 0.168821 -0.002538 -0.194196
O 4.173228 -0.010623 -1.203767
H 5.125098 -0.008790 -1.032204
H 4.062876 -0.020766 -2.164982
O -1.377956 -1.413998 0.304394
H -2.305123 -1.127771 0.465671
H -1.353718 -2.372518 0.197082
O -1.376743 1.418625 0.280238
H -1.351279 2.375276 0.157647
H -2.304133 1.136306 0.446939
O -3.670220 0.007428 0.765960
H -4.027260 0.014670 1.663676
H -4.454167 0.002176 0.164305
O -5.792145 -0.007323 -0.905681
H -6.309768 0.761705 -1.174888
H -6.311061 -0.780841 -1.159076

[2]Vc

E= -3940.921142
O -2.537981 -1.442996 0.049484
Zn -0.951884 -0.002089 -0.443224
Zn 1.388474 0.008684 0.511332
O 3.136446 -0.012727 -0.729264
O 5.683819 -0.002584 0.126763
O -4.955573 -0.001942 0.323360
H -3.478101 -1.189341 0.126377
H -2.491239 -2.394977 -0.105267
H 3.160113 -0.021359 -1.694817
H 4.070224 -0.008652 -0.404622
H -5.403184 0.002544 1.181261
H -5.664251 -0.006225 -0.335245
H 6.199037 0.772676 0.381472
H 6.199695 -0.772852 0.394938
O -2.540927 1.440641 0.033391
H -2.495730 2.391206 -0.130182
H -3.480536 1.185987 0.112973

[2]Vd

E= -3940.919566
O 2.467485 -0.586690 -0.127062
Zn 0.405653 -0.330809 0.332774
Zn -1.343510 1.254937 -0.590522
O -2.331512 2.705871 0.897914
O 0.343914 -2.476400 0.228244
O -1.973382 -3.786665 -0.153339
O 4.272798 1.403887 -0.102286
H 2.754085 -1.263405 -0.754773
H 3.135072 0.143119 -0.146766
H 0.901578 -3.002174 0.817177
H -0.507339 -2.969508 0.120600
H -2.633630 -4.028972 0.507863
H -2.129134 -4.362275 -0.912691
H -2.134801 2.878073 1.829443
H -2.993618 3.357225 0.628320
H 4.497630 2.005452 -0.822960
H 5.011431 1.438619 0.518461

[2]Ve

E= -3940.919235
O -4.326259 -1.423544 0.171915
O -1.491774 -1.393925 -0.147604
Zn 0.041198 0.095893 0.340109
Zn 2.385901 -0.069723 -0.626811
O 4.135291 -0.129575 0.857821
O -1.400607 1.613924 0.252757

O -3.971908 1.381392 -0.325286
H -4.695325 -1.589428 1.051105
H -4.811169 -2.013826 -0.421952
H -4.332586 0.479279 -0.288480
H -4.459525 1.861905 -1.004843
H -1.205085 -2.307336 -0.275709
H -2.459271 -1.416109 0.005888
H -1.250849 2.472990 0.666810
H -2.362164 1.556791 -0.000867
H 5.058165 -0.151862 0.569196
H 4.146890 -0.063668 1.823077

[2]Vf

E= -3940.919240
O -2.927092 0.281975 -0.258575
Zn -1.081603 -0.500192 0.388782
Zn 1.245715 -0.044873 -0.473677
O 2.957014 0.238532 0.773797
O 5.451551 0.734190 -0.081979
O -3.791605 2.811474 -0.009691
H -3.678919 -0.321888 -0.316566
H -3.270323 1.203713 -0.153148
H 2.971826 0.236798 1.739619
H 3.875697 0.422539 0.457065
H -4.006920 3.380229 -0.759438
H -4.213617 3.209594 0.761793
H 6.128231 0.079162 -0.292576
H 5.834455 1.597128 -0.281999
O -2.222617 -2.483558 -0.182097
H -2.258102 -3.186076 0.482935
H -2.043689 -2.930175 -1.022502

[2]Vg

E= -3940.917251
O 2.476660 0.042267 0.028801
Zn 0.534189 -0.596249 -0.027386
Zn -1.747015 0.475593 0.005987
O -3.317777 -0.172082 -1.447377
O 4.563747 -1.678490 -0.091801
O 3.195726 2.650879 0.142987
H 3.256757 -0.557544 -0.014370
H 2.781540 0.979086 0.068906
H -3.484113 0.200471 -2.324387
H -4.179115 -0.358740 -1.046878
H 3.515172 3.096216 0.937671
H 3.534310 3.161923 -0.602721
H 5.023650 -1.973504 -0.887262
H 5.054657 -2.043093 0.654761
O -3.423765 -0.529045 1.369635
H -3.284150 -1.406139 1.755285
H -3.790654 0.012752 2.083004

[2]Vh

E= are -3940.915534
O 3.959330 -1.357174 0.205991
O 1.841235 -0.089544 1.606530
Zn 0.264662 0.393251 0.141906
O 1.857835 -0.255258 -1.369858
Zn -2.016548 -0.565782 -0.493568
O 1.244024 2.338135 -0.347423
O -3.771816 -0.021331 0.952117
H 4.148903 -2.304338 0.260640
H 4.825259 -0.925536 0.191091
H 2.640551 -0.727698 -1.024761
H 1.567380 -0.734142 -2.157244
H 1.649790 -0.254359 2.538701
H 2.639395 -0.598862 1.367238
H 0.807265 3.198616 -0.282296
H 1.750206 2.339519 -1.172737
H -3.851744 0.604374 1.685623
H -4.665289 -0.340261 0.764733

[2]Vi

E= -3940.915102
O 3.149445 -1.492192 -0.182900
Zn 1.633457 -0.007206 0.618004
O 3.404134 1.278780 -0.621330
Zn -0.670761 0.082423 -0.421779
O -2.298736 1.406126 0.264337
O -2.274364 -1.439619 -0.240993
O -4.713180 -0.062731 0.213685
H 3.214820 1.695416 -1.473929
H 3.884721 1.945636 -0.110920
H 3.481588 -2.262223 0.299128
H 3.922020 -0.988605 -0.480783
H -2.253446 2.364768 0.367995
H -3.237548 1.137891 0.281162
H -5.400040 0.064914 -0.455504
H -5.188342 -0.232187 1.039365
H -3.216205 -1.193582 -0.161747
H -2.226844 -2.311475 -0.653918

VIa

E= -4017.366073
O 3.172524 -0.003615 -0.691822
O 0.832869 1.417951 -0.306446
Zn -0.644610 0.003756 -0.064720
O 0.825950 -1.417863 -0.312062
Zn -3.197015 0.000667 0.332838

O 0.584457 -4.111257 -0.321408
H 3.596676 -0.004566 -1.559255
H 3.908543 -0.005658 -0.036062
H 1.762998 1.146585 -0.451346
H 0.752005 2.398768 -0.308797
H 0.741926 -2.398355 -0.311768
H 1.757631 -1.150282 -0.453397
H 0.517050 -4.685759 0.451119
H 0.342674 -4.653552 -1.082315
O 0.599633 4.111432 -0.321132
H 0.357145 4.653822 -1.081729
H 0.535353 4.686388 0.451313
O 5.187082 -0.009039 1.134299
H 5.675553 -0.781497 1.444416
H 5.681060 0.760544 1.442846

VIb

E= -4017.363103
O -0.731751 1.696963 -0.105597
Zn 0.398585 0.014993 -0.014842
Zn 2.940020 -0.466938 0.027157
O -1.220537 -1.293436 -0.058468
O -3.317718 1.713110 0.595763
O 0.481666 4.123651 -0.165101
O -0.917401 -3.982444 0.127096
O -4.052189 -0.854946 -0.396854
H -1.683743 1.776144 0.153604
H -0.319908 2.590322 -0.112714
H -2.173595 -1.120972 -0.182015
H -1.091208 -2.267880 0.003289
H -0.632001 -4.569305 -0.584367
H -0.754515 -4.461515 0.949339
H 0.564946 4.689174 -0.942743
H 0.748944 4.662939 0.589337
H -4.608375 -1.410710 0.166716
H -4.398499 -0.973476 -1.292200
H -3.822425 0.961271 0.241545
H -3.924346 2.459172 0.666039

VIc

E= -4017.362923
O 1.624536 0.420834 -1.517224
Zn 0.000355 -0.000300 -0.156466
Zn -0.001165 0.007054 2.439774
O -0.442418 -1.622192 -1.513308
O -1.179656 1.189282 -1.520275
O 3.980012 1.508654 -0.756469
O -3.299122 2.686878 -0.761835
H -1.937620 1.754427 -1.235871
H -0.633525 1.723068 -2.113535
H -1.178555 -1.417516 -2.106066
H -0.553412 -2.559971 -1.225152
H 1.814624 -0.321694 -2.106856
H 2.492506 0.795061 -1.231980
H 4.433091 2.217949 -1.229031
H 4.605222 1.182088 -0.097723
H -3.328364 3.393726 -0.105341
H -4.140805 2.722312 -1.233011
O -0.680725 -4.203514 -0.745107
H -0.292491 -4.952153 -1.214712
H -1.277393 -4.579472 -0.086214

VId

E= -4017.361982
O 3.756356 -0.626656 -1.319679
O 1.032355 -0.889837 -1.986709
Zn -0.068963 -0.079565 -0.250626
O -0.581182 -2.004897 0.469040
O 1.798622 -0.287235 0.795478
O -3.068702 -2.909084 1.013983
O 2.379031 1.273085 2.950329
H 0.699569 -0.800957 -2.888633
H 2.004892 -0.802890 -2.017362
H 1.995835 0.288225 1.569688
H 2.605413 -0.328335 0.251703
H 4.331638 0.087783 -1.626447
H 4.311372 -1.418928 -1.323095
H 0.049869 -2.468640 1.034109
H -1.486209 -2.324953 0.694152
H 2.216012 2.217323 3.065871
H 2.664080 0.943756 3.811690
H -3.655943 -2.683988 1.745838
H -3.497424 -3.628710 0.534560
Zn -1.756736 1.895475 -0.423761

VIe

E= -4017.361623
O 2.962949 1.113172 0.889856
O 0.307117 1.702993 0.087750
Zn -0.697168 -0.120196 0.214230
Zn -3.111023 -0.956050 -0.183717
O 1.056450 -0.948207 0.973308
O 2.634721 -2.504037 -0.552127
O 4.728980 -0.412134 -0.791504
H 3.418536 1.590466 1.594980
H 3.659680 0.732287 0.319471
H 1.264400 1.769397 0.261171
H -0.056410 2.586432 -0.153915
H 1.530232 -1.652631 0.465595
H 1.746244 -0.285368 1.178236

H 2.841588 -3.446069 -0.529052
H 3.471417 -2.031942 -0.695926
H 4.850333 -0.112180 -1.703493
H 5.623176 -0.593881 -0.469486
O -0.703261 4.116491 -0.575008
H -1.135502 4.736070 0.025718
H -0.863604 4.448595 -1.466867

VIf

E= -4017.361574
O 0.158539 3.698720 -1.092553
O -0.957317 2.161595 1.008168
Zn -0.604979 0.086561 0.329209
O 0.941795 -0.366122 1.789741
O 0.977341 0.917031 -0.893159
O 3.277928 -0.022069 0.388749
O 4.564289 -2.109606 -0.759510
H 1.887757 0.713197 -0.592082
H 0.929447 1.869567 -1.088350
H -1.781671 2.409905 1.446719
H -0.743322 2.864769 0.364075
H -0.337879 3.943278 -1.886181
H 0.705446 4.470253 -0.889520
H 5.012826 -2.835748 -0.308723
H 4.821269 -2.171301 -1.687869
H 1.864417 -0.368368 1.447829
H 0.848895 -1.108540 2.400134
H 3.959800 0.553429 0.757989
H 3.761848 -0.776074 -0.026237
Zn -2.482669 -1.546586 -0.444851

VIg

E= -4017.361336
O 3.560619 0.825745 -0.716142
O 0.917873 1.716083 -0.068523
Zn -0.176659 -0.030700 0.341740
O 0.397387 -0.043576 2.504652
O 1.659955 -1.141125 0.171230
O -0.121710 4.149351 -0.664179
O 1.746643 -3.650098 -0.879771
H 1.671994 -2.046067 -0.216857
H 2.424663 -0.656310 -0.190798
H 0.536675 2.592431 -0.301961
H 1.846310 1.681941 -0.356719
H 3.916849 0.858230 -1.614604
H 4.302564 1.058868 -0.141244
H 1.064255 -0.719209 2.694033
H -0.237486 -0.065741 3.233605
H -0.614979 4.416062 -1.449563
H -0.205440 4.875915 -0.034421
H 1.254701 -3.994039 -1.635606
H 2.142153 -4.417354 -0.447759
Zn -2.602954 -0.583825 -0.418948

VIh

E= -4017.360533
O -2.450842 2.416297 -1.433367
O -1.734499 -0.324639 -1.047039
Zn 0.100242 -0.196568 0.127335
O -1.163359 -0.905813 1.826663
O -0.279698 1.866744 0.385787
O 1.651400 3.690922 0.958815
O -3.224820 -2.305915 0.443840
H -2.297918 2.783458 -2.315159
H -3.251483 2.853613 -1.112687
H -2.179963 0.518891 -1.252079
H -2.409444 -0.948711 -0.724792
H 0.433889 2.518190 0.573824
H -0.944017 2.295397 -0.181798
H -1.872814 -1.527474 1.573759
H -0.748947 -1.255441 2.626527
H -4.144837 -2.225520 0.731349
H -3.132920 -3.222328 0.147412
H 2.477252 3.871388 0.493238
H 1.708897 4.162234 1.799166
Zn 2.365653 -1.314247 -0.508547

VIi

E= -4017.354270
O -0.206872 -0.171942 -0.628988
Zn 1.693372 -0.110975 -0.188081
O -2.037793 1.223660 0.502614
O -1.728011 -2.314799 -0.528027
O -3.136811 3.642569 -0.296294
O -3.777821 -1.037104 0.877503
O -6.351641 -1.033630 -0.060349
H -0.880485 0.472977 -0.220168
H -0.698031 -1.041419 -0.694030
H -2.418275 2.069483 0.189718
H -2.777993 0.608525 0.660624
H -2.547961 -2.002709 -0.088340
H -1.996631 -2.900415 -1.245516
H -3.831233 -1.306310 1.803481
H -4.707060 -1.036674 0.553888
H -6.969096 -0.294339 0.000016
H -6.888641 -1.811594 -0.255248
H -3.204806 4.005853 -1.187720
H -3.169072 4.401186 0.299659
Zn 4.239991 -0.010178 0.229813

[2]VIa

E= -4017.360615
Zn -2.009304 0.001649 -0.526571
Zn 0.397762 -0.002440 0.232605
O 1.941500 1.425377 -0.323143
O 1.944103 -1.424630 -0.328755
O 4.256353 0.002794 -0.735770
O 6.366173 -0.000029 0.964286
O -3.622691 -0.001536 0.903913
O -6.259718 0.000349 0.341195
H -6.777775 0.774031 0.087165
H -6.777509 -0.770376 0.077809
H -3.543504 -0.007250 1.866378
H -4.585013 -0.001011 0.681612
H 2.873965 -1.133670 -0.456557
H 1.935137 -2.371173 -0.141848
H 2.872280 1.137797 -0.451547
H 1.929592 2.371670 -0.135074
H 5.032590 0.001692 -0.126154
H 4.623024 0.003896 -1.629431
H 6.879213 -0.771426 1.235185
H 6.878527 0.770958 1.237641

[2]VIb

E= -4017.360048
O -1.622716 -1.450433 0.069722
Zn -0.115633 0.000943 0.232116
O -1.660968 1.410809 0.069769
Zn 2.262315 0.031115 -0.641807
O 3.890844 0.026159 1.036881
O -1.463149 4.113798 0.044468
O -4.112450 -0.052385 -0.083859
H -2.567598 -1.224028 0.012488
H -1.515915 -2.428409 0.064212
H -2.599302 1.157880 0.015217
H -1.582714 2.391546 0.068220
H -1.404795 4.700311 0.808541
H -1.283794 4.660858 -0.730063
H -4.642917 -0.057064 -0.892368
H -4.752538 -0.061982 0.640867
H 3.820156 0.041759 2.001371
H 4.834644 0.043511 0.828069
O -1.320918 -4.143936 0.039137
H -1.132978 -4.686429 -0.736539
H -1.257839 -4.731798 0.801769

[2]VIc

E= -4017.359969
O -3.037451 0.060488 -0.001300
Zn -1.098618 -0.608216 0.000807
Zn 1.098359 0.607402 0.001775
O 3.037555 -0.060569 -0.002472
O 3.726255 -2.684636 0.002798
O -5.190234 -1.592742 -0.004268
O 5.188833 1.595246 -0.004608
H -3.827341 -0.526375 -0.002317
H -3.328120 1.002017 -0.000021
H 3.328727 -1.001938 -0.000239
H 3.827059 0.526734 -0.003063
H -5.621557 -1.977648 -0.776863
H -5.623029 -1.978308 0.767174
H 5.622876 1.976818 -0.777335
H 5.621520 1.981452 0.766575
H 4.052694 -3.164744 -0.768346
H 4.056149 -3.163043 0.773533
O -3.724297 2.684973 0.002103
H -4.053156 3.164706 -0.768235
H -4.053347 3.162655 0.773632

[2]VId

E= -4017.358743
O -1.696426 -2.146034 -0.269668
Zn -0.465480 -0.371719 0.175791
Zn 1.554275 0.396404 -1.120741
O -2.336141 0.589680 0.356107
O -2.812983 3.231349 0.733306
O -4.385182 -1.436992 0.136970
H -2.663365 -2.155177 -0.127755
H -1.378363 -3.057340 -0.265117
H -3.187222 0.120508 0.327733
H -2.498665 1.549560 0.499933
H -2.792600 3.709852 1.571081
H -2.840201 3.902160 0.040013
H -4.992223 -1.451926 -0.615906
H -4.916113 -1.706251 0.899120
O 4.492080 -0.662876 2.033002
O 3.565901 0.342132 -0.311562
H 4.980274 -1.491666 2.114346
H 4.773184 -0.113765 2.775502
H 3.911483 -0.023067 0.537860
H 4.321969 0.638471 -0.833574

[2]VIe

E= -4017.358340
Zn 1.813256 -1.654403 -0.001171
Zn -0.335008 -0.288497 -0.001185
O -2.402285 -0.111455 0.001217
H -3.068009 -0.839459 0.001815

H -2.853067 0.751982 0.000402
O -3.042802 2.683421 0.000321
H -3.473312 3.082337 0.769069
H -3.474128 3.084023 -0.767089
O -0.301633 1.881165 -0.003785
H 0.517313 2.410479 -0.001421
H -1.085725 2.457572 -0.001602
O 2.334650 2.983075 0.001151
H 2.612024 3.503356 0.768232
H 2.614427 3.498708 -0.768201
O 3.324734 0.191417 0.004994
H 4.285361 0.093979 0.007112
H 3.127874 1.144508 0.004784
O -4.237445 -2.086348 0.002648
H -4.476626 -2.615001 -0.768900
H -4.475314 -2.615686 0.774130

^{2}VIf

E= -4017.358016
O -3.148732 -0.231553 0.846621
Zn -1.528536 -0.055256 -0.564524
Zn 0.891007 -0.052055 0.136981
O 2.340160 1.423880 -0.303454
O 2.420941 -1.362635 -0.785385
O -5.785398 -0.209754 0.278784
O 2.464868 4.006474 0.469168
O 3.618042 -3.274470 0.713432
H -4.110441 -0.220338 0.622506
H -3.071814 -0.284878 1.807824
H 2.220722 -1.761820 -1.642664
H 2.837197 -2.074744 -0.242352
H 3.209283 1.125591 -0.600382
H 2.407444 2.363869 -0.011308
H 3.198956 -3.982875 1.217324
H 4.561749 -3.476508 0.698143
H 2.389812 4.759719 -0.129604
H 2.817941 4.353331 1.297651
H -6.313474 0.571969 0.075100
H -6.300542 -0.969523 -0.019280

^{2}VIg

E= -4017.356223
Zn 1.021868 -0.706172 -0.162374
Zn -1.290437 0.117551 0.378230
O -3.019063 -0.591126 -0.806477
O -5.034115 1.124313 -1.370077
O -2.557180 -0.829668 1.962328
O 3.292664 2.814177 0.549599
O 5.196781 -1.048649 -0.768606
O 2.867365 0.215911 -0.143049
H -2.833208 -1.065843 -1.627556
H -3.745844 0.044392 -1.015986
H -5.977921 0.926524 -1.330823
H -4.962000 2.046382 -1.645574
H -3.247475 -1.439935 1.667152
H -2.357514 -1.035976 2.885242
H 3.678877 3.091075 1.389816
H 3.508193 3.510755 -0.082938
H 5.712245 -1.615206 -0.181611
H 5.542817 -1.197221 -1.657103
H 3.056348 1.150699 0.101925
H 3.710959 -0.236681 -0.367940

^{2}VIh

E= -4017.354836
O 2.025185 0.306431 -0.244058
Zn 1.282577 -1.633500 0.200308
Zn -1.286722 -1.630322 0.199737
O -2.025426 0.313878 -0.232900
O 0.002295 2.220938 -0.655785
H 1.420261 1.062723 -0.396987
H 2.959474 0.598625 -0.350637
H -1.418766 1.067735 -0.390872
H -2.958870 0.606357 -0.345704
H 0.000884 2.602001 -1.543660
H 0.004441 2.993241 -0.042693
O 4.614471 1.096020 -0.537309
H 5.248975 1.150722 0.188313
H 5.136686 0.933243 -1.332940
O -4.613068 1.103887 -0.540275
H -5.247951 1.165084 0.184481
H -5.135225 0.938625 -1.335424
O 0.008518 4.349530 1.041213
H 0.780234 4.838367 1.352390
H -0.761603 4.832472 1.365276

^{2}VIi

E= -4017.354641
O -4.825114 -0.724684 0.647860
Zn -3.131888 0.673143 0.107104
Zn -0.859924 -0.402744 -0.208464
O 1.051067 -0.262887 -0.728336
O 2.730925 -2.236256 -0.481276
O 2.549579 1.958192 -0.617940
O 4.939132 -1.280057 0.787739
O 5.154031 1.633449 0.619498
H -5.737025 -0.427446 0.773448
H -4.825840 -1.686600 0.753505
H 1.584433 0.573225 -0.713777
H 1.687449 -1.040023 -0.675161

H 3.550890 -1.964253 -0.008961
H 3.011252 -2.777449 -1.228666
H 2.582139 2.598843 -1.338332
H 3.454011 1.899579 -0.258527
H 5.940682 1.862266 0.104898
H 5.220951 2.159464 1.428624
H 5.077322 -0.320617 0.862339
H 5.411128 -1.691014 1.521042

VIIa

E= -4093.802415
O 2.819433 0.000135 -0.000248
O 4.402444 -0.058202 2.268422
O 0.505936 -1.407268 -0.031318
Zn -0.979264 0.000070 0.000330
Zn -3.560321 -0.000091 -0.000333
O 0.505871 1.407475 0.030592
O 0.302693 -4.118836 -0.020122
O 0.302975 4.119103 0.022144
H 3.405054 0.018927 -0.786124
H 3.405443 -0.018968 0.785305
H 1.446967 1.115075 0.021047
H 0.436433 2.387150 0.026725
H 0.436409 -2.386933 -0.026193
H 1.447058 -1.114937 -0.021518
H 0.107500 -4.662856 0.752809
H 0.101772 -4.665856 -0.789466
H 0.107565 4.663193 -0.750685
H 0.102072 4.665988 0.791587
H 4.862036 -0.827802 2.625666
H 4.773374 0.705577 2.726864
O 4.401045 0.057762 -2.269608
H 4.861307 0.827017 -2.626731
H 4.771360 -0.706294 -2.728089

VIIb

E= -4093.801496
O 0.065656 1.812108 -0.274720
Zn -0.792668 -0.005611 -0.088752
Zn -3.221508 -0.784207 0.343174
O 0.923470 -1.113763 -0.416172
O -1.415850 4.021957 0.286688
O 3.592263 -0.327786 -0.575659
O 2.611632 2.265868 -0.899130
O 0.823913 -3.835524 -0.574904
H 0.998386 2.028772 -0.538031
H -0.427379 2.640002 -0.080388
H 1.867982 -0.842692 -0.445610
H 0.880105 -2.094745 -0.458668
H 0.691284 -4.447784 0.159425
H 0.588679 -4.323942 -1.373680
H -1.332576 4.563147 1.081543
H -1.868927 4.572100 -0.364199
H 4.145992 -0.389092 0.237944
H 4.097249 -0.773094 -1.267862
H 3.110460 1.424844 -0.907367
H 2.919574 2.787979 -1.648925
O 5.119142 -0.403774 1.672358
H 5.627772 0.349459 1.997153
H 5.364869 -1.153118 2.228341

VIIc

E= -4093.801447
O -0.538966 0.289242 -1.383077
Zn 0.957172 -0.029636 0.127272
O -0.188786 1.330436 1.414846
O -0.149590 -1.827264 0.697751
O -2.894796 -1.576133 1.080107
O -2.976808 1.169367 1.391340
O -3.281496 1.583855 -1.262503
O -2.934261 -1.148934 -1.688426
H 0.211555 1.819695 2.144150
H -1.164437 1.348546 1.529539
H -0.986688 1.146131 -1.454162
H -1.227593 -0.375129 -1.593163
H -1.096111 -1.833285 0.961888
H 0.298544 -2.531152 1.183536
H -3.440607 -2.142030 1.640872
H -3.060047 -0.648593 1.363664
H -3.538218 1.673701 1.994071
H -3.210133 1.460104 0.474615
H -3.185667 -1.811779 -2.343751
H -3.080041 -1.544941 -0.805598
H -3.832212 2.208950 -1.750076
H -3.451571 0.696912 -1.635765
Zn 3.525524 0.095246 -0.250943

VIId

E= -4093.799229
O 2.760191 -0.449221 -0.152657
O 0.253043 -1.479741 0.346839
Zn -1.030412 0.057643 -0.189933
O 0.615132 1.111346 -0.878112
Zn -3.586432 0.437531 -0.208154
O -0.328168 -3.912942 1.398821
O 1.543319 3.223635 0.517191
O 3.830757 1.700110 1.555149
H 3.439590 -0.872807 -0.721625
H 3.238038 0.124378 0.471879

H 1.222727 -1.343959 0.298642
H 0.043028 -2.358933 0.734117
H 0.833828 1.984781 -0.471158
H 1.446983 0.590175 -0.809915
H 1.604977 4.167643 0.329320
H 2.398137 2.950391 0.891545
H 3.792604 1.591158 2.515562
H 4.740082 1.974966 1.372971
H -0.594764 -4.685923 0.885975
H -0.588925 -4.093572 2.310337
O 4.697847 -1.578147 -1.737150
H 5.122976 -2.432921 -1.594851
H 4.829074 -1.370937 -2.670818

VIIe

E= -4093.798781
O 2.420065 1.637798 1.005615
O -0.274096 1.882621 0.122884
Zn -1.020763 -0.067471 0.234988
O 0.767265 -0.636692 1.044989
Zn -3.277549 -1.197464 -0.312118
O 2.066690 -2.790068 0.320969
O 4.277400 -1.813932 -0.988867
O 4.765349 1.099857 -0.570087
H 2.685709 2.075035 1.824828
H 3.237372 1.532389 0.478788
H 0.657259 2.082303 0.328915
H -0.748696 2.706090 -0.133985
H 1.227178 -1.498271 0.825051
H 1.453650 0.052892 1.138426
H 2.295627 -3.523192 0.903942
H 2.884697 -2.541593 -0.161494
H 4.880424 1.661562 -1.349481
H 5.610779 1.147840 -0.101359
H 4.472989 -0.863099 -0.994695
H 4.685007 -2.187349 -1.778991
O -1.579278 4.146960 -0.583500
H -2.131409 4.680792 0.001323
H -1.808359 4.410297 -1.483401

VIIf

E= -4093.798476
O -0.919531 -1.130409 -0.283042
Zn 0.792748 -0.116275 -0.024212
O -0.073892 1.777093 -0.090405
Zn 3.271043 -0.780557 0.302328
O -3.339714 -0.138066 -0.260676
O -5.529418 -0.803497 1.312473
O -0.768907 -3.799028 -0.827736
O 1.394233 4.045713 0.232776
O -2.670687 2.616929 -0.935236
H -1.857838 -0.784839 -0.242924
H -0.923348 -2.093003 -0.477319
H -0.963303 2.046653 -0.393559
H 0.471387 2.588464 0.019981
H 1.640705 4.418993 1.088276
H 2.014657 4.415701 -0.407595
H -0.876774 -4.510906 -0.185026
H -0.942991 -4.192594 -1.691720
H -2.801681 2.787920 -1.878032
H -3.031275 3.392132 -0.483672
H -3.382819 0.819544 -0.401480
H -4.086299 -0.384426 0.320916
H -5.580493 -0.956516 2.263749
H -6.330348 -1.192049 0.939679

VIIg

E= -4093.789231
O 0.034452 0.000249 0.549738
Zn -1.859373 0.000398 0.125527
Zn -4.406356 0.000733 -0.289454
O 1.639216 1.856725 -0.232806
O 1.638327 -1.857402 -0.232503
O 2.646075 4.042436 1.173864
O 3.495788 -0.000459 -1.356051
O 6.148487 0.000773 -0.620129
H 0.612086 0.802251 0.318327
H 0.611578 -0.802104 0.318513
H 1.991030 2.604437 0.289872
H 2.401490 1.378978 -0.612175
H 2.401367 -1.380272 -0.611034
H 1.988998 -2.605909 0.289814
H 3.491395 -0.000939 -2.321510
H 4.444991 -0.000107 -1.102721
H 6.728876 0.771400 -0.597684
H 6.729523 -0.769354 -0.597294
H 2.751248 4.141539 2.127941
H 2.559940 4.938447 0.825488
O 2.642261 -4.045505 1.172887
H 2.747380 -4.145809 2.126844
H 2.555120 -4.941004 0.823444

^{2}VIIa

E= -4093.797955
Zn -0.377972 0.011721 -0.315509
Zn -2.623900 0.065000 0.854419
O -4.439146 -0.111230 -0.643606
H -5.350414 -0.067146 -0.324002
H -4.481704 -0.040893 -1.607247
O 1.165475 1.407677 -0.426702

H 2.098269 1.123521 -0.507810
H 1.108359 2.387801 -0.413127
O 1.128860 -1.426579 -0.388806
H 2.068305 -1.169347 -0.481998
H 1.043379 -2.404732 -0.377741
O 3.522263 -0.043346 -0.663252
H 4.225713 -0.046050 0.025465
H 3.986990 -0.058017 -1.509265
O 1.029235 4.129105 -0.378470
H 0.876424 4.667194 0.407941
H 0.842338 4.699917 -1.133819
O 0.915221 -4.142959 -0.345328
H 0.751382 -4.675486 0.442669
H 0.694800 -4.704811 -1.098376
O 5.471349 -0.050883 1.245166
H 5.936137 0.717023 1.599333
H 5.910153 -0.824892 1.618676

^{2}VIIb

E= -4093.796783
Zn 0.599650 0.000038 0.271758
Zn -1.739800 0.000777 -0.661558
O -3.382684 0.002452 0.785570
H -4.341851 0.000989 0.559097
H -3.309800 -0.001966 1.748159
O 2.138132 -1.430740 0.036160
H 3.078711 -1.188654 -0.021188
H 2.056048 -2.409083 0.077153
O 2.138181 1.430194 0.033249
H 3.078795 1.187641 -0.021615
H 2.056582 2.408535 0.075658
O 4.615321 -0.000517 -0.142840
H 5.126831 -0.000135 -0.963316
H 5.271512 -0.000821 0.567195
O 1.956859 -4.146371 0.119419
H 1.735013 -4.709446 -0.632298
H 1.845584 -4.693269 0.906656
O 1.956961 4.145381 0.120291
H 1.737892 4.709671 -0.631326
H 1.846053 4.692005 0.907766
O -6.039000 -0.002436 0.214703
H -6.525620 0.769226 -0.100475
H -6.521389 -0.772848 -0.109888

^{2}VIIc

E= -4093.796298
O 2.279671 0.350565 -1.439952
Zn 0.633346 0.598984 0.000283
Zn -1.459907 -0.789440 0.000840
O -3.433528 -0.127624 -0.001916
O 2.281703 0.357375 1.438457
O -4.231066 2.490770 -0.000365
O -5.603493 -1.794957 0.001059
O 4.617344 0.633649 -0.002879
H 3.204731 0.489557 -1.141130
H 2.217523 0.609525 -2.367580
H 3.206200 0.495197 1.137307
H 2.220982 0.621164 2.364820
H 5.277468 -0.099673 -0.001107
H 5.133554 1.449696 -0.005706
H -5.913875 -2.286175 0.771705
H -5.916828 -2.284421 -0.769509
H -3.748870 0.803714 -0.001282
H -4.210392 -0.728940 -0.000853
H -4.616549 2.926531 -0.770579
H -4.618381 2.925639 0.769432
O 6.460805 -1.353883 0.002300
H 6.795694 -1.829145 -0.767942
H 6.794083 -1.826136 0.775089

^{2}VIId

E= are -4093.796028
Zn 0.395797 -0.000162 0.180651
Zn -1.677525 -0.000877 -1.246188
O -3.663074 0.001034 -0.313178
H -4.460182 0.000663 -0.857312
H -3.946062 0.000666 0.630465
O 1.952750 -1.431686 0.260480
H 2.892485 -1.188137 0.324127
H 1.873746 -2.408706 0.193732
O 1.952561 1.431922 0.255597
H 2.892217 1.188895 0.322382
H 1.873053 2.409039 0.191298
O 4.429710 0.000931 0.444891
H 5.076064 0.000998 -0.274108
H 4.952465 0.001021 1.258197
O 1.783929 -4.143179 0.061153
H 1.657374 -4.638510 -0.757352
H 1.591254 -4.757949 0.779559
O 1.782495 4.143983 0.061767
H 1.657924 4.640103 -0.756568
H 1.587785 4.757998 0.780267
O -4.438372 0.000142 2.305334
H -4.798235 -0.770799 2.761142
H -4.798043 0.770706 2.761931

^{2}VIIe

E= -4093.795546
O -3.419373 -0.259846 0.089975
Zn -1.413796 -0.658867 -0.171154

Zn 0.528203 0.920192 -0.019963
O 2.537153 0.796005 -0.249987
O 4.118762 3.008441 -0.030555
O -5.395132 -2.117679 -0.158996
H -4.133998 -0.929252 0.000435
H -3.818524 0.616208 0.296302
H 3.054204 -0.032785 -0.444986
H 3.145611 1.563563 -0.176726
H -5.767239 -2.439338 -0.989254
H -5.664930 -2.749472 0.518707
H 4.461271 3.538586 -0.760445
H 4.591493 3.300413 0.758299
O -4.423648 2.203301 0.663667
H -4.881409 2.764531 0.025593
H -4.758108 2.460827 1.531699
O 3.804980 -1.451973 -0.780654
H 4.065927 -1.712833 -1.670622
H 4.368919 -1.959952 -0.160860
O 5.342048 -2.891169 1.002984
H 5.101335 -3.765773 1.332042
H 6.284917 -2.791086 1.181841

^{2}VIIf

E= -4093.794678
O -2.215488 -2.034600 -0.627350
Zn -0.953272 -0.403768 0.221543
O -2.780271 0.682607 0.185344
Zn 1.246666 0.300967 -0.759736
O 3.126512 0.049968 0.119130
O -3.207717 3.264377 0.937191
O 5.476387 0.776844 -1.094784
O -4.901160 -1.182977 -0.442088
H -3.190104 -1.989676 -0.581531
H -1.957730 -2.964648 -0.605691
H -3.645711 0.264416 0.040740
H -2.917362 1.613284 0.470608
H -3.197908 3.613522 1.836790
H -3.109911 4.026081 0.352666
H -5.401920 -1.079229 -1.262932
H -5.543719 -1.486858 0.213415
H 5.822489 1.677846 -1.095754
H 5.823782 0.359481 -1.892770
H 3.338629 -0.427226 0.951574
H 3.965255 0.311092 -0.319694
O 3.652721 -1.290613 2.456671
H 4.133921 -2.126823 2.487782
H 3.870613 -0.832072 3.277639

^{2}VIIg

E= -4093.793875
O -4.278033 -0.583270 0.437860
Zn -2.535997 0.523608 -0.110995
Zn -0.291631 -0.616841 -0.206420
O 1.635470 -0.350735 -0.672321
O 3.462105 -2.189132 -0.305476
O 2.984640 1.983956 -0.648724
O -6.790186 0.360077 0.550068
O 5.612000 -1.024988 0.906168
O 5.606138 1.886083 0.607895
H -5.199474 -0.227247 0.476012
H -4.314983 -1.524566 0.650763
H 2.106033 0.519757 -0.689171
H 2.323312 -1.072936 -0.570037
H -7.200679 0.804306 1.302026
H -7.397781 0.462122 -0.192711
H 4.261399 -1.837804 0.148103
H 3.778847 -2.754584 -1.019437
H 2.976391 2.591523 -1.397764
H 3.891379 1.998066 -0.291976
H 6.381125 2.140868 0.088014
H 5.628211 2.456129 1.389049
H 5.664747 -0.054839 0.942022
H 6.073232 -1.359717 1.683795

^{2}VIIh

E= -4093.793509
O 2.677185 1.284664 1.160106
Zn 1.130217 -0.206249 0.546566
Zn -1.019672 0.481342 -0.536395
O -2.950939 -0.077195 -0.004658
O 2.845708 -1.053057 -0.395054
O 3.153736 -3.592465 -1.296022
O 2.854946 3.627121 -0.203247
O -5.209671 0.692660 -1.346603
O -3.605018 -1.698795 2.100445
H 2.681435 1.528173 2.095280
H 2.737056 2.132846 0.658545
H 3.688326 -0.732987 -0.047094
H 2.969960 -1.981842 -0.700439
H 3.284178 -4.393947 -0.774636
H 3.407916 -3.814377 -2.200229
H -5.502194 0.332846 -2.192949
H -5.656674 1.542418 -1.249696
H -3.213037 -0.658548 0.743758
H -3.758155 0.204240 -0.487954
H -3.944961 -2.596496 1.999593
H -3.972940 -1.369127 2.929667
H 3.676307 4.064440 -0.459273
H 2.158842 4.026087 -0.739424

²VIIi

E= are -4093.791193
O -1.993623 0.075271 0.242895
Zn -1.286785 -1.906354 0.043888
Zn 1.278821 -1.911114 -0.044280
O 1.992807 0.067624 -0.243716
O 0.003413 1.952583 0.000188
H -1.367968 0.832047 0.145706
H -2.919811 0.389881 0.146375
H 1.369996 0.826759 -0.146404
H 2.920253 0.378794 -0.148093
H 0.100199 2.540956 0.778802
H -0.090011 2.543138 -0.777147
O -4.594991 0.906891 0.010680
H -5.140713 0.778054 -0.775208
H -5.205511 0.896498 0.758547
O 4.597354 0.889168 -0.014051
H 5.208075 0.874670 -0.761683
H 5.142501 0.761909 0.772485
O -0.282663 3.557587 -2.240107
H -0.955349 4.233109 -2.389310
H 0.336057 3.634901 -2.976496
O 0.301223 3.548147 2.245650
H -0.316150 3.625502 2.983155
H 0.969193 4.229648 2.388706

VIIIa

E= -4170.240039
O -0.137097 -1.360401 -1.004249
Zn -1.273038 0.105307 0.078964
O 0.228979 1.566007 -0.474069
O -0.260598 -0.586077 1.894368
O 2.852372 0.948662 -0.089875
O 4.879217 2.781394 0.121286
O 2.424416 -0.932735 -2.036322
O 2.458458 -1.106487 1.800428
O 2.322397 -2.970146 -0.160743
H -0.644882 -0.478666 2.772904
H 0.703643 -0.755387 1.997100
H 0.102820 -2.191297 -0.566925
H 0.652167 -1.115425 -1.533155
H 1.196887 1.473230 -0.293069
H 0.009834 2.505643 -0.463461
H 3.578900 1.604744 -0.006282
H 2.900681 0.359884 0.689950
H 2.989716 -1.308183 2.580844
H 2.512643 -1.896351 1.208293
H 2.703244 -0.793936 -2.949488
H 2.740598 -0.163437 -1.509799
H 2.687495 -3.861003 -0.225530
H 2.590367 -2.488212 -0.968456
H 4.847168 3.643652 0.553501
H 5.745808 2.729276 -0.300272
Zn -3.842370 0.428484 -0.125389

VIIIb

E= -4170.239210
O -0.059455 -0.946172 -1.553791
Zn 1.190204 0.053965 -0.074416
O 0.127515 1.951299 -0.082166
O -0.131280 -0.599778 1.484501
O -2.624591 2.248761 -0.044103
O -3.687703 0.691045 -1.908803
O -2.503544 0.668000 2.198582
O -2.673993 -1.838812 -1.242251
O -2.868301 -1.997065 1.456397
H 0.583263 2.728624 -0.428261
H -0.841280 2.110795 -0.168359
H 0.278952 -1.158350 -2.432189
H -0.976704 -1.298410 -1.488132
H -0.858673 -0.038616 1.831418
H -0.464421 -1.507696 1.528707
H -2.677255 1.099314 3.044575
H -2.676869 1.335347 1.495162
H -2.984920 3.144369 -0.064826
H -3.090739 1.741927 -0.764031
H -2.934796 -2.650356 -1.696184
H -2.829505 -1.996617 -0.277895
H -4.551377 0.735786 -2.334600
H -3.476153 -0.254544 -1.776054
H -3.422677 -2.602952 1.963279
H -2.997501 -1.105744 1.839060
Zn 3.786179 -0.110668 -0.016871

VIIIc

E= -4170.235949
O 0.336091 4.168631 -0.961103
O -0.792151 1.691184 -0.652941
Zn 0.364534 0.051645 0.093493
O -0.869126 0.061598 1.817095
O -0.876544 -1.381594 -0.928942
O -0.740406 -1.692263 3.916242
O 0.130162 -2.884212 -2.999146
O -3.215692 1.146461 0.696598
O -3.741431 -0.739500 -1.349668
H -0.817098 -0.596787 2.543705
H -1.798989 0.327223 1.696777
H -1.644484 1.822668 -0.197429

H -0.369407 2.571929 -0.757245
H 0.936168 4.608698 -0.346598
H 0.435873 4.627068 -1.804435
H -1.795204 -1.171895 -1.171219
H -0.491655 -1.901122 -1.668287
H 0.711833 -2.557219 -3.696744
H 0.343565 -3.820303 -2.897775
H -4.305560 -1.514605 -1.221367
H -3.985550 -0.389261 -2.217621
H -3.625868 0.539948 0.050755
H -3.929060 1.666587 1.085963
H -0.566389 -2.640884 3.882401
H -0.431443 -1.397940 4.782219
Zn 2.951667 -0.156196 0.094235

VIIId

E= -4170.235888
O -2.417671 1.081898 -0.250949
O 0.187656 1.677454 0.419229
Zn 1.264637 0.007927 -0.189129
O -0.469094 -0.739511 -0.966176
Zn 3.726621 -0.757501 -0.059286
O -1.248634 -3.215974 -0.543338
O -3.417033 -2.972480 1.124235
O -4.365750 -0.204547 1.518190
H -2.909016 1.577608 -0.941294
H -3.088251 0.730187 0.361738
H -0.784827 1.701237 0.297686
H 0.511986 2.518661 0.809821
H -0.732146 -1.698567 -0.873272
H -1.269120 -0.191452 -0.810851
H -1.419606 -3.863707 -1.236702
H -2.030309 -3.223829 0.051437
H -4.415935 0.144497 2.418790
H -5.271940 -0.159751 1.182137
H -3.742603 -2.091562 1.374260
H -3.629336 -3.564258 1.855302
O 1.092031 4.005619 1.501533
H 1.530411 4.706811 1.003638
H 1.375629 4.113427 2.417854
O -3.852540 2.409295 -2.192988
H -3.915620 2.094568 -3.103523
H -4.018776 3.359327 -2.232455

VIIIe

E= -4170.235750
O -0.006360 1.635829 0.243834
Zn 1.139064 -0.000264 0.020906
Zn 3.672789 -0.360020 -0.353246
O -0.393479 -1.373268 0.183252
O -2.610226 1.669331 0.357668
O 1.193312 4.028781 0.812911
O 0.091812 -4.054346 -0.110473
O -3.003826 -1.069308 1.032989
H -1.008562 1.688399 0.256235
H 0.378861 2.516199 0.441608
H -1.322894 -1.260658 0.488839
H -0.213682 -2.333652 0.084861
H 0.252145 -4.470297 -0.966823
H 0.556572 -4.598322 0.537746
H 1.380232 4.731176 0.178030
H 1.162818 4.455031 1.678359
H -3.153710 -1.150536 1.983357
H -3.683925 -1.640071 0.609198
H -2.964321 0.777583 0.518842
H -3.212936 2.117261 -0.267024
O -4.422974 2.950724 -1.347100
H -4.312972 3.146341 -2.285691
H -4.994202 3.647831 -1.001286
O -4.911142 -2.645241 -0.134940
H -4.928645 -3.608865 -0.180831
H -5.787301 -2.348889 -0.410339

VIIIf

E= -4170.223745
O 0.291665 0.000026 -0.782556
Zn 2.134254 0.000143 -0.196810
Zn 4.638627 0.000065 0.417160
O -1.363951 1.846443 -0.124229
O -1.364120 -1.846500 -0.124920
O -2.329450 3.923720 -1.743452
O -3.260778 0.000037 0.780950
O -5.756509 0.001132 -0.485734
H -0.304722 0.804063 -0.591675
H -0.304701 -0.804099 -0.592042
H -1.700176 2.549262 -0.713242
H -2.138129 1.337089 0.198353
H -2.138296 -1.337132 0.197628
H -1.700337 -2.549400 -0.713823
H -3.406875 -0.000515 1.747759
H -4.150582 0.000310 0.374581
H -6.337129 0.769935 -0.541436
H -6.336995 -0.767591 -0.543873
H -2.350015 3.956761 -2.707616
H -2.274184 4.842238 -1.452269
O -2.329362 -3.924819 -1.743313
H -2.349686 -3.959462 -2.707427
H -2.274192 -4.842865 -1.450618
O -3.646184 -0.000570 3.550990
H -3.676954 -0.770081 4.131961
H -3.673943 0.769511 4.131360

[2]VIIIa

E= -4170.234175
O -4.021315 0.001344 -0.595040
O -6.137382 0.000100 1.136829
O -1.661093 -1.416490 -0.172329
Zn -0.142180 -0.000953 0.134863
Zn 2.260267 0.002209 -0.617829
O 3.769881 0.000729 0.983615
O -1.660722 1.416886 -0.163550
O -1.498728 4.148306 -0.214938
O -1.504079 -4.148553 -0.214151
H -4.411787 0.002724 -1.477797
H -4.780549 0.000911 0.030279
H -2.588601 -1.144590 -0.320137
H -1.592811 -2.394944 -0.177086
H -1.590575 2.395200 -0.172473
H -2.587828 1.146003 -0.315491
H -6.615666 -0.771064 1.464782
H -6.612975 0.771186 1.468856
H -1.386053 -4.716558 0.557019
H -1.195764 -4.660773 -0.971802
H 3.609446 -0.008020 1.935413
H 4.745022 -0.001706 0.845829
H -1.381253 4.717532 0.555421
H -1.186450 4.657969 -0.972690
O 6.474292 -0.005374 0.655164
H 6.971263 -0.774178 0.349148
H 6.975143 0.767029 0.364932

[2]VIIIb

E= -4170.233506
O 3.154215 -0.016025 -0.030620
O 4.895470 -0.069961 -2.191706
O 0.821375 -1.410793 -0.162534
Zn -0.686039 0.010630 -0.230890
Zn -3.064717 0.040388 0.638799
O -4.647000 -0.096117 -1.129516
O 0.843891 1.409786 -0.219308
O 0.687200 4.131158 0.062294
O 0.624481 -4.127767 0.130409
H 3.692942 -0.004905 0.787268
H 3.784563 -0.034744 -0.779683
H 1.759949 -1.122573 -0.097988
H 0.747359 -2.382589 -0.056959
H 0.784042 2.382838 -0.116766
H 1.776747 1.107361 -0.137772
H 5.296146 -0.849611 -2.594656
H 5.285589 0.689397 -2.641087
H 0.461203 -4.742789 -0.595196
H 0.321578 -4.574911 0.930260
H -4.549805 -0.026753 -2.089145
H -5.595177 -0.058288 -0.945860
H 0.549633 4.747865 -0.667171
H 0.389299 4.589889 0.857425
O 4.596266 0.018328 2.341010
H 5.025348 0.789084 2.732014
H 4.986104 -0.748678 2.777802

[2]VIIIc

E= -4170.233413
O 4.074293 -0.005138 0.501635
Zn 2.033958 -0.011727 1.343325
Zn 0.084300 0.002722 -0.246907
O -1.439447 1.422423 -0.523965
O -1.437797 -1.416300 -0.539941
O -1.344281 -4.133984 -0.213392
O -1.346385 4.139400 -0.193517
O 4.985964 0.020134 -2.080987
O -3.799514 0.004376 -0.949215
H 4.843061 -0.010809 1.084847
H 4.404376 0.004013 -0.425537
H -2.366754 -1.141738 -0.673248
H -1.390070 -2.388802 -0.421830
H -2.367788 1.148359 -0.662600
H -1.391685 2.394790 -0.404656
H -4.563722 0.001278 -0.330099
H -4.182596 0.007931 -1.835205
H -1.187868 -4.584752 0.625499
H -1.090517 -4.759725 -0.902906
H -1.193861 4.588848 0.646798
H -1.088203 4.765727 -0.880858
H 5.361547 -0.747132 -2.530101
H 5.367197 0.794040 -2.513643
O -5.928618 -0.004532 0.767098
H -6.407383 -0.777557 1.089963
H -6.407198 0.764647 1.099283

[2]VIIId

E= -4170.232190
O 4.033039 2.016520 0.127019
O 1.211670 2.416299 -0.097620
Zn 0.288744 0.550535 0.296263
Zn -1.857120 -0.376277 -0.637908
O -3.513394 -0.569945 0.802658
O 2.223288 -0.222465 0.353061
O 3.015832 -2.678633 0.932837
O 0.050652 4.862820 -0.433649
O 3.607871 -4.655340 -0.903456
H 4.609975 2.096318 -0.644522

H 4.556195 2.341045 0.872267
H 2.175665 2.540602 -0.051067
H 0.776125 3.289763 -0.205939
H 2.496926 -1.152260 0.569515
H 3.010474 0.346909 0.331533
H 3.205602 -3.387882 0.284758
H 2.876508 -3.109848 1.782911
H -0.360572 5.402773 0.252330
H -0.289823 5.193042 -1.274133
H 2.997983 -5.185752 -1.430061
H 4.481612 -5.041973 -1.037503
H -3.453733 -0.478858 1.761828
H -4.397082 -0.952511 0.596318
O -5.978765 -1.609886 0.285098
H -6.702560 -1.086789 -0.081273
H -6.133540 -2.517311 -0.005209

[2]VIIIe

E= -4170.231973
O 0.870401 0.886024 -1.195399
Zn -0.659589 0.014368 0.078185
Zn -3.143054 -0.565566 -0.049224
O 0.545458 -1.869441 -0.024093
O 0.495968 0.821790 1.790457
O 3.246450 0.304617 1.881707
O 3.349442 -1.793287 0.095001
O 3.644866 -0.130141 -2.015170
O 3.306274 2.035105 -0.309163
H 0.125967 -2.732669 0.073727
H 1.515576 -1.992126 0.045639
H 1.325352 0.324691 -1.841811
H 1.566394 1.472905 -0.836537
H 1.432898 0.599649 1.980792
H 0.041149 0.918172 2.636799
H 3.789232 0.274508 2.679605
H 3.406436 -0.536524 1.396191
H 3.907128 -2.580302 0.137922
H 3.582068 -1.323294 -0.743776
H 3.576849 2.960155 -0.258312
H 3.435014 1.646932 0.580571
H 4.202691 -0.185203 -2.800719
H 3.801239 0.746062 -1.610225
O -4.456692 1.419627 -0.353789
H -4.235767 2.247054 -0.803546
H -5.410276 1.301588 -0.461541

[2]VIIIf

E= -4170.230845
O -2.901363 0.864339 -0.328080
Zn -0.867584 0.900751 -0.279297
Zn 0.867693 -0.903271 -0.279599
O 2.901486 -0.863985 -0.327543
O 4.452788 -3.117704 -0.458294
O -4.449119 3.120434 -0.459320
H -3.465732 1.665969 -0.372044
H -3.473797 0.050255 -0.301502
H 3.472830 -0.049134 -0.301032
H 3.466926 -1.664865 -0.371468
H -4.650236 3.714366 0.274015
H -4.667611 3.605429 -1.264391
H 4.653384 -3.711506 0.275292
H 4.670525 -3.603413 -1.263139
O -4.325243 -1.359578 -0.266429
H -4.916782 -1.630351 0.465851
H -4.674507 -1.767428 -1.066487
O 4.322455 1.361825 -0.265974
H 4.914532 1.632562 0.465873
H 4.670801 1.770161 -1.066179
O 5.942949 2.136567 1.835927
H 6.851237 1.864939 2.015095
H 5.794873 2.931385 2.362570
O -5.944138 -2.134460 1.836614
H -6.852281 -1.862722 2.016374
H -5.795953 -2.929544 2.362832

[2]VIIIg

E= -4170.230630
O -2.229342 -0.043465 -0.074069
Zn -0.274612 -0.481124 -0.177335
Zn 1.925240 0.631019 0.270341
O 3.805806 -0.194288 0.012840
O -3.112130 2.308290 0.767658
O 4.281609 -2.710586 -0.928461
O 6.142554 1.109377 0.556807
O -4.076842 -1.821253 -0.747276
O -4.097687 4.340429 -0.832677
H -2.954875 -0.678139 -0.308816
H -2.608396 0.823121 0.229107
H 4.011233 -1.095803 -0.323249
H 4.645828 0.276897 0.207967
H -4.515377 -1.843360 -1.604599
H -4.602476 -2.388383 -0.147016
H 6.543507 1.785514 -0.003076
H 6.504484 1.246196 1.440949
H -3.481722 3.020604 0.206892
H -3.450821 2.455045 1.657527
H 4.603305 -2.899662 -1.818599
H 4.582169 -3.443341 -0.376825
H -4.985853 4.442805 -1.195127
H -3.661314 5.192336 -0.954047
O -5.515475 -3.453700 0.963915

H -6.353332 -3.263197 1.402614
H -5.383159 -4.405894 1.046210

²VIIIh

E= -4170.230354
O 4.012213 0.053735 -0.229836
Zn 2.102002 0.761096 0.110588
Zn 0.002479 -0.601144 0.255646
O -1.947979 -0.301048 0.671104
O -3.788134 -2.172361 0.463751
O 6.239503 1.624641 -0.312816
O 4.631731 -2.579426 -0.548224
O -3.316946 2.030758 0.466403
O -5.966319 1.818345 -0.729800
H 4.815633 0.619244 -0.259333
H 4.273207 -0.887859 -0.347293
H -2.420383 0.564731 0.618214
H -2.632151 -1.028324 0.628195
H 6.526868 2.147044 -1.071629
H 6.694732 1.993515 0.453991
H -4.597610 -1.855392 0.004301
H -4.088960 -2.684068 1.223526
H 4.854703 -2.985455 -1.394952
H 5.049469 -3.127517 0.127515
H -3.294312 2.699200 1.161203
H -4.232296 2.010072 0.134069
H -6.736293 2.112513 -0.223627
H -5.997597 2.324076 -1.553640
O -5.976782 -1.106985 -0.785974
H -6.021172 -0.142553 -0.901155
H -6.426586 -1.499043 -1.543263

²VIIIi

E= -4170.228741
O -2.457023 1.381155 -0.604492
Zn -0.889400 0.011278 -0.012668
O -1.849235 0.094082 1.983414
Zn 1.589548 -0.027778 0.441708
O 2.964818 -0.128575 -1.243058
O -2.463539 -1.470533 -0.290740
O 0.212412 0.207372 3.700649
O -2.120763 3.761652 -1.877829
H -1.177529 0.107975 2.710785
H -2.406985 0.873747 2.105558
H -2.802226 -1.710075 0.583403
H -2.365879 -2.304905 -0.804685
H -3.187762 0.914063 -1.031866
H -2.318677 2.222845 -1.096950
H -2.520958 4.584340 -1.570303
H -1.767096 3.946904 -2.756203
H 1.013085 0.121886 3.152185
H 0.404415 -0.235291 4.536744
H 2.740098 -0.067150 -2.179659
H 3.946704 -0.083400 -1.168720
O -2.255125 -3.749908 -1.770454
H -2.984504 -4.113449 -2.287706
H -1.617922 -4.467201 -1.666768
O 5.674441 -0.005842 -1.108725
H 6.160565 0.761429 -0.781913
H 6.232331 -0.771936 -0.925235

Xa

E= -4323.114580
O 1.784812 1.645241 2.171535
O 1.716684 3.409546 0.082812
O -0.010959 -0.347294 1.372758
Zn -1.470857 -0.060160 -0.141366
O -0.418449 -1.350707 -1.588865
O -0.389190 1.596256 -1.017676
O 1.691043 -2.545532 1.625986
O 2.008347 -2.659555 -1.150246
O 3.782935 -0.570182 -1.108232
O 3.635621 -0.410595 1.498393
O 2.283978 1.594611 -1.956864
H 0.469711 1.421336 -1.455219
H -0.256645 2.433349 -0.546444
H -0.954043 -1.814667 -2.244463
H 0.407117 -1.870549 -1.462880
H 2.257161 -3.419461 -1.691688
H 2.724379 -1.987404 -1.263697
H 0.474561 -1.182221 1.537864
H 0.555114 0.386675 1.694737
H 4.703777 -0.646423 -1.389724
H 3.803614 -0.468820 -0.105751
H 1.805833 -2.858458 0.705467
H 1.583529 -3.326702 2.182718
H 3.137682 0.359941 1.833270
H 3.128257 -1.188599 1.790352
H 1.802956 2.386374 1.521608
H 1.735211 2.047453 3.047755
H 2.135481 2.964490 -0.682003
H 2.021381 4.325202 0.077501
H 2.876856 0.837948 -1.737851
H 2.443705 1.804584 -2.885459
Zn -4.046950 -0.043119 0.195935

Xb

E= -4323.113466
O 0.322590 -1.382811 -1.037407

Zn 1.587390 0.071037 -0.062460
O 0.427396 1.837426 -0.499608
O 0.472069 -0.157221 1.747486
O -2.254708 2.091424 -0.246364
O -3.412319 0.027687 -1.514055
O -2.181063 -2.154414 -0.330398
O -1.875668 1.227522 2.283757
O -2.819452 -4.767409 -0.970012
O -3.286164 4.576610 -0.905442
O -2.096505 -1.547986 2.363169
H 0.808755 2.522598 -1.060990
H -0.558208 1.956390 -0.497725
H 0.536452 -1.706658 -1.920375
H -0.596320 -1.683921 -0.815003
H -0.226004 0.493225 1.981163
H 0.100420 -1.014104 2.007905
H -2.005034 1.890033 2.972859
H -2.128858 1.649142 1.423628
H -2.646951 2.957595 -0.483693
H -2.758296 1.398490 -0.744846
H -2.415843 -3.082017 -0.543974
H -2.252012 -2.059711 0.644855
H -4.344657 -0.086485 -1.728922
H -3.096742 -0.825743 -1.145910
H -2.534683 -2.009516 3.088067
H -2.267937 -0.592563 2.490320
H -3.245099 4.985618 -1.778602
H -3.913741 5.105589 -0.397986
H -2.246291 -5.539421 -0.888419
H -3.645851 -5.091618 -1.348213
Zn 4.181527 0.019845 -0.216978

Xc

E= -4323.113197
O 0.600714 0.519070 1.910973
Zn 1.612795 -0.061125 0.067688
O 0.177178 0.988208 -1.090562
O 0.523424 -1.886111 -0.304138
O -2.166018 -2.180831 -0.242363
O -3.098315 -1.228874 2.103955
O -2.260543 -0.054194 -1.935595
O -2.017115 1.365516 2.152093
O -2.237797 2.303566 -0.342642
O -3.285703 4.753808 -1.033257
O -3.052414 -4.729170 -0.879628
H 0.976539 -2.729732 -0.189078
H -0.452141 -2.052117 -0.220570
H 1.061370 0.494263 2.757996
H -0.323021 0.823236 2.074151
H -0.504726 0.480919 -1.579896
H -0.328960 1.735125 -0.721347
H -2.583590 -0.234180 -2.826326
H -2.360398 -0.890928 -1.419557
H -2.506255 -3.074596 -0.455960
H -2.577092 -1.914260 0.616633
H -2.235760 2.015750 2.831125
H -2.194017 1.801117 1.276238
H -3.974501 -1.335305 2.490926
H -2.841939 -0.294134 2.233899
H -2.639029 3.158115 -0.608231
H -2.560966 1.621033 -0.959894
H -2.805559 5.436815 -1.517268
H -4.190900 5.076510 -0.947248
H -2.894578 -5.524931 -0.356932
H -3.710262 -4.976597 -1.540797
Zn 4.193155 0.113658 -0.189306

Xd

E= -4323.113085
O -0.521185 0.833692 -1.367973
Zn -1.684079 -0.155350 0.116254
O -0.558484 -1.964329 -0.296847
O -0.348881 0.626037 1.624803
O 2.290228 0.251547 1.253616
O 2.127206 -1.811733 -0.589442
O 1.896354 2.005260 -0.880974
O 2.080286 -0.043701 -2.686881
O 2.420545 4.715573 -0.799391
O 3.636075 -4.130151 -0.705196
O 4.219974 0.245330 3.244424
H -0.888020 -2.834748 -0.044341
H 0.429199 -2.015383 -0.377390
H -0.209366 0.309878 -2.121495
H 0.252576 1.395284 -1.119578
H 0.630678 0.464229 1.613179
H -0.638862 0.637711 2.544695
H 2.968532 0.239044 1.959823
H 2.397296 -0.568687 0.723741
H 2.679840 -2.618667 -0.647605
H 2.242387 -1.318496 -1.432796
H 2.093629 2.964944 -0.853159
H 2.201141 1.608526 -0.039866
H 2.555736 -0.008982 -3.524878
H 2.234053 0.811130 -2.229782
H 4.528778 -4.260620 -0.363111
H 3.444476 -4.908039 -1.243174
H 1.731112 5.374009 -0.648171
H 3.112023 5.170211 -1.295919
H 4.131730 -0.108295 4.137940
H 4.976401 0.843926 3.273193
Zn -4.276264 -0.077888 0.261065

Xe

E= -4323.112317
O 3.795385 -2.447251 -1.821390
O 1.500581 -1.895377 -0.412930
Zn 0.839227 0.097424 -0.051669
O -0.741880 -0.210881 -1.469885
O -0.404641 -0.466059 1.575873
O 0.617338 -0.679352 4.129771
O -0.964265 -2.978420 -1.227600
O -2.380227 -2.422236 1.038371
O -4.342263 -0.903426 0.043666
O -3.196420 1.192593 -1.425127
O -3.058800 3.739003 -0.440866
H -1.573612 0.306217 -1.453589
H -0.974633 -1.151973 -1.600527
H 0.808906 -2.494253 -0.755397
H 2.323366 -2.065521 -0.920262
H 4.147362 -1.963421 -2.578519
H 4.536323 -2.948184 -1.458818
H -3.757169 4.227469 0.011707
H -2.373726 4.385791 -0.650032
H -1.071505 -1.177719 1.485567
H -0.020664 -0.532053 2.475769
H -3.557427 1.293144 -2.315409
H -3.149796 2.105988 -1.060251
H 1.454068 -1.103865 4.356123
H 0.454696 -0.030556 4.825629
H -3.179887 -1.904105 0.762840
H -2.667656 -3.029165 1.731076
H -5.252191 -0.760480 0.326044
H -4.053029 -0.093478 -0.423792
H -1.539014 -3.008883 -0.429672
H -1.233140 -3.704326 -1.802746
Zn 2.296782 2.243264 0.036841

Xf

E= -4323.110633
O 2.614600 -3.344855 -2.012059
O 0.650997 -1.848004 -0.802826
Zn 1.054556 0.094620 -0.042127
O -0.660685 0.158768 1.207028
O -0.077711 0.933232 -1.684606
O -0.443917 -0.671918 3.843268
O -1.724273 -1.244346 -2.130605
O -3.103852 -0.477238 0.041087
O -3.899429 2.009466 -0.765006
O -4.975193 -1.995773 1.413439
O -1.443383 3.463839 -1.234843
H -0.533182 1.785671 -1.559748
H -0.731259 0.298495 -2.047582
H -0.144037 -1.901566 -1.369898
H 1.364857 -2.365875 -1.233213
H 3.315051 -3.026464 -2.594467
H 2.905024 -4.208768 -1.694924
H -1.544181 -0.126458 0.879880
H -0.575313 -0.138165 2.137110
H -1.380147 4.077913 -1.979448
H -1.065920 3.938450 -0.481359
H 0.054140 -1.458102 4.099927
H -0.338622 -0.047903 4.572346
H -3.518926 0.390214 -0.164343
H -3.768653 -1.007783 0.525983
H -4.701825 2.526754 -0.633511
H -3.173550 2.643142 -0.899621
H -2.381461 -1.093163 -1.405853
H -2.203691 -1.593468 -2.890607
H -5.063567 -2.073129 2.370990
H -5.664353 -2.557079 1.038171
Zn 3.400524 0.907778 0.719832

XIIa

E= -4475.987397
O 0.099646 3.390181 2.176421
O 1.921137 1.502214 1.502016
O 0.216026 -0.536049 0.881588
Zn -1.493217 -0.415514 -0.339215
O -0.474162 1.006121 -1.558921
O -0.618613 -2.050024 -1.534960
O 1.337880 -2.698701 2.222906
O 1.586340 -3.450410 -0.485838
O 3.509266 -1.497654 -0.797442
O 3.508320 -0.819096 1.734740
O 2.286870 0.506326 -2.266159
O 2.682700 2.861633 -0.912890
O 0.026478 3.661559 -0.704795
H -0.028061 4.119651 2.793348
H -0.120810 3.722472 1.288489
H 0.428904 0.753515 -1.835847
H -0.427403 1.944707 -1.278990
H -1.211853 -2.637932 -2.019112
H 0.106554 -2.603889 -1.168869
H 1.819337 -4.316553 -0.843469
H 2.345042 -2.852334 -0.689827
H 0.422588 -1.245462 1.520512
H 0.789697 0.240799 1.090820
H 4.416292 -1.716803 -1.047052
H 3.556565 -1.204187 0.167791
H 1.396411 -3.234213 1.406773
H 1.079645 -3.293367 2.937638

H 3.080570 0.046508 1.884842
H 2.937342 -1.467972 2.181814
H 2.306289 1.991200 0.750574
H 1.375733 2.174446 1.970295
H 2.701394 2.096406 -1.530009
H 3.503369 3.352150 -1.043826
H 0.993411 3.626944 -0.863608
H -0.314422 4.390919 -1.237949
H 2.733025 -0.238280 -1.794603
H 2.417415 0.345589 -3.209176
Zn -4.005521 -0.217923 0.289752

XVIa

E= -4781.730926
O -1.852148 1.281599 -2.941764
O 0.664613 0.642518 -2.724148
Zn 1.612316 -0.320623 -1.096950
O 2.457557 1.522951 -0.545606
O -0.056612 -0.006287 0.367107
O 0.610413 -1.264564 2.533651
O 3.568059 1.785569 1.897721
O -1.467765 -2.789160 3.435689
O -0.151081 2.379823 1.756644
O -2.789841 2.666165 1.278617
O 1.483322 1.248790 3.715039
O -2.041014 3.613830 -1.243966
O 0.696265 3.858686 -0.466210
O -2.929861 -0.026012 0.427970
O -3.779787 -0.625651 -2.067520
O -3.598016 -2.449970 1.596946
O -3.608294 -3.238752 -0.974329
H 0.851063 1.806087 3.212265
H 1.479321 1.573492 4.623611
H 3.005449 1.527784 2.655117
H 4.471482 1.529825 2.115657
H -3.276639 -3.996438 -1.468422
H -3.617973 -2.472437 -1.580454
H -0.315003 0.839233 -2.818341
H 1.014476 0.485122 -3.609166
H -0.076678 -1.835486 2.945962
H 0.887655 -0.604400 3.191147
H -3.067385 0.837480 0.863839
H -3.228378 -0.738090 1.033815
H -1.861010 2.638704 1.627792
H -3.324152 3.128596 1.935894
H -2.455597 3.399445 -0.379007
H -2.545752 4.350534 -1.611136
H -0.182394 3.884711 -0.898431
H 1.100559 4.722152 -0.616233
H -4.699618 -0.426271 -2.283280
H -3.631266 -0.326256 -1.137934
H -2.553555 0.625529 -2.763916
H -2.047304 2.067608 -2.400639
H -4.466272 -2.646412 1.970248
H -3.587743 -2.875221 0.703312
H -1.359563 -3.693252 3.752433
H -2.213802 -2.803033 2.804683
H 0.167005 -0.565449 1.182092
H -1.009453 -0.153807 0.194487
H 1.923804 2.326676 -0.684142
H 2.913354 1.614702 0.327512
H -0.064971 1.550601 1.224416
H 0.268770 3.060146 1.192152
Zn 2.849520 -2.600316 -1.025453

XVIIa

E= -4858.167744
O -1.075236 1.890903 -3.039846
O 0.713934 0.012904 -2.753120
Zn 1.333484 -1.068422 -1.047562
O 0.129261 0.086847 0.422906
O 2.952646 0.265745 -0.825990
O 0.423133 -0.961557 2.785396
O 4.380572 0.312596 1.461169
O -1.915185 -0.814994 4.196326
O 1.222277 2.423533 1.417941
O -1.091854 3.742069 0.979479
O 2.486918 0.977532 3.429680
O 2.460647 3.163946 -0.969364
O -0.106676 4.192435 -1.599965
O -3.908791 -0.221986 2.296521
O -4.544174 -2.205771 0.679654
O -4.050794 -1.622609 -1.930329
O -2.466507 1.276401 0.488083
O -3.586520 1.167979 -1.972907
H 2.071917 1.675873 2.877736
H 2.705795 1.383486 4.277117
H 3.855122 0.434477 2.277154
H 5.099091 -0.293542 1.674236
H -4.338744 -3.131085 0.853454
H -4.397020 -2.058774 -0.284873
H 0.001470 0.713419 -2.848832
H -4.559019 -1.971911 -2.671207
H -3.939824 -0.664826 -2.094189
H 0.835930 -0.394857 -3.618457
H -0.376127 -0.964700 3.359356
H 1.099329 -0.433835 3.244722
H -2.170308 2.154407 0.796663
H -2.991246 0.856830 1.209132
H -0.236917 3.356802 1.305024

H -1.346264 4.426585 1.610642
H -0.540231 4.204805 -0.717700
H -0.264177 5.059502 -1.994147
H 1.671159 3.591891 -1.361375
H 3.215958 3.719972 -1.197077
H -4.367199 1.725720 -2.076647
H -3.288210 1.268714 -1.034958
H -1.996544 1.696569 -2.778648
H -0.830021 2.730303 -2.611235
H -4.744221 0.108446 2.649458
H -4.151336 -1.003437 1.721797
H -2.192302 -1.464449 4.852612
H -2.682909 -0.669897 3.606994
H 0.177561 -0.380613 1.319868
H -0.811181 0.334801 0.296544
H 2.822970 1.200836 -1.065584
H 3.514236 0.244853 -0.012038
H 0.891793 1.582944 1.016034
H 1.835803 2.780218 0.742595
Zn 1.474398 -3.641821 -0.747413

XVIIIa

E= -4934.602492
O 0.417950 -1.127054 3.665935
O -1.512415 0.312728 2.693518
Zn -1.938135 0.501491 0.630849
O -1.525180 2.534398 0.862928
O 0.195489 0.093399 -0.138148
O 0.183382 -0.023458 -2.799231
O -1.635671 4.179802 -1.248617
O 2.838963 -0.438095 -3.095490
O 1.734642 2.362991 -0.570044
O 4.148866 1.246759 -1.025926
O 4.219299 -0.224509 1.250030
O 2.519188 0.853542 3.189869
O 1.089900 3.071817 2.061933
O 0.243723 2.949619 -2.897875
O 0.594110 -3.442356 2.055392
O 2.121524 -1.956700 0.415963
O 2.435321 -3.033165 -2.059638
O -0.153261 -2.921132 -2.454446
O -1.411033 -3.460293 -0.018422
H 0.891700 2.895889 -2.163426
H 0.681542 3.423760 -3.615702
H -1.115423 3.833318 -2.001752
H -2.442293 4.565552 -1.608526
H -0.384340 -2.021257 -2.736300
H -0.667274 -3.108721 -1.640812
H -0.806857 -0.287094 3.084608
H -2.082463 -4.150208 0.038122
H -0.835053 -3.571740 0.762477
H -2.257728 0.325474 3.305918
H 1.151571 -0.120691 -2.996465
H -0.054270 0.858950 -3.129060
H 2.924861 -1.485355 0.710082
H 2.344127 -2.393838 -0.442113
H 4.336382 0.333201 0.440032
H 5.095908 -0.556373 1.480772
H 3.201102 0.533552 2.557878
H 2.989031 1.028645 4.015424
H 1.537812 2.345959 2.546225
H 1.180796 3.858985 2.612950
H 0.953429 -4.275301 2.384637
H 1.281295 -3.043368 1.468451
H 0.529478 -1.992317 3.223876
H 1.230571 -0.611044 3.522879
H 2.830277 -3.870203 -2.330259
H 1.455608 -3.085884 -2.275339
H 3.329133 1.786617 -0.884283
H 4.836520 1.852680 -1.328494
H 3.413423 0.046706 -2.479990
H 2.917594 -1.381000 -2.853603
H 0.111481 -0.019046 -1.129291
H 0.754583 -0.661464 0.150252
H -0.729851 2.763943 1.378308
H -1.603052 3.168917 0.106393
H 1.200358 1.557411 -0.368783
H 1.632570 2.897294 0.242168
Zn -3.790896 -0.810435 -0.602486

XIXa

E= -5011.039917
O 1.020000 -3.904263 -0.310018
O 2.430777 -1.971410 -1.328781
Zn 2.169419 0.033916 -0.725801
O 0.014523 -0.172967 -0.068770
O 1.504100 0.504999 -2.652181
O -0.725240 2.099624 1.106053
O 0.399440 2.956854 -2.900565
O -3.463309 1.560058 1.166443
O -3.473077 4.064100 -0.086472
O -0.825973 4.261090 -0.677795
O -2.723516 -0.096633 3.304094
O -0.341665 0.905462 3.685717
O -1.614438 -1.929697 1.565755
O 0.430434 -3.445220 2.424261
O 1.633334 -1.037763 3.415175
O -3.429649 -2.789218 -0.433276
O -4.190266 -0.244598 -0.937042
O -1.924201 0.237886 -2.109696

O -0.378370 -1.463010 -3.721830
O -1.283365 -3.632591 -2.044068
H -1.804512 4.317806 -0.550123
H -0.477092 5.129495 -0.442316
H -0.073773 3.435696 -2.191346
H 0.740944 3.626078 -3.503649
H -0.219285 1.502567 2.927356
H 0.389407 0.252524 3.643150
H 1.927470 -2.727889 -0.898159
H 2.358410 -1.129794 4.043715
H 1.364273 -1.942937 3.166488
H 3.334819 -2.274324 -1.476784
H -1.700573 1.974051 1.094787
H -0.573027 2.905251 0.570084
H -2.293663 -2.293025 0.963493
H -2.091108 -1.360608 2.214100
H -3.851726 -1.919259 -0.654165
H -4.151075 -3.396547 -0.227773
H -2.097909 -3.362470 -1.565158
H -1.504226 -4.462077 -2.486493
H -0.590684 -2.278269 -3.223294
H -0.299969 -1.722815 -4.647636
H 0.238501 -4.171755 3.029602
H -0.426891 -2.989891 2.238576
H 0.830637 -3.866027 0.648488
H 0.167821 -3.915344 -0.781517
H -3.193846 -0.281869 4.125543
H -1.831539 0.295401 3.562044
H -3.384562 -0.023315 -1.496267
H -4.966310 0.031709 -1.439940
H -3.791056 0.931299 0.492431
H -3.432679 1.059896 2.008063
H -0.190142 0.685545 0.397061
H -0.368423 -0.865426 0.509357
H 0.941890 -0.170075 -3.077023
H 1.085798 1.390945 -2.786831
H -1.219367 0.116464 -1.441256
H -1.591979 -0.230490 -2.895562
H -3.647884 3.299143 0.496064
H -4.132491 4.734115 0.124546
Zn 3.698898 1.381904 0.870945

Infrared Multiple Photon Dissociation Spectra of $Zn_2^+(H_2O)_n$ (n = 1–20)

The following IRMPD spectra are given in ASCII format, presented in wavenumber (cm^{-1}) and calculated single-photon cross sections (10^{-21} cm^2) for cluster sizes n = 1–8, 10, 16 and 20.

$Zn_2^+(H_2O)$

Wavenumber / cm^{-1}	Calculated Cross Section / 10^{-21} cm^2
2240.143	0.00
2289.902	0.00
2339.729	0.00
2390.057	0.01
2440.215	0.00
2490.04	0.00
2540.005	0.00
2590.003	0.00
2639.916	0.00
2690.342	0.00
2739.726	0.00
2790.179	0.00
2800.336	0.00
2809.778	0.00
2820.079	0.00
2829.655	0.00
2840.102	0.00
2849.815	0.00
2859.594	0.00
2870.264	0.00
2880.184	0.00
2890.173	0.00
2900.232	0.00
2910.361	0.00
2919.708	0.00
2929.974	0.00
2940.312	0.00
2949.853	0.00
2960.332	0.00
2970.003	0.00
2979.738	0.00
2990.431	0.00
3000.3	0.00
3010.235	0.00
3020.236	0.00

3030.303	0.00
3040.438	0.00
3049.71	0.00
3059.976	0.00
3070.31	0.00
3079.766	0.00
3090.235	0.00
3099.814	0.00
3110.42	0.00
3120.125	0.00
3129.89	0.00
3139.717	0.00
3149.606	0.00
3159.558	0.00
3169.572	0.00
3179.65	0.00
3189.793	0.00
3200	0.00
3210.273	0.00
3219.575	0.00
3229.974	0.00
3240.441	0.00
3249.919	0.00
3260.515	0.00
3270.111	0.00
3279.764	0.00
3289.474	0.00
3300.33	0.00
3300.33	0.00
3310.162	0.00
3320.053	0.00
3330.003	0.00
3340.013	0.00
3350.084	0.00
3360.215	0.00
3370.408	0.00
3379.52	0.00
3389.831	0.00
3400.204	0.00
3409.478	0.00
3419.973	0.00
3430.532	0.00
3439.972	0.00
3449.465	0.00
3460.208	0.00

3469.813	0.00
3479.471	0.00
3490.401	0.00
3500.175	0.00
3510.004	0.00
3519.887	0.00
3529.827	0.00
3539.823	0.03
3549.876	0.09
3559.986	0.13
3570.154	0.26
3580.38	0.59
3589.375	0.46
3599.712	0.27
3610.108	0.14
3620.565	0.10
3629.764	0.07
3640.335	0.05
3649.635	0.06
3660.322	0.07
3669.725	0.05
3680.53	0.05
3690.037	0.04
3699.593	0.04
3710.575	0.07
3720.238	0.06
3729.952	0.04
3739.716	0.04
3749.531	0.02
3759.399	0.04
3769.318	0.03
3779.289	0.02
3789.314	0.00
3799.392	0.00
3809.524	0.03
3819.71	0.00
3829.95	0.00
3840.246	0.00
3850.597	0.00
3859.514	0.00
3869.969	0.00
3880.481	0.00
3889.537	0.00
3900.156	0.00
3909.304	0.00

3920.031	0.00
3929.273	0.00
3940.11	0.00
3949.447	0.00
3960.396	0.00
3969.829	0.00
3979.308	0.00
3990.423	0.00
4000	0.00

$Zn_2^+(H_2O)_2$

Wavenumber / cm^{-1}	Calculated Cross Section / 10^{-21} cm^2
2240.143	0.01
2289.902	0.01
2339.729	0.01
2390.057	0.01
2440.215	0.01
2490.04	0.01
2540.005	0.01
2590.003	0.01
2639.916	0.01
2690.342	0.01
2739.726	0.01
2790.179	0.01
2840.102	0.01
2890.173	0.09
2940.312	0.33
2990.431	0.27
3040.438	0.15
3090.235	0.07
3139.717	0.06
3189.793	0.12
3240.441	0.06
3249.919	0.05
3260.515	0.05
3270.111	0.04
3279.764	0.01
3289.474	0.01
3300.33	0.01
3310.162	0.01
3320.053	0.01
3330.003	0.01
3340.013	0.01
3350.084	0.01

3360.215	0.01
3370.408	0.01
3379.52	0.01
3389.831	0.01
3400.204	0.01
3409.478	0.01
3419.973	0.01
3430.532	0.01
3439.972	0.01
3449.465	0.01
3460.208	0.01
3469.813	0.01
3479.471	0.01
3490.401	0.04
3500.175	0.01
3510.004	0.01
3519.887	0.01
3529.827	0.05
3539.823	0.06
3549.876	0.09
3559.986	0.12
3570.154	0.23
3580.38	0.79
3589.375	2.69
3599.712	2.81
3610.108	1.25
3620.565	0.93
3629.764	0.85
3640.335	1.03
3649.635	0.92
3660.322	0.99
3669.725	0.94
3680.53	0.96
3690.037	0.93
3699.593	0.92
3710.575	0.81
3720.238	0.68
3729.952	0.61
3739.716	0.50
3749.531	0.37
3759.399	0.29
3769.318	0.24
3779.289	0.20
3789.314	0.15
3799.392	0.12

3809.524	0.10
3819.71	0.11
3829.95	0.10
3840.246	0.07
3850.597	0.06
3859.514	0.06
3869.969	0.01
3880.481	0.01
3889.537	0.01
3900.156	0.01
3909.304	0.01
3920.031	0.01
3929.273	0.01
3940.11	0.01
3949.447	0.01
3960.396	0.01
3969.829	0.01
3979.308	0.01
3990.423	0.01
4000	0.01

$Zn^{2+}(H_2O)_3$

**Wavenumber / cm^{-1}**	**Calculated Cross Section Zn loss / 10^{-21} cm^2**	**Calculated Cross Section Water loss / 10^{-21} cm^2**
2250.225	0.00	0.02
2259.887	0.02	0.02
2270.148	0.00	0.01
2279.982	0.02	0.02
2289.902	0.00	0.00
2299.908	0.01	0.00
2310.002	0.00	0.02
2320.186	0.00	0.02
2329.916	0.00	0.00
2339.729	0.00	0.00
2350.176	0.00	0.00
2360.16	0.00	0.00
2370.23	0.00	0.01
2379.819	0.00	0.00
2390.057	0.00	0.01
2399.808	0.00	0.00
2410.219	0.00	0.00
2420.136	0.00	0.01
2430.134	0.00	0.01
2440.215	0.00	0.00

2449.78	0.00	0.01
2460.025	0.00	0.00
2469.746	0.01	0.00
2480.159	0.00	0.01
2490.04	0.00	0.00
2500	0.01	0.01
2510.04	0.00	0.01
2520.161	0.00	0.01
2529.724	0.00	0.00
2540.005	0.00	0.00
2549.72	0.00	0.00
2560.164	0.00	0.01
2570.033	0.01	0.01
2579.979	0.00	0.00
2590.003	0.00	0.01
2600.104	0.00	0.00
2610.285	0.00	0.01
2619.859	0.00	0.01
2630.195	0.00	0.01
2639.916	0.00	0.01
2649.709	0.00	0.01
2660.282	0.00	0.01
2670.227	0.00	0.01
2680.247	0.01	0.01
2690.342	0.00	0.01
2699.784	0.01	0.02
2710.027	0.00	0.01
2720.348	0.01	0.00
2730.003	0.01	0.02
2739.726	0.00	0.02
2750.275	0.01	0.02
2760.144	0.01	0.02
2770.083	0.01	0.02
2780.095	0.02	0.02
2790.179	0.02	0.03
2800.336	0.02	0.03
2809.778	0.02	0.03
2820.079	0.03	0.04
2829.655	0.03	0.05
2840.102	0.04	0.05
2849.815	0.04	0.05
2859.594	0.05	0.06
2870.264	0.06	0.07
2880.184	0.07	0.09
2890.173	0.08	0.11

2900.232	0.08	0.12
2910.361	0.09	0.13
2919.708	0.10	0.14
2929.974	0.10	0.16
2940.312	0.11	0.19
2949.853	0.10	0.17
2960.332	0.11	0.19
2970.003	0.12	0.20
2979.738	0.12	0.22
2990.431	0.15	0.25
3000.3	0.17	0.29
3010.235	0.20	0.35
3020.236	0.24	0.42
3030.303	0.33	0.60
3040.438	0.42	0.80
3049.71	0.55	1.10
3059.976	0.72	1.58
3070.31	0.95	2.39
3079.766	1.13	3.08
3090.235	1.36	3.95
3099.814	1.48	4.17
3110.42	1.59	4.39
3120.125	1.61	4.29
3129.89	1.58	4.15
3139.717	1.44	3.75
3149.606	1.33	3.45
3159.558	1.17	3.21
3169.572	1.08	3.47
3179.65	0.90	2.87
3189.793	0.77	2.34
3200	0.67	1.90
3210.273	0.60	1.73
3219.575	0.56	1.61
3229.974	0.52	1.49
3240.441	0.52	1.39
3249.919	0.50	1.30
3260.515	0.56	1.45
3270.111	0.53	1.26
3279.764	0.49	1.16
3289.474	0.47	1.14
3300.33	0.45	1.07
3310.162	0.45	1.09
3320.053	0.46	1.05
3330.003	0.44	0.92
3340.013	0.43	0.93

3350.084	0.44	0.91
3360.215	0.42	0.87
3370.408	0.41	0.87
3379.52	0.41	0.86
3389.831	0.40	0.90
3400.204	0.43	0.95
3409.478	0.45	1.01
3419.973	0.51	1.06
3430.532	0.56	1.09
3439.972	0.62	1.10
3449.465	0.63	1.06
3460.208	0.63	1.02
3469.813	0.62	0.94
3479.471	0.62	0.97
3490.401	0.57	0.94
3500.175	0.52	0.81
3510.004	0.42	0.68
3519.887	0.33	0.51
3529.827	0.40	0.67
3539.823	0.40	0.71
3549.876	0.36	0.68
3559.986	0.31	0.65
3570.154	0.27	0.63
3580.38	0.25	0.69
3589.375	0.29	1.09
3599.712	0.44	1.69
3610.108	0.38	1.36
3620.565	0.55	1.77
3629.764	0.50	2.04
3640.335	0.70	2.90
3649.635	0.79	3.09
3660.322	1.20	4.18
3669.725	1.63	5.34
3680.53	1.76	6.54
3690.037	1.50	5.28
3699.593	1.50	4.87
3710.575	1.09	3.55
3720.238	0.77	2.82
3729.952	0.56	2.15
3739.716	0.39	1.60
3749.531	0.27	1.16
3759.399	0.21	0.96
3769.318	0.16	0.79
3779.289	0.13	0.73
3789.314	0.13	0.68

3799.392	0.11	0.66
3809.524	0.12	0.71
3819.71	0.11	0.67
3829.95	0.12	0.66
3840.246	0.10	0.59
3850.597	0.10	0.51
3859.514	0.09	0.47
3869.969	0.10	0.43
3880.481	0.10	0.40
3889.537	0.10	0.35
3900.156	0.09	0.31
3909.304	0.08	0.29
3920.031	0.08	0.25
3929.273	0.07	0.23
3940.11	0.07	0.21
3949.447	0.06	0.19
3960.396	0.06	0.18
3969.829	0.06	0.18
3979.308	0.06	0.17
3990.423	0.05	0.16
4000	0.00	0.00

$Zn^{2+}(H_2O)_4$

Wavenumber / cm^{-1}	Calculated Cross Section / 10^{-21} cm^2
2250.225	0.12
2259.887	0.12
2270.148	0.12
2279.982	0.12
2289.902	0.12
2299.908	0.10
2310.002	0.10
2320.186	0.10
2329.916	0.10
2339.729	0.08
2350.176	0.10
2360.16	0.10
2370.23	0.10
2379.819	0.10
2390.057	0.08
2399.808	0.08
2410.219	0.08
2420.136	0.10
2430.134	0.10
2440.215	0.08

2449.78	0.08
2460.025	0.08
2469.746	0.08
2480.159	0.08
2490.04	0.08
2500	0.08
2510.04	0.08
2520.161	0.08
2529.724	0.08
2540.005	0.10
2549.72	0.08
2560.164	0.08
2570.033	0.08
2579.979	0.08
2590.003	0.08
2600.104	0.08
2610.285	0.08
2619.859	0.08
2630.195	0.08
2639.916	0.08
2649.709	0.08
2660.282	0.08
2670.227	0.08
2680.247	0.10
2690.342	0.10
2699.784	0.10
2710.027	0.10
2720.348	0.10
2730.003	0.10
2739.726	0.10
2750.275	0.14
2760.144	0.12
2770.083	0.14
2780.095	0.12
2790.179	0.16
2800.336	0.16
2809.778	0.18
2820.079	0.16
2829.655	0.16
2840.102	0.20
2849.815	0.20
2859.594	0.24
2870.264	0.28
2880.184	0.41
2890.173	0.45

2900.232	0.45
2910.361	0.57
2919.708	0.65
2929.974	0.73
2940.312	0.90
2949.853	0.92
2960.332	1.00
2970.003	1.10
2979.738	1.36
2990.431	1.40
3000.3	1.51
3030.303	1.55
3040.438	1.55
3049.71	1.85
3059.976	2.06
3070.31	2.30
3079.766	2.44
3090.235	3.09
3099.814	3.68
3110.42	4.86
3120.125	5.58
3129.89	8.51
3139.717	12.19
3149.606	17.38
3159.558	19.49
3169.572	24.52
3179.65	26.35
3189.793	28.75
3200	31.66
3210.273	35.31
3219.575	33.78
3229.974	29.26
3240.441	26.27
3249.919	24.72
3260.515	22.29
3270.111	21.82
3279.764	18.92
3289.474	18.25
3300.33	17.26
3310.162	14.88
3320.053	11.84
3330.003	10.60
3340.013	8.36
3350.084	6.90
3360.215	6.23

3370.408	5.58
3379.52	5.53
3389.831	5.53
3400.204	5.92
3409.478	6.33
3419.973	7.55
3430.532	8.81
3439.972	9.04
3449.465	9.73
3460.208	9.42
3469.813	9.46
3479.471	9.30
3490.401	9.87
3500.175	10.09
3510.004	10.34
3519.887	9.73
3529.827	9.08
3539.823	7.55
3549.876	10.07
3559.986	8.16
3570.154	6.76
3580.38	5.49
3589.375	4.23
3599.712	3.87
3610.108	3.36
3620.565	3.54
3629.764	3.40
3640.335	4.03
3649.635	3.80
3660.322	5.96
3669.725	7.02
3680.53	6.23
3690.037	8.91
3699.593	15.22
3710.575	14.00
3720.238	11.52
3729.952	11.86
3739.716	5.90
3749.531	7.45
3759.399	4.78
3769.318	2.05
3779.289	1.28
3789.314	0.94
3799.392	0.79
3809.524	0.69

3819.71	0.69
3829.95	0.75
3840.246	0.75
3850.597	0.79
3859.514	0.63
3869.969	0.47
3880.481	0.39
3889.537	0.35
3900.156	0.35
3909.304	0.33
3920.031	0.28
3929.273	0.24
3940.11	0.20
3949.447	0.18
3960.396	0.16
3969.829	0.14
3979.308	0.12
3990.423	0.10
4000	0.10

$Zn_2^+(H_2O)_5$

Wavenumber / cm^{-1}	Calculated Cross Section / 10^{-21} cm^2
2250.225	0.31
2259.887	0.37
2270.148	0.37
2279.982	0.37
2289.902	0.37
2299.908	0.37
2310.002	0.31
2320.186	0.31
2329.916	0.31
2339.729	0.31
2350.176	0.31
2360.16	0.31
2370.23	0.31
2379.819	0.31
2390.057	0.31
2399.808	0.31
2410.219	0.31
2420.136	0.31
2430.134	0.31
2440.215	0.31
2449.78	0.31
2460.025	0.31

2469.746	0.24
2480.159	0.31
2490.04	0.24
2500	0.24
2510.04	0.24
2520.161	0.24
2529.724	0.24
2540.005	0.24
2549.72	0.24
2560.164	0.18
2570.033	0.18
2579.979	0.18
2590.003	0.18
2600.104	0.18
2610.285	0.18
2619.859	0.24
2630.195	0.24
2639.916	0.24
2649.709	0.18
2660.282	0.24
2670.227	0.24
2680.247	0.24
2690.342	0.24
2699.784	0.24
2710.027	0.24
2720.348	0.24
2730.003	0.24
2739.726	0.24
2750.275	0.31
2760.144	0.37
2770.083	0.43
2780.095	0.43
2790.179	0.49
2800.336	0.49
2809.778	0.61
2820.079	0.67
2829.655	0.67
2840.102	0.73
2849.815	0.85
2859.594	0.85
2870.264	0.98
2880.184	1.10
2890.173	1.16
2900.232	1.22
2910.361	1.34

2919.708	1.53
2929.974	1.59
2940.312	1.83
2949.853	1.95
2960.332	2.08
2970.003	2.20
2979.738	2.50
2990.431	2.81
3000.3	3.17
3010.235	3.30
3020.236	3.66
3030.303	4.03
3040.438	4.34
3049.71	4.94
3059.976	5.25
3070.31	6.04
3079.766	6.29
3090.235	6.78
3099.814	6.96
3110.42	7.69
3120.125	8.06
3129.89	8.97
3139.717	10.01
3149.606	12.27
3159.558	14.41
3169.572	18.07
3179.65	21.85
3189.793	28.87
3200	38.46
3210.273	43.78
3219.575	48.04
3229.974	49.27
3240.441	49.27
3249.919	47.49
3260.515	46.28
3270.111	46.40
3279.764	45.17
3289.474	45.47
3300.33	43.23
3310.162	37.49
3320.053	32.66
3330.003	26.92
3340.013	23.02
3350.084	21.12
3360.215	18.62

3370.408	16.61
3379.52	15.32
3389.831	14.16
3400.204	14.29
3409.478	14.77
3419.973	15.14
3430.532	15.39
3439.972	16.36
3449.465	17.21
3460.208	18.07
3469.813	18.74
3479.471	20.27
3490.401	21.80
3500.175	21.19
3510.004	19.66
3519.887	15.87
3529.827	19.29
3539.823	18.25
3549.876	16.48
3559.986	14.16
3570.154	11.60
3580.38	9.83
3589.375	8.67
3599.712	7.94
3610.108	7.75
3620.565	6.66
3629.764	6.90
3640.335	10.07
3649.635	12.76
3660.322	9.04
3669.725	9.64
3680.53	17.04
3690.037	15.81
3699.593	11.72
3710.575	16.91
3720.238	11.42
3729.952	13.49
3739.716	13.61
3749.531	6.78
3759.399	4.46
3769.318	3.30
3779.289	2.87
3789.314	2.81
3799.392	2.93
3809.524	2.93

3819.71	3.11
3829.95	3.42
3840.246	2.32
3850.597	1.65
3859.514	1.34
3869.969	1.10
3880.481	0.98
3889.537	0.92
3900.156	0.73
3909.304	0.61
3920.031	0.55
3929.273	0.49
3940.11	0.43
3949.447	0.37
3960.396	0.31
3969.829	0.31
3979.308	0.31
3990.423	0.24
4000	0.31

$Zn^{2+}(H_2O)_6$

Wavenumber / cm^{-1}	Calculated Cross Section / 10^{-21} cm^2
2250.225	0.73
2259.887	0.73
2270.148	0.73
2279.982	0.79
2289.902	0.85
2299.908	0.79
2310.002	0.79
2320.186	0.73
2329.916	0.79
2339.729	0.73
2350.176	0.73
2360.16	0.73
2370.23	0.67
2379.819	0.73
2390.057	0.73
2399.808	0.73
2410.219	0.73
2420.136	0.79
2430.134	0.79
2440.215	0.73
2449.78	0.79
2460.025	0.73

2469.746	0.67
2480.159	0.67
2490.04	0.67
2500	0.67
2510.04	0.61
2520.161	0.55
2529.724	0.55
2540.005	0.55
2549.72	0.49
2560.164	0.49
2570.033	0.49
2579.979	0.49
2590.003	0.49
2600.104	0.43
2610.285	0.43
2619.859	0.43
2630.195	0.43
2639.916	0.43
2649.709	0.43
2660.282	0.43
2670.227	0.43
2680.247	0.43
2690.342	0.43
2699.784	0.49
2710.027	0.43
2720.348	0.49
2730.003	0.49
2739.726	0.49
2750.275	0.49
2760.144	0.55
2770.083	0.55
2780.095	0.55
2790.179	0.55
2800.336	0.61
2809.778	0.67
2820.079	0.67
2829.655	0.67
2840.102	0.73
2849.815	0.79
2859.594	0.85
2870.264	0.92
2880.184	1.04
2890.173	1.22
2900.232	1.40
2910.361	1.59

2919.708	1.77
2929.974	2.14
2940.312	2.56
2949.853	2.87
2960.332	3.48
2970.003	3.60
2979.738	3.85
2990.431	4.15
3000.3	4.09
3010.235	4.09
3020.236	4.21
3030.303	4.21
3040.438	4.40
3049.71	4.58
3059.976	4.70
3070.31	5.07
3079.766	5.31
3090.235	5.56
3099.814	6.17
3110.42	6.59
3120.125	7.02
3129.89	7.39
3139.717	7.75
3149.606	8.73
3159.558	9.89
3169.572	11.72
3179.65	13.86
3189.793	17.21
3200	22.47
3210.273	28.39
3219.575	31.31
3229.974	33.33
3240.441	34.56
3249.919	34.61
3260.515	35.84
3270.111	37.12
3279.764	38.46
3289.474	43.76
3300.33	50.25
3310.162	56.29
3320.053	58.36
3330.003	54.27
3340.013	50.79
3350.084	47.38
3360.215	42.31

3370.408	37.79
3379.52	34.18
3389.831	29.85
3400.204	27.29
3409.478	25.58
3419.973	25.03
3430.532	25.16
3439.972	26.19
3449.465	25.39
3460.208	23.75
3469.813	23.20
3479.471	22.28
3490.401	21.74
3500.175	19.54
3510.004	16.97
3519.887	13.49
3529.827	16.97
3539.823	17.52
3549.876	16.12
3559.986	13.31
3570.154	11.23
3580.38	9.52
3589.375	8.06
3599.712	6.84
3610.108	6.29
3620.565	5.56
3629.764	5.49
3640.335	8.30
3649.635	15.02
3660.322	10.87
3669.725	13.49
3680.53	24.24
3690.037	29.30
3699.593	19.23
3710.575	15.94
3720.238	12.94
3729.952	14.16
3739.716	18.56
3749.531	10.07
3759.399	6.84
3769.318	5.43
3779.289	4.70
3789.314	4.64
3799.392	4.46
3809.524	4.27

3819.71	4.15
3829.95	4.58
3840.246	2.81
3850.597	2.14
3859.514	1.83
3869.969	1.47
3880.481	1.22
3889.537	1.04
3900.156	0.85
3909.304	0.79
3920.031	0.73
3929.273	0.67
3940.11	0.61
3949.447	0.49
3960.396	0.55
3969.829	0.49
3979.308	0.49
3990.423	0.49
4000	0.55

$Zn_2^+(H_2O)_7$

Wavenumber / cm^{-1}	Calculated Cross Section / 10^{-21} cm^2
2250.225	0.95
2259.887	0.85
2270.148	0.82
2279.982	0.89
2289.902	0.79
2299.908	0.79
2310.002	0.85
2320.186	0.70
2329.916	0.70
2339.729	0.70
2350.176	0.67
2360.16	0.73
2370.23	0.70
2379.819	0.70
2390.057	0.70
2399.808	0.70
2410.219	0.67
2420.136	0.67
2430.134	0.70
2440.215	0.67
2449.78	0.70
2460.025	0.67

2469.746	0.61
2480.159	0.64
2490.04	0.61
2500	0.58
2510.04	0.64
2520.161	0.58
2529.724	0.55
2540.005	0.55
2549.72	0.55
2560.164	0.52
2570.033	0.52
2579.979	0.52
2590.003	0.52
2600.104	0.52
2610.285	0.52
2619.859	0.49
2630.195	0.52
2639.916	0.49
2649.709	0.49
2660.282	0.49
2670.227	0.52
2680.247	0.52
2690.342	0.49
2699.784	0.49
2710.027	0.49
2720.348	0.49
2730.003	0.49
2739.726	0.49
2750.275	0.52
2760.144	0.49
2770.083	0.49
2780.095	0.52
2790.179	0.55
2800.336	0.52
2809.778	0.58
2820.079	0.61
2829.655	0.58
2840.102	0.64
2849.815	0.67
2859.594	0.67
2870.264	0.70
2880.184	0.79
2890.173	0.79
2900.232	0.85
2910.361	0.95

2919.708	0.92
2929.974	1.07
2940.312	1.13
2949.853	1.31
2960.332	1.53
2970.003	1.71
2979.738	2.01
2990.431	2.35
3000.3	2.75
3010.235	3.11
3020.236	3.51
3030.303	3.82
3040.438	4.06
3049.71	4.18
3059.976	4.36
3070.31	4.61
3079.766	4.64
3090.235	4.88
3099.814	5.13
3110.42	5.59
3120.125	5.83
3129.89	6.47
3139.717	6.71
3149.606	7.39
3159.558	8.21
3169.572	9.67
3179.65	10.62
3189.793	12.54
3200	15.45
3210.273	16.61
3219.575	17.24
3229.974	16.76
3240.441	17.49
3249.919	17.55
3260.515	17.98
3270.111	19.02
3279.764	19.23
3289.474	20.45
3300.33	21.37
3310.162	23.69
3320.053	27.41
3330.003	31.50
3340.013	36.69
3350.084	41.18
3360.215	41.39

3370.408	40.48
3379.52	39.40
3389.831	36.26
3400.204	31.93
3409.478	28.93
3419.973	26.07
3430.532	25.25
3439.972	24.94
3449.465	25.06
3460.208	24.48
3469.813	23.87
3479.471	22.65
3490.401	22.32
3500.175	20.21
3510.004	17.95
3519.887	14.62
3529.827	17.70
3539.823	17.70
3549.876	16.45
3559.986	14.19
3570.154	12.39
3580.38	10.50
3589.375	9.03
3599.712	7.51
3610.108	6.20
3620.565	5.16
3629.764	4.76
3640.335	5.83
3649.635	9.80
3660.322	6.81
3669.725	8.64
3680.53	18.07
3690.037	28.85
3699.593	24.02
3710.575	16.76
3720.238	12.15
3729.952	10.99
3739.716	12.36
3749.531	7.60
3759.399	5.62
3769.318	4.36
3779.289	3.72
3789.314	3.48
3799.392	3.20
3809.524	3.17

3819.71	2.99
3829.95	2.81
3840.246	2.14
3850.597	1.62
3859.514	1.40
3869.969	1.13
3880.481	0.98
3889.537	0.89
3900.156	0.79
3909.304	0.67
3920.031	0.64
3929.273	0.55
3940.11	0.55
3949.447	0.55
3960.396	0.52
3969.829	0.52
3979.308	0.52
3990.423	0.52
4000	0.55

$Zn_2^+(H_2O)_8$

Wavenumber / cm^{-1}	Calculated Cross Section / 10^{-21} cm^2
2250.225	0.79
2259.887	0.73
2270.148	0.76
2279.982	0.61
2289.902	0.64
2299.908	0.58
2310.002	0.67
2320.186	0.61
2329.916	0.55
2339.729	0.58
2350.176	0.61
2360.16	0.55
2370.23	0.61
2379.819	0.58
2390.057	0.55
2399.808	0.55
2410.219	0.52
2420.136	0.52
2430.134	0.52
2440.215	0.52
2449.78	0.49
2460.025	0.49

2469.746	0.52
2480.159	0.49
2490.04	0.40
2500	0.40
2510.04	0.37
2520.161	0.40
2529.724	0.43
2540.005	0.40
2549.72	0.46
2560.164	0.37
2570.033	0.31
2579.979	0.37
2590.003	0.37
2600.104	0.34
2610.285	0.34
2619.859	0.37
2630.195	0.31
2639.916	0.31
2649.709	0.31
2660.282	0.34
2670.227	0.37
2680.247	0.34
2690.342	0.34
2699.784	0.40
2710.027	0.34
2720.348	0.37
2730.003	0.34
2739.726	0.37
2750.275	0.40
2760.144	0.37
2770.083	0.34
2780.095	0.37
2790.179	0.43
2800.336	0.37
2809.778	0.43
2820.079	0.43
2829.655	0.40
2840.102	0.43
2849.815	0.49
2859.594	0.55
2870.264	0.52
2880.184	0.52
2890.173	0.61
2900.232	0.70
2910.361	0.67

2919.708	0.79
2929.974	0.79
2940.312	0.98
2949.853	1.04
2960.332	1.13
2970.003	1.22
2979.738	1.22
2990.431	1.40
3000.3	1.65
3010.235	1.92
3020.236	2.17
3030.303	2.53
3040.438	2.75
3049.71	3.17
3059.976	3.54
3070.31	4.03
3079.766	4.33
3090.235	4.98
3099.814	5.34
3110.42	6.17
3120.125	6.78
3129.89	7.39
3139.717	8.09
3149.606	9.25
3159.558	10.35
3169.572	12.03
3179.65	13.92
3189.793	16.09
3200	18.59
3210.273	18.62
3219.575	18.59
3229.974	19.02
3240.441	18.56
3249.919	18.59
3260.515	18.35
3270.111	18.86
3279.764	18.50
3289.474	18.80
3300.33	19.35
3310.162	19.72
3320.053	21.12
3330.003	23.23
3340.013	25.64
3350.084	30.49
3360.215	35.32

3370.408	40.17
3379.52	41.87
3389.831	41.79
3400.204	42.00
3409.478	38.97
3419.973	38.22
3430.532	36.76
3439.972	35.99
3449.465	35.49
3460.208	34.89
3469.813	33.21
3479.471	32.75
3490.401	31.05
3500.175	28.14
3510.004	24.33
3519.887	18.89
3529.827	22.62
3539.823	22.37
3549.876	20.79
3559.986	18.68
3570.154	15.90
3580.38	13.31
3589.375	11.48
3599.712	9.34
3610.108	7.66
3620.565	6.14
3629.764	5.28
3640.335	5.59
3649.635	8.00
3660.322	6.14
3669.725	7.08
3680.53	18.04
3690.037	34.00
3699.593	28.72
3710.575	20.33
3720.238	16.21
3729.952	12.15
3739.716	11.63
3749.531	7.57
3759.399	5.40
3769.318	4.12
3779.289	3.48
3789.314	2.99
3799.392	2.72
3809.524	2.69

3819.71	2.44
3829.95	2.32
3840.246	1.83
3850.597	1.50
3859.514	1.25
3869.969	0.98
3880.481	0.95
3889.537	0.73
3900.156	0.67
3909.304	0.61
3920.031	0.49
3929.273	0.49
3940.11	0.43
3949.447	0.43
3960.396	0.40
3969.829	0.43
3979.308	0.52
3990.423	0.43
4000	0.43

$Zn_2^+(H_2O)_{10}$

Wavenumber / cm^{-1}	Calculated Cross Section / 10^{-21} cm^2
2250.225	1.40
2259.887	1.53
2270.148	1.47
2279.982	1.59
2289.902	1.59
2299.908	1.65
2310.002	1.53
2320.186	1.47
2329.916	1.53
2339.729	1.40
2350.176	1.47
2360.16	1.47
2370.23	1.40
2379.819	1.47
2390.057	1.40
2399.808	1.47
2410.219	1.34
2420.136	1.47
2430.134	1.34
2440.215	1.34
2449.78	1.40

2460.025	1.40
2469.746	1.28
2480.159	1.40
2490.04	1.28
2500	1.34
2510.04	1.40
2520.161	1.34
2529.724	1.16
2540.005	1.16
2549.72	1.10
2560.164	1.28
2570.033	1.28
2579.979	1.22
2590.003	1.16
2600.104	1.16
2610.285	1.16
2619.859	1.16
2630.195	1.04
2639.916	1.10
2649.709	1.04
2660.282	1.22
2670.227	1.16
2680.247	1.04
2690.342	1.16
2699.784	0.98
2710.027	1.16
2720.348	1.16
2730.003	1.16
2739.726	1.10
2750.275	1.28
2760.144	1.22
2770.083	1.16
2780.095	1.16
2790.179	1.28
2800.336	1.10
2809.778	1.16
2820.079	1.34
2829.655	1.22
2840.102	1.40
2849.815	1.34
2859.594	1.53
2870.264	1.40
2880.184	1.53
2890.173	1.53
2900.232	1.59

2910.361	1.83
2919.708	1.71
2929.974	2.01
2940.312	2.14
2949.853	2.14
2960.332	2.38
2970.003	2.56
2979.738	2.69
2990.431	3.11
3000.3	3.17
3010.235	3.42
3020.236	3.79
3030.303	4.15
3040.438	4.40
3049.71	5.01
3059.976	5.37
3070.31	6.04
3079.766	6.41
3090.235	7.02
3099.814	7.69
3110.42	8.43
3120.125	9.10
3129.89	10.07
3139.717	11.35
3149.606	12.64
3159.558	14.16
3169.572	16.73
3179.65	18.74
3189.793	22.46
3200	26.13
3210.273	28.88
3219.575	31.56
3229.974	31.07
3240.441	30.71
3249.919	30.40
3260.515	29.31
3270.111	34.19
3279.764	32.72
3289.474	32.66
3300.33	31.93
3310.162	33.03
3320.053	33.58
3330.003	34.61
3340.013	37.42
3350.084	45.67

3360.215	52.20
3370.408	56.48
3379.52	59.45
3389.831	61.35
3400.204	64.11
3409.478	68.43
3419.973	74.48
3430.532	77.42
3439.972	78.45
3449.465	79.11
3460.208	77.05
3469.813	78.87
3479.471	79.23
3490.401	77.05
3500.175	69.23
3510.004	60.07
3519.887	47.56
3529.827	54.70
3539.823	53.11
3549.876	46.46
3559.986	38.46
3570.154	30.53
3580.38	23.93
3589.375	20.08
3599.712	16.91
3610.108	14.22
3620.565	10.87
3629.764	9.46
3640.335	7.63
3649.635	9.71
3660.322	7.51
3669.725	8.18
3680.53	25.95
3690.037	60.50
3699.593	51.95
3710.575	35.47
3720.238	28.08
3729.952	16.06
3739.716	16.05
3749.531	9.89
3759.399	7.45
3769.318	5.25
3779.289	4.33
3789.314	3.85
3799.392	3.54

3809.524	3.30
3819.71	3.17
3829.95	3.17
3840.246	2.50
3850.597	2.26
3859.514	2.01
3869.969	1.65
3880.481	1.83
3889.537	1.47
3900.156	1.34
3909.304	1.53
3920.031	1.40
3929.273	1.22
3940.11	1.04
3949.447	1.10
3960.396	1.28
3969.829	1.40
3979.308	1.40
3990.423	1.16
4000	1.22

$Zn^{2+}(H_2O)_{16}$

Wavenumber / cm^{-1}	Calculated Cross Section / 10^{-21} cm^2
2250.225	3.17
2259.887	2.62
2270.148	2.08
2279.982	2.01
2289.902	1.59
2299.908	1.59
2310.002	1.53
2320.186	1.53
2329.916	1.47
2339.729	1.53
2350.176	1.40
2360.16	1.53
2370.23	1.59
2379.819	1.53
2390.057	1.53
2399.808	1.47
2410.219	1.40
2420.136	1.71
2430.134	1.34
2440.215	1.34
2449.78	1.59

2460.025	1.83
2469.746	1.65
2480.159	1.65
2490.04	1.77
2500	1.59
2510.04	1.77
2520.161	1.89
2529.724	1.59
2540.005	1.53
2549.72	1.95
2560.164	1.28
2570.033	1.83
2579.979	1.40
2590.003	1.22
2600.104	1.28
2610.285	1.59
2619.859	1.16
2630.195	1.34
2639.916	1.47
2649.709	1.53
2660.282	0.06
2670.227	1.10
2680.247	1.47
2690.342	1.47
2699.784	1.53
2710.027	1.34
2720.348	1.34
2730.003	1.28
2739.726	1.47
2750.275	1.40
2760.144	1.53
2770.083	1.53
2780.095	1.59
2790.179	1.65
2800.336	1.59
2809.778	1.83
2820.079	1.77
2829.655	1.65
2840.102	1.89
2849.815	2.08
2859.594	2.26
2870.264	1.95
2880.184	2.69
2890.173	2.81
2900.232	2.87

2910.361	3.17
2919.708	3.48
2929.974	3.91
2940.312	4.40
2949.853	4.58
2960.332	5.31
2970.003	5.62
2979.738	6.35
2990.431	7.82
3000.3	8.30
3010.235	9.59
3020.236	10.99
3030.303	12.52
3040.438	13.92
3049.71	15.32
3059.976	16.18
3070.31	17.52
3079.766	18.86
3090.235	20.45
3099.814	21.30
3110.42	22.35
3120.125	23.69
3129.89	25.27
3139.717	27.84
3149.606	28.93
3159.558	30.46
3169.572	36.99
3179.65	40.35
3189.793	43.04
3200	48.59
3210.273	52.87
3219.575	57.13
3229.974	55.74
3240.441	54.04
3249.919	52.13
3260.515	49.46
3270.111	45.48
3279.764	43.95
3289.474	45.54
3300.33	45.49
3310.162	45.67
3320.053	47.37
3330.003	45.79
3340.013	46.34
3350.084	48.29

3360.215	46.09
3370.408	50.98
3379.52	53.17
3389.831	52.80
3400.204	56.23
3409.478	56.10
3419.973	57.93
3430.532	64.60
3439.972	68.86
3449.465	68.12
3460.208	71.13
3469.813	64.46
3479.471	65.37
3490.401	69.54
3500.175	61.72
3510.004	57.81
3519.887	45.85
3529.827	52.19
3539.823	52.13
3549.876	45.30
3559.986	38.22
3570.154	34.56
3580.38	33.89
3589.375	32.23
3599.712	28.51
3610.108	23.81
3620.565	17.65
3629.764	13.86
3640.335	11.54
3649.635	8.67
3660.322	7.20
3669.725	8.00
3680.53	13.13
3690.037	40.72
3699.593	43.28
3710.575	27.78
3720.238	29.49
3729.952	16.12
3739.716	9.22
3749.531	6.53
3759.399	4.58
3769.318	4.09
3779.289	2.69
3789.314	2.44
3799.392	1.89

3809.524	1.77
3819.71	1.71
3829.95	1.59
3840.246	1.59
3850.597	1.77
3859.514	1.46
3869.969	1.71
3880.481	0.06
3889.537	0.06
3900.156	0.06
3909.304	0.06
3920.031	0.06
3929.273	0.06
3940.11	0.06
3949.447	0.06
3960.396	0.06
3969.829	0.06
3979.308	0.06
3990.423	0.06
4000	1.22

$Zn^{2+}(H_2O)_{20}$

Wavenumber / cm^{-1}	Calculated Cross Section / 10^{-21} cm^2
2250.225	3.79
2259.887	4.15
2270.148	4.09
2279.982	4.09
2289.902	3.72
2299.908	3.91
2310.002	3.17
2320.186	3.30
2329.916	3.54
2339.729	3.60
2350.176	3.85
2360.16	3.85
2370.23	3.85
2379.819	3.54
2390.057	3.97
2399.808	4.03
2410.219	2.38
2420.136	3.48
2430.134	3.79
2440.215	3.91

2449.78	3.78
2460.025	3.85
2469.746	3.66
2480.159	4.09
2490.04	3.48
2500	3.42
2510.04	3.60
2520.161	3.72
2529.724	3.48
2540.005	3.24
2549.72	3.66
2560.164	3.79
2570.033	3.48
2579.979	2.20
2590.003	3.60
2600.104	3.72
2610.285	3.54
2619.859	3.11
2630.195	3.05
2639.916	3.17
2649.709	3.48
2660.282	3.17
2670.227	3.17
2680.247	2.01
2690.342	2.81
2699.784	2.93
2710.027	3.11
2720.348	3.60
2730.003	3.42
2739.726	3.48
2750.275	2.99
2760.144	3.36
2770.083	3.30
2780.095	3.05
2790.179	2.93
2800.336	3.97
2809.778	3.42
2820.079	3.79
2829.655	3.78
2840.102	4.64
2849.815	3.60
2859.594	4.39
2870.264	4.88
2880.184	4.95
2890.173	6.29

2900.232	5.92
2910.361	6.96
2919.708	7.63
2929.974	9.46
2940.312	8.98
2949.853	10.44
2960.332	10.62
2970.003	11.72
2979.738	11.96
2990.431	13.37
3000.3	14.71
3010.235	17.22
3020.236	18.62
3030.303	21.19
3040.438	23.26
3049.71	23.87
3059.976	25.34
3070.31	27.29
3079.766	28.39
3090.235	29.00
3099.814	32.23
3110.42	32.85
3120.125	35.35
3129.89	35.59
3139.717	37.85
3149.606	42.24
3159.558	44.93
3169.572	48.10
3179.65	50.66
3189.793	55.61
3200	58.97
3210.273	57.57
3219.575	63.91
3229.974	61.23
3240.441	58.07
3249.919	54.45
3260.515	54.34
3270.111	52.75
3279.764	50.73
3289.474	53.59
3300.33	54.16
3310.162	52.57
3320.053	53.91
3330.003	50.30
3340.013	53.85

3350.084	57.39
3360.215	56.11
3370.408	58.61
3379.52	60.80
3389.831	61.47
3400.204	64.65
3409.478	66.84
3419.973	67.76
3430.532	68.69
3439.972	72.46
3449.465	70.68
3460.208	72.77
3469.813	70.51
3479.471	72.51
3490.401	70.76
3500.175	68.26
3510.004	58.67
3519.887	48.65
3529.827	55.37
3539.823	56.04
3549.876	51.34
3559.986	47.56
3570.154	50.12
3580.38	49.64
3589.375	44.99
3599.712	37.30
3610.108	29.12
3620.565	21.37
3629.764	17.95
3640.335	11.84
3649.635	10.74
3660.322	10.56
3669.725	7.75
3680.53	11.30
3690.037	38.52
3699.593	49.44
3710.575	26.80
3720.238	22.35
3729.952	14.04
3739.716	8.49
3749.531	5.01
3759.399	4.03
3769.318	4.15
3779.289	4.39
3789.314	3.72

3799.392	3.66
3809.524	2.44
3819.71	1.65
3829.95	1.59
3840.246	2.20
3850.597	3.72
3859.514	3.17
3869.969	1.47
3880.481	0.06
3889.537	1.53
3900.156	2.93
3909.304	1.46
3920.031	2.75
3929.273	2.50
3940.11	1.77
3949.447	0.06
3960.396	1.47
3969.829	1.59
3979.308	2.14
3990.423	3.24
4000	1.65

References

1. Bersenkowitsch, N. K.; Ončák, M.; Heller, J.; van der Linde, C.; Beyer, M. K. Photodissociation of Sodium Iodide Clusters Doped with Small Hydrocarbons *Chem. Eur. J.* **2018**, *24*, 12433.
2. Heller, J.; Ončák, M.; Bersenkowitsch, N.K.; van der Linde, C.; Beyer, M. K. Infrared multiple photon dissociation of cesium iodide clusters doped with mono-, di- and triglycine *Eur. J. Mass Spectrom*. **2019**, *25*, 122–132.
3. Cunningham, E. M.; Taxer, T.; Heller, J.; Ončák, M.; van der Linde, C.; Beyer, M. K. Microsolvation of Zn cations: infrared multiple photon dissociation spectroscopy of Zn+(H2O)n (n = 2–35)† *Phys. Chem. Chem. Phys.* **2021**, *23*, 3627.
